# Investigation of *in silico* studies for cytochrome P450 isoforms specificity

**DOI:** 10.1016/j.csbj.2024.08.002

**Published:** 2024-08-05

**Authors:** Yao Wei, Luca Palazzolo, Omar Ben Mariem, Davide Bianchi, Tommaso Laurenzi, Uliano Guerrini, Ivano Eberini

**Affiliations:** Dipartimento di Scienze Farmacologiche e Biomolecolari “Rodolfo Paoletti”, Università degli Studi di Milano, Via Giuseppe Balzaretti 9, 20133 Milano, Italy

**Keywords:** Cytochrome P450, Computational biochemistry, Machine learning, Inhibitor, Substrate, Drug metabolism

## Abstract

Cytochrome P450 (CYP450) enzymes comprise a highly diverse superfamily of heme-thiolate proteins that responsible for catalyzing over 90 % of enzymatic reactions associated with xenobiotic metabolism in humans. Accurately predicting whether chemicals are substrates or inhibitors of different CYP450 isoforms can aid in pre-selecting hit compounds for the drug discovery process, chemical toxicology studies, and patients treatment planning. In this work, we investigated *in silico* studies on CYP450s specificity over past twenty years, categorizing these studies into structure-based and ligand-based approaches. Subsequently, we utilized 100 of the most frequently prescribed drugs to test eleven machine learning-based prediction models which were published between 2015 and 2024. We analyzed various aspects of the evaluated models, such as their datasets, algorithms, and performance. This will give readers with a comprehensive overview of these prediction models and help them choose the most suitable one to do prediction. We also provide our insights for future research trend in both structure-based and ligand-based approaches in this field.

## Graphical Abstract

**Figure:**
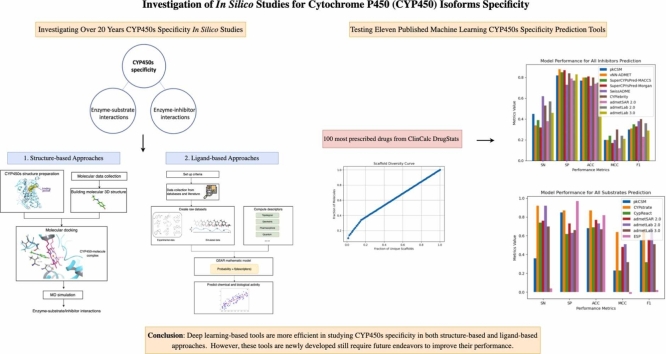

## 1 Introduction

Xenobiotics are exogenous chemical compounds to which the body is exposed, including those found in drugs, food and environmental pollutants. After xenobiotic intake, they undergo the kinetic processes of absorption, distribution, metabolism, and excretion (ADME). These processes can sometimes lead to intermediary metabolism disorders and toxic effects [1]. Drugs are a significant subclass of xenobiotic substances. When orally administered, several drugs undergo the hepatic "first-pass effect" before entering the systemic blood circulation. Various liver enzymes catalyze metabolic reactions that convert the non-polar compound (parent drug) into more hydrophilic metabolites. These metabolic reactions are divided into two phases: phase I (involving oxidation, reduction, hydrolysis), and phase II (involving conjugation). During phase I, the parent drug undergoes chemical modifications, such as the introduction of reactive or polar groups (e.g., -SH or -OH), yielding more polar metabolites, which can be pharmacologically inactive or active. In phase II, xenobiotics or their phase I metabolites can be conjugated with hydrophilic endogenous species, such as glutathione or glycine, further enhancing their solubility and facilitating their excretion, primarily through the kidneys [2].

Cytochrome P450 (CYP450) enzymes comprise a highly diverse superfamily of heme-thiolate proteins [3] that serve as indispensable components of the oxidative metabolic machinery and play a pivotal role in the metabolism and detoxification of a wide range of xenobiotics. They are found across various life forms, including animals, plants, bacteria, viruses, and more [4]. Notably, CYP450 enzymes are the major drug-metabolizing phase I enzymes of the liver [5] and are responsible for catalyzing over 90 % of enzymatic reactions associated with xenobiotic metabolism [6]. In the human context, a total of 57 distinct CYP450 isoforms have been identified to date. These enzymes predominantly reside in the membranes of the endoplasmic reticulum and mitochondria in hepatocytes [7]. The three human CYP450 subfamilies (CYP1–3) are typically participating in the xenobiotic metabolism, like drugs, while the remaining human CYP subfamilies are usually engaged in the endobiotic one [8]. Among the CYP450 enzymes, six isoforms stand out as particularly critical for drug metabolism in humans, including CYP1A2, CYP2C9, CYP2C19, CYP2D6, CYP2E1 and CYP3A4. These enzymes are responsible for metabolizing a substantial portion of clinically administered medications, accounting for approximately 70 % to 80 % of clinically relevant drugs [5], [9].

**Fig. 1:**
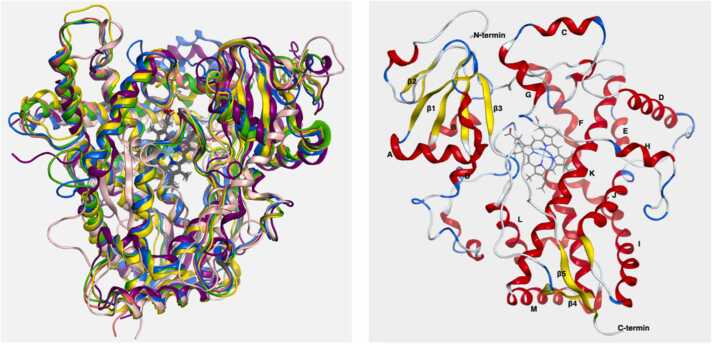
Three-dimensional structure of CYP450s. Left: Superposition of CYP1A2 (pink; PDB: 2HI4 [12]), CYP2C9 (green; PDB: 4NZ2 [13]), CYP2C19 (light coral, PDB: 4GQS [14]), CYP2D6 (blue, PDB: 4WNV [15]), CYP2E1 (yellow, PDB: 3GPH [16]), and CYP3A4 (purple, PDB: 1TQN [17]). Right: Three-dimensional structure of CYP3A4 (PDB: 1TQN). Thirteen α-helices are labelled from A to M. Five β-sheets are represented in β1-β5. All the heme cofactors are represented as gray sticks. (Protein structures were displayed by MOE 2022.02 [18]).

Human CYP450s consist of 400–500 amino acid residues, and exhibit a helix-rich secondary structure architecture and an enclosed active site. The secondary structure elements include 13 α-helices and 2–5 β-sheets [10], [11]. CYP450s contain a heme cofactor that is essential for the catalytic reaction to occur. The heme cofactor is located at the bottom of the active site, with the heme iron fifth coordination position bound to a cysteine thiolate, and the sixth coordination position free to perform redox reactions [5]. CYP450 structures exhibit a similar shape resembling an inverted triangle and are highly conserved (Fig. 1, left). Among all the human CYP450s, CYP3A4 is the most prevalent one, and it is responsible for the largest fraction of chemical metabolism [6]. Therefore, we chose CYP3A4 to depict the CYP450s structure (Fig. 1, right).

Pharmaceuticals with metabolic liabilities refer to the susceptibility of a drug to undergo metabolic processes in the body. This can give rise to various issues, such as improper drug metabolism, drug-drug interactions (DDIs) and drug-induced toxicity, often linked to CYP450 inhibition or induction [19]. Therefore, recognizing the significance of CYP450s in drug metabolism is imperative to conduct research aimed at accurately understanding CYP450 specificity. This is crucial for designing novel drug molecules and establishing personalized drug treatment regimens. CYP450 specificity prediction involves anticipating enzyme-substrate interactions and enzyme-inhibitor interactions. This predictive capability can assist in the assessment of metabolic stability, DDIs, and more. However, determining the CYP450 isoforms specificity experimentally faces time- and resource-consuming challenges [20]. Computational techniques can speed up the prediction of CYP450 specificity. These techniques are based on two main approaches: structure-based and ligand-based approaches. The former one relies on the available three-dimensional (3D) protein structures to directly assess the interactions between CYP450s and chemicals. On the other hand, ligand-based approaches can evaluate the structural similarities between ligands and known substrates [20], [21]. From classical molecular modeling to machine learning techniques, computational prediction models can facilitate the study of CYP450 enzymatic interactions at the atomistic level. *In silico* approaches will aid in saving on the experimental costs, accelerating the drug development process, and reducing environmental pollution.

In this review, we investigated *in silico* studies on CYP450s specificity prediction carried out over the past 20 years. Firstly, we summarized both structure-based and ligand-based approaches in CYP450s specificity studies. Machine learning methods face the challenge of the model prediction results not being as accurate as the real experimental results [22]. However, one relevant review discussed the CYP450s inhibitor prediction models and just analyzed the performance of these prediction models as reported in their original publications [23]. Another recently published review article tested the performance of only three open-access CYP450s inhibitors prediction tools [24]. We then extended our scope and evaluations to a set of 100 of the most prescribed drugs to assess eleven predictive tools published between 2015 and 2024, developed using various classical machine learning or deep learning methods. This will give readers, especially researchers using prediction tools for initial investigations of their molecules of interest, as well as people working in pharmacotherapy to predict drug-drug interactions (DDIs) and optimize patients’ treatment plan, a comprehensive overview of the performance of tested machine learning CYP450s specificity prediction models compared to the real experimental or simulation results. We then discuss the advantages and limitations of the evaluated models and provide guidance for selecting appropriate computational tools for prediction. Finally, we highlight trends in the future development of CYP450 specificity prediction models.

## 2 *In silico* prediction tools

### 2.1 Structure-based approaches

**Fig. 2:**
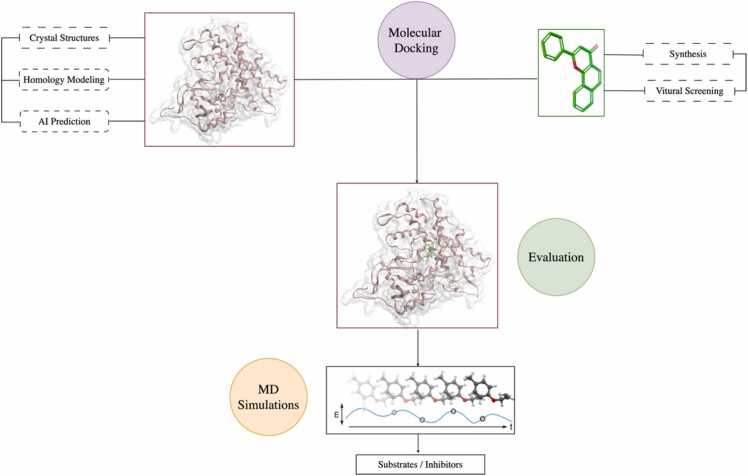
General workflow of structure-based approaches. Enzyme structures can be obtained through experimental crystallization, homology modeling, and AI prediction. Chemicals can be collected via experimental synthesis and virtual screening. Molecular docking is performed to obtain the enzyme-molecule complex. After evaluating the complex structures, the best structures could be submitted to MD simulations to study the enzyme-molecule interaction in a dynamic process.

Structure-based approaches are known for their high accuracy and strong ability to elucidate the dynamic processes of enzyme-ligand interactions and rational binding events at the atomic level. These approaches rely on the 3D structure of proteins, including both experimentally solved structures and theoretical models obtained through techniques such as homology modelling or predictions made by artificial intelligence tools like AlphaFold [25]. Structure-based approaches are implemented through techniques such as molecular docking, molecular dynamics (MD) simulations and quantum mechanical methods. The general workflow of structure-based approaches is summarized in Fig. 2. After preparing the protein and ligand structures, molecular docking is performed to determine the binding poses of the ligand at the enzyme active site. Subsequently, MD simulations can be conducted to study the binding modes during the dynamic process and to verify the stability of the protein-ligand complexes [26]. Additionally, quantum mechanics can be used to optimize the geometry of the enzyme and molecule, as well as to calculate the non-covalent interaction parameters between the ligand and the enzyme cofactor heme [27].

Molecular docking lies at the heart of structure-based approaches. It involves simulating molecular recognition on a computer, generating a series of putative protein-ligand configurations. The docking algorithm and scoring function are the two essential components of molecular docking tools. Protein-ligand interaction represents a thermodynamic equilibrium and is determined by calculating the free energy variation throughout the docking process. By using a scoring function, ligand poses can be assessed based on their approximated binding free energy, allowing for the selection of the most stable one [28].

GOLD, AutoDock, Glide and MOE are the four most widely used molecular docking software [29]. GOLD is a genetic algorithm (GA)-based automated docking program [30]. It can handle fully flexible ligand conformations and partially flexible protein conformations by encoding the conformation information into the corresponding binary strings. Its available scoring functions include CHEMPLP, GoldScore, ChemScore, ASP [31]. Kemp et al. utilized GOLDv2.0 to dock drug-like compounds and build an in-silico tool for predicting inhibitors of CYP2D6. This approach can discriminate between tight and weak binding compounds and predict novel inhibitors compared to experimental and published data [32]. Autodock leverages Lamarckian genetic algorithm (LGA), which integrates local search and genetic algorithms by converting genotypes into phenotypes through developmental mapping. The binding affinity is then assessed through semi-empirical free energy calculations [33], [34]. Hu et al. conducted molecular docking to study the interaction between CYP2E1 and dioxin-like polychlorinated biphenyls (DL-PCBs) using AutoDock 4.2. The docking results were evaluated based on the average binding energy score, the numbers in each cluster, and the distance between the ligand and the ferric ion. The results showed the docking simulations and experimental finding are consistent [35]. Glide is an exhaustive searching-based docking program that comprehensively searches the conformation, orientation, and positional space of the docked ligand. It then refines the binding poses through Monte Carlo sampling. The docking results are ranked by GlideScore, which was expanded from ChemScore [36]. Kesharwani et al. used Glide docking to determine the substrate specificity among CYP1A1, CYP1A2 and CYP1B1, and evaluated their binding affinity through the Glide docking scores. Their docking results were further verified by MD simulations with molecular mechanics Poisson-Boltzmann surface area (MM-PBSA) analysis, and they were compared to the experimental results [37]. MOE is a molecular modelling suite. The MOE-Docking module places the ligand into the protein binding pocket using geometric methods and assesses the docking poses via different scoring functions, including, for example, the GBVI/WSA dG [38]. Metruccio et al. investigated the ligand binding mode in the active sites of CYP26A1, CYP26B1 and CYP26C1 using the Triangle Matcher algorithm of MOE-Docking. They then sorted the binding poses using the London dG empirical function. All the docking results are comparable to the original structures [39].

It is worth noting that in recent years, some docking programs based on deep learning methods have been developed. GNINA [40], a molecular docking software forked from AutoDock Vina [41] and SMINA [42], utilizes Monte Carlo sampling to explore the ligand conformational space and employ convolutional neural networks (CNN) to score and refine the docking poses. GNINA has been used for docking inhibitors to the binding pocket of the CYP4F11, which 3D model was generated by AlphaFold. The top-scoring protein-ligand complexes were refined by MD simulation using AMBER. The resulting CYP4F11-inhibitor complexes are in agreement with the experimental spectroscopic ligand binding assays [43]. DiffDock [44] employed a diffusion generative model on a non-Euclidean manifold of ligand poses, achieved by mapping this manifold onto the product space of translation, rotation, and torsion degrees of freedom. It ranks the docking poses by confidence model. DiffDock has been applied to obtain complexes of acetyl-coenzyme synthetase 2 with inhibitor and showed comparative performance [45], however, there is no published data of applying DiffDock for CYP450s.

Structure-based approaches provide an intuitive understanding of protein-ligand interactions at the atomistic level. However, CYP450s exhibit broad selectivity towards various chemicals due to the diverse shape, size, and different chemical characteristics of residues within the active sites of different isoforms. Additionally, the flexibility of CYP450s structure and their active sites result in complex interactions with water molecules, presenting significant challenges to structure-based methods. While most published molecular docking programs have their own characteristics and can provide relatively accurate results to obtain protein-ligand complexes, accurate sampling of ligand poses can still be limited by induced-fit effects and enzyme conformation changes. Molecular docking is a computationally expensive process, and docking poses are often selected by simplistic scoring functions. This could lead to inaccurate ranking of docking poses and incorrect prediction of binding free energy [46]. Moreover, molecular docking typically outputs the most stable binding mode, and this may not necessarily be the catalytically active pose [28]. Although these limitations can be improved with more advanced algorithms and scoring functions, particularly the machine learning-based methods, these docking tools are still under development and require further validation through extensive applications in the field to ensure accuracy and reliability.

### 2.2 Ligand-based approaches

**Fig. 3:**
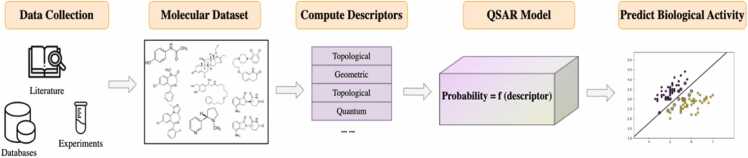
Workflow of QSAR model building. This entails collecting experimental and simulated molecular data from various sources based on the predefined criteria. Descriptors are computed from the processed dataset. A QSAR mathematical model is applied to predict the biological activity of compounds.

Ligand-based approaches have advantages compared to structure-based approaches, particularly in terms of prediction speed, handling with the absence of an experimental 3D structure, addressing flexibility of the CYP450s structure [47]. In the absence of such structural data, insights into ligand molecules binding to enzyme active sites are derived from an analysis of the structural, physicochemical, and biological properties exhibiting correlations with the desired bioactivity [48]. Quantitative structure-activity relationship (QSAR) and pharmacophoric models are the most important techniques in ligand-based approaches. The predominant ligand-based method is the QSAR modeling which is used to establish correlations between molecular descriptors of chemicals and their biological activities through mathematical models, thereby explaining the inherent relationships at a molecular level [20]. (Fig. 3).

In early years, flexible docking was adopted to sample possible binding modes, followed by the utilization of multi-dimensional QSAR models [49], [50] or Boltzmann scoring [51] to successfully predict the binding of small molecules to CYP450s. However, these methods are computationally expensive and encounter challenges when implemented on larger datasets. Nowadays, machine learning algorithms [52] have become the mainstay for building the QSAR models. The related published *models use* algorithms that include classical machine learning model such as support vector machine (SVM), random forests (RF), k-nearest neighbor (kNN), Bayesian approaches; as well as deep learning methods such as convolutional neural network (CNN), graph neural network (GNN), Transformer, and more [20], [53].

**Fig. 4:**
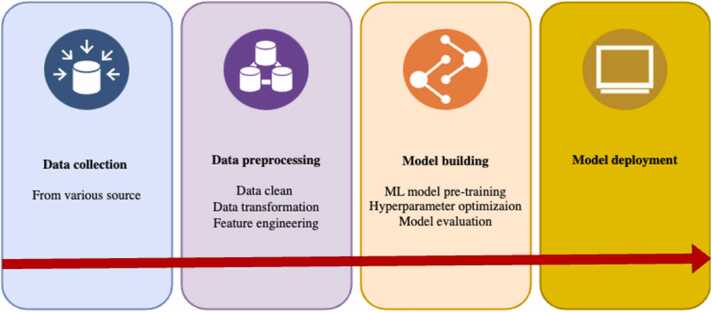
General working flow of machine learning model building. First step is collecting data from various sources. Second step is data preprocessing, which includes data clean, transformation, and feature engineering. Third step is building machine learning model(s), which involves model pre-training, hyperparameter optimization, and model evaluation. Final step is deploying the machine learning mode.

The machine learning building procedure can be briefly summarized into four main stages: raw data gathering, data preprocessing, building machine learning models, and model deployment (Fig. 4). Consistently collecting reliable data to create high-quality datasets is crucial for prediction performance. Several databases have been published in the CYP450s research field, such as SuperCYP [54], Transformer [55], CypComp [56], P450Rdb [57], along with other biochemistry databases like OCHEM [58], PubChem BioAssay [59], DrugBank [60], and ChEMBL [61], among others. Retrieving data from relevant publications also serves as a primary method. During data preprocessing, it is essential to carefully remove redundant, missing values, outliers, and to standardize molecular structure. Enzymes and molecules need to be mathematically represented for machine learning models to learn. Molecular representations, including physicochemical descriptors such as geometrical, thermodynamics, electronic, constitutional, topological descriptors, as well as molecular fingerprints like MACCS keys, Mol2Vec and Morgan fingerprints, encode structure features into binary strings based on the presence or absence of substructural fragments. Feature engineering can help prevent overfitting of classification models and enhance efficiency and accuracy. Methods involve using genetic algorithms to identify the most informative features and PCA to reduce computational complexity. The selection of molecular descriptors significantly influences the prediction model performance; therefore, preparing sufficient descriptors is very important. More details about the molecular descriptors and their calculations can be found in these reviews [23], [62]. Once the model is built and validated, it can be deployed for practical use, ensuring it integrates well with the necessary systems and can handle real-time data if required.

To date, published prediction models incorporate both classical machine learning and deep learning algorithms. Support vector machine (SVM) and random forest (RF) are the most commonly used classical machine learning algorithms in this field. SVM utilizes the structural risk minimization principle to classify data by optimizing the separation of classes in an N-dimensional space [63], while RF employs an ensemble of decision trees to determine the output category based on the mode of individual tree outputs [64]. On the other hand, graph-based neural networks (GNNs) and Transformer attention mechanisms are two principal strategies in deep learning-based prediction models. GNNs regard molecules as graphs where atoms represent nodes and bonds represent edges, updating node and edge representations iteratively to capture structural relationships for classification [65]. Transformer attention mechanisms effectively capture intricate relationships and dependencies within the data by assigning weights to different input data to accurate classification [66]. Notably, consensus models can be used to integrate prediction results from multiple models, enhancing the reliability and performance of the prediction model [67]. Table 1 summarizes machine learning-based prediction models for CYP450s – substrates/inhibitors interactions published in the last years.

**Table 1:** Summary of published machine learning-based models for predicting CYP450s – substrates/inhibitors interactions.

Model	Algorithms	Descriptors	Datasets	CYP450s Prediction	Year	Ref.
WhichCyp	SVM	Molecular signatures	17143 substances from PubChem	1A2, 2C9, 2C19, 2D6, 3A4 (inhibitors)	2013	[68]
CypRules	Rule-based C5.0 algorithm	Molecular descriptors	16561 compounds from PubChem	1A2, 2C9, 2C19, 2D6, 3A4 (inhibitors)	2015	[69]
pkCSM	RF, LR	Molecular descriptors and graph-based signature	30 datasets with 18000 compounds from literature	1A2, 2C9, 2C19, 2D6, 3A4 (inhibitors) 2D6, 3A4 (substrates)	2015	[70]
vNN-ADMET	vNN	Molecular and topological fingerprints	More than 40000 compounds from ChEMBL	1A2, 2C9, 2C19, 2D6, 3A4 (inhibitors)	2017	[71]
SwissADME	SVM	Molecular and physicochemical descriptors	About 47000 compounds from PubChem	1A2, 2C9, 2C19, 2D6, 3A4 (inhibitors)	2017	[72]
CypReact	Learning based model	Physicochemical and structure descriptors	1632 compounds from Human Metabolome Database, KEGG, DrugBank, PubChem, literature	1A2, 2A6, 2B6, 2C8, 2C9, 2C19, 2D6, 2E1, 3A4 (substrates)	2018	[56]
WhichP450	RF	Molecular and Structural descriptors	465 compounds from literature	1A2, 2C8, 2C9, 2C19, 2D6, 2E1, 3A4 (substrates)	2018	[73]
DeepCYP	Multitask autoencoder DNN	Molecular fingerprints	Over 13000 compounds from PubChem	1A2, 2C9, 2C19, 2D6, 3A4 (inhibitors)	2018	[74]
admetSAR 2.0	RF, k-NN, SVM	Molecular fingerprints	Over 96000 molecules from DrugBank, CYP450, literature, etc.	1A2, 2D6, 2C8, 2C9 2C19, 3A4 (inhibitors), 2D6, 2C9, 3A4 (substrates)	2019	[75]
SuperCYPsPred	RF	Molecular fingerprints	17143 substances from PubChem	1A2, 2C9, 2C19, 2D6, 3A4 (inhibitor)	2020	[76]
CYPstrate	RF, SVM	Molecular descriptors	1831 compounds from literature	1A2, 2A6, 2B6, 2C8, 2C9, 2C19, 2D6, 2E1, 3A4 (substrate)	2021	[77]
CYPlebrity	RF	Molecular descriptors	18815 compounds from PubChem, ChEMBL, ADME.	1A2, 2C9, 2C19, 2D6, 3A4 (inhibitors)	2021	[78]
ADMETlab 2.0	multi-task graph attention framework	Physicochemical, ADME properties	0.25 M entries from ChEMBL, PubChem, OCHEM, literature.	1A2, 2C9, 2C19, 2D6, 3A4 (inhibitor, substrate)	2021	[79]
iCYP-MFE	Multitask DNN	Molecular fingerprints	17143 compounds from PubChem	1A2, 2C9, 2C19, 2D6, 3A4 (inhibitor)	2021	[80]
HelixADMET	Multitask GNN	ADMET, physicochemical endpoints	Over 70000 molecules from literature and PubChem	1A2, 2C9, 2C19, 2D6, 3A4 (inhibitor, substrate)	2022	[81]
Interpretable-ADMET	GCNN, GAT	Physicochemical and biological properties	80167 compounds from ChEMBL, PubChem, DrugBank, literature	1A2, 2C9, 2C19, 2D6, 3A4 (inhibitor) 2C9, 2C19, 2D6, 3A4 (substrate)	2022	[82]
ESP	GNN, Esm−1b transformer	Molecular fingerprints	18351 (experimental) and 274030 (inferred) enzyme-substrate pairs from UniProt-GOA	-	2023	[83]
DEEPCYPs	Multi-task FP-GNN	Molecular graph and fingerprints	71456 compounds from PubChem	1A2, 2C9, 2C19, 2D6, 3A4 (inhibitor)	2023	[84]
ADMET-AI	Graph neural network	Physicochemical features	41 datasets from Therapeutics Data Commons	1A2, 2C9, 2C19, 2D6, 3A4 (inhibitor) 2C9, 2D6, 3A4 (substrate)	2023	[85]
ADMETlab 3.0	Multi-task DMPNN framework	Physicochemical, ADME properties	4 M entries from ChEMBL, PubChem, OCHEM, literature.	1A2, 2C9, 2C19, 2D6, 3A4 (inhibitor, substrate)	2024	[86]

## 3 Evaluation of existing machine learning prediction models

### 3.1 Selection of prediction models and testing dataset molecules

Structure-based prediction models are computationally expensive and challenging to apply to large-scale datasets. Additionally, molecular docking and MD simulations, which are structure-based approaches, require specialized knowledge and specific computing resources. As a results, these tools are difficult to use for obtaining rapid results and are less accessible to a broad range of users, such as researchers conducting initial chemical investigations, pharmacotherapy professionals, and others. In contrast, ligand-based models address these drawbacks effectively. Specifically, models developed using machine learning methods are trained on large datasets that better cover the chemical space. Most of these models are also non-commercial and open-access prediction tools. These advantages have significantly advanced *in silico* prediction of CYP450s specificity. However, due to the challenge of achieving high accuracy between the machine learning model predictions and real experimental results, it is crucial to test and compare the performance of these models. Therefore, we selected eleven machine learning-based, non-commercial, open access prediction models published between 2015 and 2024. We then utilized the 100 most prescribed drugs from the top 300 drugs of 2021, according to the ClinCalc DrugStats database (https://clincalc.com/DrugStats/Top300Drugs.aspx), to test their prediction performance with respect to experimental or QM calculated data on drugs. This will help users in selecting the most suitable model for prediction and provide researchers with valuable insights for further model development in this field.

The eleven selected prediction models from Table 1 are: pkCSM, vNN-ADMET, SwissADME, CypReact, admetSAR 2.0, SuperCYPsPred, CYPstrate, CYPlebrity, ADMETlab 2.0, ADMET 3.0, ESP. Among these models, vNN-ADMET, SuperCYPsPred, SwissADME, CYPlebrity are dedicated to inhibitors prediction. CYPstrate, CypReact, and ESP are designed for substrates prediction. pkCSM, admetSAR 2.0, ADMETlab 2.0 and 3.0 can predict both inhibitors and substrates. The datasets for these models were collected from various databases and literature sources, employing different techniques for data preprocessing and handling imbalanced datasets. Molecules were represented using different types of molecular descriptors. The machine learning algorithms used to develop these models varied, including random forest, support vector machine, logistic regression, graph neural networks, transformers, and others. For some models that used the same algorithms, different techniques were applied to improve their performance. For instance, kernelization techniques were used to apply different kernel functions to the support vector machine, enabling it to transform input data into a higher-dimensional space more effectively; architectural enhancement techniques were used to integrate attention mechanisms and convolutional neural networks into graph neural networks, enhancing their ability to learn molecular graphs, and more. This provides us with a comprehensive overview of the effects of different data sources, data preprocessing methods, machine learning algorithms on the performance of prediction models. The details of each model are illustrated below.

**pkCSM** (https://biosig.lab.uq.edu.au/pkcsm/) employs distance-based graph signatures to represent chemical and topological information. It utilizes random forest and logistic regression algorithms to build the classification models, which were trained on datasets containing inhibitor prediction data for CYP1A2, CYP2C9, and CYP2D6 (each dataset includes over 14,000 molecules), as well as substrate prediction datasets for CYP2D6 and CYP3A4 (each dataset contains 671 compounds).

**vNN-ADMET** (https://vnnadmet.bhsai.org/) uses the variable nearest neighbor (vNN) method [87] to construct prediction models for the inhibitors of CYP1A2, CYP2C9, CYP2C19, CYP2D6, and CYP3A4. The vNN method, based on the distance-weighted k-nearest neighbor (kNN), predicts the biological activity of compounds by averaging weights across structurally similar neighbors. Accelrys extended-connectivity fingerprints with a diameter of four chemical bonds were utilized to identify structurally similar compounds. Each isoform dataset contains over 7500 molecules which were collected from ChEMBL. Compounds with IC_50_ values smaller than 10 μM are distinguished as inhibitors, and those greater than 10 μM as non-inhibitors.

The prediction models for CYP450 inhibitors in **SwissADME** (http://www.swissadme.ch/) were developed using SVM with an RBF Gaussian kernel. The datasets for CYP1A2, CYP2C9, CYP2C19, CYP2D6, and CYP3A4 contain 12145, 12727, 8015, 4732, and 10097 compounds, respectively. Molecular data were gathered from literature and PubChem database. Hierarchical grouping and reciprocal nearest neighbor algorithms were implemented to cluster different chemical classes. Each isoform was assigned specific chemical descriptors based on its distinct properties.

**CypReact** (https://bitbucket.org/Leon_Ti/cypreact/src/master/) is a Java software package that predicts substrates of nine CYP450 isoforms (CYP1A2, CYP2A6, CYP2B6, CYP2C8, CYP2C9, CYP2C19, CYP2D6, CYP2E1, and CYP3A4). CypReact was developed via a cost-sensitive learning-based model (LBM). This LBM incorporates five machine learning classifiers, including SVM, logistic regression, decision tree, random forest, and an ensemble method, along with a cost matrix. The cost matrix is a 2 × 2 matrix representing the cost of four different classes (true reactants, true non-reactants, false reactants, and false non-reactants), which helps rebalance the imbalanced datasets. The CypReact model was trained on datasets containing 1632 compounds with different labels and considered 2279 features, including physicochemical descriptors and molecular fingerprints. A five-fold cross-validation was used to select the best classification model for different isoforms.

**admetSAR 2.0** (http://lmmd.ecust.edu.cn/admetsar2), an update from admetSAR [88], provides various tools for evaluating the ADMET properties of chemicals. SVM, RF, and kNN algorithms were utilized to build classification models for predicting inhibitors of CYP1A2, CYP2D6, CYP2C8, CYP2C9, CYP2C19, CYP3A4, as well as substrates of CYP2D6, CYP2C9, CYP3A4. The datasets for predicting inhibitors of each isoform includes more than 14,000 molecules, while the datasets for substrates prediction includes about 670 compounds for each isoform. To enhance model performance with imbalanced datasets, two techniques were used: synthetic minority over-sampling, which creates new samples by interpolating between minority class samples, and random under-sampling, which randomly reduces the number of majority class. Molecular data were represented using six numerical fingerprints.

**SuperCYPsPred** (https://insilico-cyp.charite.de/SuperCYPsPred/) is a user-friendly web server developed to predict inhibitors of CYP1A2, CYP2C9, CYP2C19, CYP2D6, and CYP3A4 using a random forest algorithm. The model datasets consist of 17,143 substances, collected from PubChem AID: 1851, SuperCYP databases and literature. To address dataset imbalance problem, various data sampling methods were used, including RandUS, AugRandomUS, RandOS, AugRandOs, kMedoids, SMOTETC and SMOTETVDM. Molecules were represented using MACCS or Morgan fingerprints, depending on the specific prediction.

**CYPstrate** (https://nerdd.univie.ac.at/cypstrate/) is a module of the New E-Resource for Drug Discovery (NERDD) web portal [89] for predicting substrates of CYP1A2, 2A6, 2B6, 2C8, 2C9, 2C19, 2D6, 2E1, and 3A4. The core dataset consists of 1831 compounds, and was evaluated by PCA-based comparative analysis and a Tanimoto coefficient-based pairwise maximum similarities analysis for the coverage on drugs, cosmetic ingredients, and agrochemicals. The molecular data were represented as four different features: MACCS keys, Mol2vec descriptors, Morgan fingerprints, and RDKit 2D descriptors. SVM with an RBF kernel function and RF algorithms were used to built the prediction models. The final models were created via consensus decision strategy, which used a grid search with five-fold cross-validation optimization to evaluate individual or combination models.

**CYPlebrity** (https://nerdd.univie.ac.at/cyplebrity/) is another module of the NERDD web portal for predicting inhibitors of CYP1A2, CYP2C9, CYP2C19, CYP2D6, CYP3A4. The datasets have 134,844 molecules, and were iteratively collected from PubChem, ChEMBL, ADME databases. Nearest neighbor similarity and t-SNE were employed to evaluate chemical coverage of the dataset, Morgan3 fingerprints and 44 physicochemical molecular descriptors were calculated as features for the models training. The classification models were built using random forest algorithm.

**ADMETlab 2.0** (https://admetmesh.scbdd.com/) is a web platform fully redesigned from the original ADMETlab [90], focusing on predicting molecular pharmacokinetics and toxicity. It can predict both substrates and inhibitors for CYP1A2, CYP2C9, CYP2C19, CYP2D6, and CYP3A4. The prediction models were developed using a multi-task graph attention framework which constitutes of the input, relation graph convolution network (RGCN) layers, attention layer, and fully connected (FC) layers. The RGCN layers take each atom of the input molecules as a node and convert each node to represent the features of each atom in the circular substructure. The attention layers assign different attention weights to the corresponding substructures and generate customized fingerprints for the prediction tasks. The FC layers adopt different loss functions to complete the predictions. The BCEWithLogitsLoss loss function and the positive samples weights were adopted to address the imbalanced datasets. **ADMETlab 3.0** (https://admetlab3.scbdd.com/) is the newest version of ADMETlab. It incorporates additional features and can predict a broader range of molecular properties. Instead of using RGCN and attention layers in ADMETlab 2.0 models, ADMETlab 3.0 utilizes a directed message passing neural network (DMPNN) block to process both atomic and bond embeddings from the input molecular graph. These embeddings are then converted into predicted property values using a feed-forward neural network.

**ESP** (https://esp.cs.hhu.de/) web server was built to predict enzyme-substrate complexes in general. The datasets for ESP were created by retrieving enzymes and molecules data from the Uniprot-GO annotation database, comprising 18,351 enzyme-substrate pairs in the experimental datasets and 274,030 enzyme-substrate pairs in the systematically inferred datasets. All the small molecules are regarded as positive data points, and their pairwise similarity have been calculated to create negative data points, enabling balanced molecular data points distribution in the datasets. The enzymes representations were obtained by a modified ESM-1b Transformer which is a protein language model, and the molecules representations were obtained by a graph neural network. Subsequently, a gradient boosting model was trained on the pairs of enzyme-substrate representations, allowing the ESP model to successfully predict novel enzyme-substrate pairs.

**Fig. 5:**
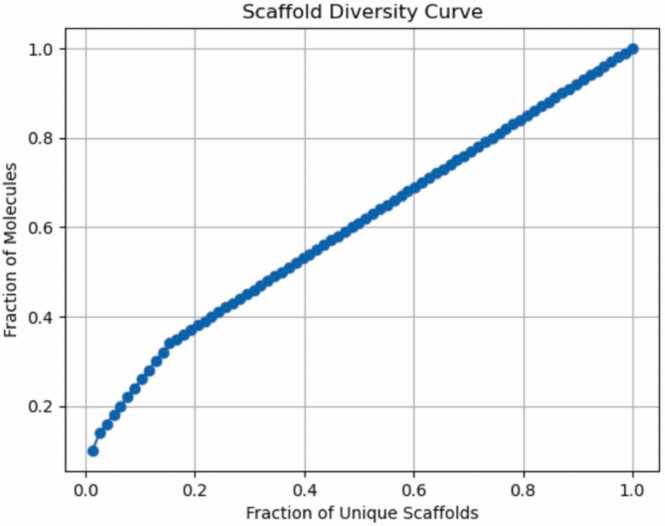
Scaffold diversity curve for our testing dataset. The curve was calculated by RDkit.Chem.Scaffolds package [91] and indicates the fraction of molecules in the dataset that are covered as the number of Murcko scaffolds increases.

As human CYP450s are the major enzymes for drug metabolism, understanding whether a drug molecule is a substrate or inhibitor of a specific CYP450 isoform is crucial for drug discovery and patient treatment planning. Therefore, we utilized the 100 most prescribed drugs to test above eleven prediction models. Even though our dataset contains only 100 drugs, these are the most prescribed drugs and hold significant clinical relevance. They also have more published experimental data to help us verify the performance of prediction models. Additionally, each drug has different structure, and most of them do not share the same privileged structure. The molecular scaffold diversity is shown in the Scaffold Diversity Curve (Fig. 5), the curve has a gradual slope indicating in our dataset the compounds have an even distribution across many scaffolds. This enables us to evaluate the generalizability of prediction models in a non-redundant medicinal chemical space. However, as most of the prediction models only reported their dataset sources and the number of molecules in their datasets, we cannot determine whether our testing dataset drug molecules are included in the testing prediction models datasets. For the ESP model, which published its dataset, our testing data were not included in their training datasets.

### 3.2 Prediction models testing results

The performance of the predictive models has been assessed via different metrics including sensitivity (SN), specificity (SP), accuracy (ACC), Matthew’s correlation coefficient (MCC), and F1 score:

(1) $$\mathrm{SN}=\;\frac{\mathrm{TP}}{\mathrm{TP}+\mathrm{FN}}$$

(2) $$\mathrm{SP}=\;\frac{\mathrm{TN}}{\mathrm{FP}+\mathrm{TN}}\;$$

(3) $$\mathrm{ACC}=\;\frac{\mathrm{TP}+\mathrm{TN}}{\mathrm{TP}+\mathrm{TN}+\mathrm{FP}+\mathrm{FN}}$$

(4) $$\mathrm{MCC}=\;\frac{\mathrm{TP}\times \mathrm{TN}-\mathrm{FP}\times \mathrm{FN}}{\sqrt{\left(\mathrm{TP}+\mathrm{FP})(\mathrm{TP}+\mathrm{FN})(\mathrm{TN}+\mathrm{FP})(\mathrm{TN}+\mathrm{FN}\right)}}$$

(5) $$F1=\;\frac{2\;\times \mathrm{TP}}{2\;\times \mathrm{TP}+\mathrm{FP}+\mathrm{FN}}$$

Where, TP, TN, FP, and FN denote the numbers of true positives, true negatives, false positives, and false negatives, respectively. Among these metrics, SN indicates how well the model correctly identifies the positive cases out of all the actual positive cases. SP shows how well the model correctly identifies the negative cases out of all the actual negative cases. ACC measures the overall correctness of the model. MCC measures the quality of binary classifications and takes the real TP, TN, FP, and FN values into account, making it suitable for dealing with imbalanced datasets. MCC values range from −1 to + 1, where + 1 indicates ideal prediction, 0 indicates random performance, negative values imply poor performance or that the model prediction is worse than random guessing [92]. F1 score ranges from 0 to 1 [93], it can be seen as a harmonic average of the classification model precision and recall (the model ability to identify all the relevant cases within one data set [92]). All the tests were done on the prediction model corresponding web server, CYPreact testing was done by Visual Studio Code 1.90.2 for processing its Java software package. Our testing results are shown in Supplementary Information Table S12 and Table S13.

**pkCSM** prediction results indicate that, for inhibitors prediction, sensitivity ranges from 0.21 (CYP3A4) to 0.71 (CYP1A2); specificity is between 0.74 (CYP1A2) and 0.86 (CYP3A4); accuracy is between 0.74 (CYP1A2 and CYP3A4) and 0.79 (CYP2C9, CYP2C19, CYP2D6); MCC varies from 0.05 (CYP2C9) to 0.34 (CYP2D6); F1 score ranges from 0.16 (CYP2C9) to 0.46 (CYP2D6). For substrates prediction, sensitivity, specificity, accuracy, MCC, and F1 score for CYP2D6 are 0.14, 0.57, 0.93, 0.71, and 0.12, respectively. For CYP3A4, the corresponding values are 0.57, 0.78, 0.65, 0.34, and 0.67. This suggests that pkCSM, under our test conditions, performs well in identifying inhibitors of CYP1A2 and non-substrates of CYP3A4 but exhibits lower performance in identifying inhibitors of CYP2C9 and CYP3A4. The prediction accuracy is moderate, with relatively low MCC and F1 score across most predictions, indicating a mild agreement between predicted and actual values.

**vNN-ADMET** prediction results show a sensitivity of 0.75 for CYP2C19, while sensitivity for other isoforms ranges from 0.14 (CYP1A2) to 0.33 (CYP2D6 and CYP3A4). Specificity falls between 0.82 (CYP2D6) and 0.95 (CYP3A4), suggesting vNN-ADMET has a good ability to predict non-inhibitors over inhibitors. The reason could be imbalance in the number of inhibitors and non-inhibitors in each dataset. Accuracy ranges from 0.74 (CYP2D6) to 0.84 (CYP1A2 and CYP2C19). MCC ranges from 0.02 (CYP2C9) to 0.37 (CYP2C19), and F1 score ranges from 0.13 (CYP2C9) to 0.43 (CYP3A4), suggesting the precision and recall of the model are mild. It is also worth noting that, in total, 195 molecules had no prediction, therefore, the dataset chemical coverage should be reassessed.

For **SwissADME** prediction, the CYP1A2 inhibitors prediction has the lowest sensitivity at 0.29, while sensitivities for other isoforms range from 0.55 (CYP3A4) to 0.82 (CYP2D6). This indicates that under our test conditions, SwissADME could not efficiently predict CYP1A2 inhibitors. The specificity is between 0.63 (CYP2D6) and 0.79 (CYP1A2), and the accuracy is between 0.67 (CYP2D6) and 0.75 (CYP1A2), suggesting that the models moderately identify non-inhibitors and have moderate prediction accuracy. MCC value ranges from 0.04 (CYP1A2) to 0.36 (CYP2C19), and F1 score ranges from 0.14 (CYP1A2) to 0.47 (CYP2C19). Additionally, SwissADME cannot predict molecules using SMILE strings longer than 200 characters and the inorganic salt compounds. This limitation resulted in no prediction of four relative molecules from our dataset.

**CypReact** testing results indicate that no reactant for CYP2A6 was predicted from our dataset, so the sensitivity, MCC, F1 score for CYP2A6 cannot be calculated. Sensitivity for other isoforms ranges from 0.50 (CYP2C8) to 1.00 (CYP2C9 and CYP2E1). Specificity values vary across different isoforms: 0.18 (CYP3A4), 0.45 (CYP2C19), 0.48 (CYP2C9), 0.58 (CYP2D6), 0.64 (CYP1A2), 0.67 (CYP2C8), 0.75 (CYP2B6), 0.90 (CYP2A6), and 0.92 (CYP2E1), showing that CypReact has significant ability to predict non-reactants among different isoforms. Accuracy ranges from 0.49 (CYP2C19) to 0.92 (CYP2E1). MCC is between 0.07 (CYP2C8) and 0.46 (CYP2D6), and F1 score ranges from 0.11 (CYP2C8) to 0.65 (CYP3A4). This indicates that CypReact shows moderate precision and recall on CYP2D6 substrates prediction, while the CYP2C8 prediction has much worse precision and recall than other isoforms.

**admetSAR 2.0** results show the following performance metrics: For inhibitors prediction, sensitivity ranges from 0.22 (CYP2C9) to 0.50 (CYP2C8); specificity ranges from 0.72 (CYP1A2) to 0.82 (CYP2D6); accuracy ranges from 0.71 (CYP1A2) to 0.77 (CYP2C8); MCC ranges from 0.09 (CYP1A2) to 0.20 (CYP3A4). For substrates prediction: sensitivity ranges from 0.68 (CYP2D6) to 0.89 (CYP3A4); speicificity ranges from 0.46 (CYP3A4) to 0.85 (CYP2C9); accuracy ranges from 0.65 (CYP3A4) to 0.85 (CYP2C9); MCC ranges from 0.38 (CYP3A4) to 0.56 (CYP2C9); F1 score ranges fro, 0.38 (CYP3A4) to 0.66 (CYP2D6). These results indicate that admetSAR 2.0 performs well in predicting substrates for CYP2C9 and CYP2D6, and effectively identify non-inhibitors for all predicted isoforms.

**SuperCYPsPred** results show that the MACCS sensitivity ranges from 0.14 (CYP1A2) to 0.65 (CYP2D6), while Morgan sensitivity ranges from 0.11 (CYP2C9) to 0.71 (CYP2D6). MACCS specificity ranges from 0.70 (CYP2C9) to 0.96 (CYP1A2), and Morgan specificity ranges from 0.68 (CYP2D6) to 0.93 (CYP2C19). MACCS accuracy ranges from 0.67 (CYP2C9) to 0.90 (CYP1A2), and Morgan accuracy ranges from 0.69 (CYP2D6) to 0.88 (CYP2C19). MACCS MCC ranges from 0.02 (CYP2C9) to 0.43 (CYP3A4), while Morgan MCC ranges from 0.04 (CYP2C9) to 0.30 (CYP2D6). MACCS F1 score ranges from 0.15 (CYP2C9) to 0.50 (CYP3A4), and Morgan F1 score ranges from 0.12 (CYP2C9) to 0.44 (CYP2D6). Sensitivity and specificity results indicate that both MACCS and Morgan models are effective at predicting non-inhibitors of all the isoforms, however, the ability of correctly identifying inhibitors, under our testing conditions, are relatively low and vary widely across different isoforms. MCC and F1 score results suggest that MACCS models have a slightly better balance between precision and recall.

All the drug molecules were tested by the **CYPstrate** best performance models. The results show the following performance metrics: sensitivity ranges from 0.50 (CYP2C8) to 1.00 (CYP1A2 and CYP2E1); specificity ranges from 0.61 (CYP3A4) to 0.99 (CYP2A6); accuracy ranges from 0.75 (CYP2C8) to 0.99 (CYP2A6); MCC ranges from 0.26 (CYP2B6) to 0.86 (CYPCYP2D6); F1 score ranges from 0.10 (CYP2C8) to 0.90 (CYP2D6). These results point out that CYPstrate has a comparable ability to accurately predict substrates and non-substrates across all tested isoforms. However, the models for CYP2B6 and CYP2C8 show, under our test conditions, less reliable performance compared to other isoforms. It is worth noting that no substrate for CYP2A6 was predicted, so its sensitivity and MCC values cannot be calculated. Additionally, 31, 35, 35, 35, 27, 9, 33, 25, and 6 molecules in the CYP1A2, CYP2C9, CYP2C19, CYP2D6, CYP3A4, CYP2B6, CYP2C8, and CYP2E1 datasets, respectively, had no predictions. This suggests that CYPstrate cannot determine whether these molecules are substrates or non-substrates for the corresponding isoforms. While the core dataset has been evaluated by comparative analysis to show it can cover the major drug space well, its actual datasets scope still limit the prediction effectiveness.

**CYPlebrity** results show that sensitivity ranges from 0.43 (CYP1A2) to 0.67 (CYP2C19); specificity is between 0.82 (CYP1A2 and CYP2C19) and 0.86 (CYP2C9 and CYP3A4); accuracy ranges from 0.77 (CYP3A4) to 0.82 (CYP2C9); MCC ranges from 0.16 (CYP1A2) to 0.43 (CYP2D6); F1 score ranges from 0.23 (CYP1A2) to 0.54 (CYP2D6). Thereby, CYP2D6 model has the best performance with balanced metrics, while CYP1A2 model has moderate ability to identify the true inhibitors. Overall high specificity indicates a strong ability to correctly determine non-inhibitors for each isoform. However, the models generally seem less efficient in predicting true inhibitors.

**ADMETlab 2.0** results show that the inhibitors prediction sensitivity ranges from 0.44 (CYP2C9) to 0.67 (CYP2C19), specificity ranges from 0.71 (CYP2D6) to 0.82 (CYP1A2 and CYP2C9), accuracy ranges from 0.68 (CYP3A4) to 0.80 (CYP1A2), MCC ranges from 0.19 (CYP2C9 and CYP3A4) to 0.29 (CYP2C19), F1 score ranges from 0.28 (CYP2C9) to 0.42 (CYP2D6). For substrate prediction, sensitivity is between 0.90 (CYP2C9) and 1.00 (CYP1A2 and CYP2C19), specificity ranges from 0.40 (CYP2C19) to 0.68 (CYP2D6), accuracy is between 0.51 (CYP2C19) and 0.76 (CYP2D6), MCC ranges from 0.32 (CYP2C19) to 0.58 (CYP2D6), F1 score ranges from 0.41 (CYP2C19) to 0.76 (CYP3A4). ADMETlab 2.0 performs strongly in correctly identifying non-substrates/inhibitors across different isoforms, and the overall accuracy is relatively high. However, the sensitivity, MCC, and F1 score are relatively low, indicating weakness in identifying actual substrates/inhibitors, and low model robustness.

**ADMETlab 3.0** results show that the inhibitors prediction sensitivity ranges from 0.29 (CYP2D6) to 0.78 (CYP2C19), specificity ranges from 0.72 (CYP2C8) to 0.87 (CYP2C19), accuracy ranges from 0.71 (CYP2D6 and CYP2C8) to 0.86 (CYP2C19), MCC ranges from 0.08 (CYP2D6) to 0.47 (CYP2C19), F1 score ranges from 0.12 (CYP2C8) to 0.5 (CYP2C19). For substrates prediction, sensitivity with the lowest 0.09 (CYP2B6), others are between 0.59 (CYP2D6) and 0.95 (CYP3A4), specificity ranges from 0.41 (CYP2C19) to 1.00 (CYP2B6), accuracy ranges from 0.48 (CYP2C19) to 0.80 (CYP2D6 and CYP2B6), F1 scores are between 0.17 (CYP2B6) and CYP3A4 (0.73). We can see ADMET lab 3.0 can predict substrates and inhibitors for more CYP450 isoforms. However, under our test conditions, there is no significant improvement in the prediction performance of the model, with some performance metrics even slightly worse than ADMET lab 2.0.

**ESP** results show that the specificity is around 0.96, and the accuracy ranges from 0.56 (CYP3A4) to 0.96 (CYP2A6). However, the sensitivity, MCC, and F1 score are around 0. These results indicate that the model prediction for CYP450s substrates is close to random guessing. The authors stated that the model high prediction performance is limited to the enzymes and molecules in their datasets, and our tested molecules are not included in their datasets.

**Fig. 6:**
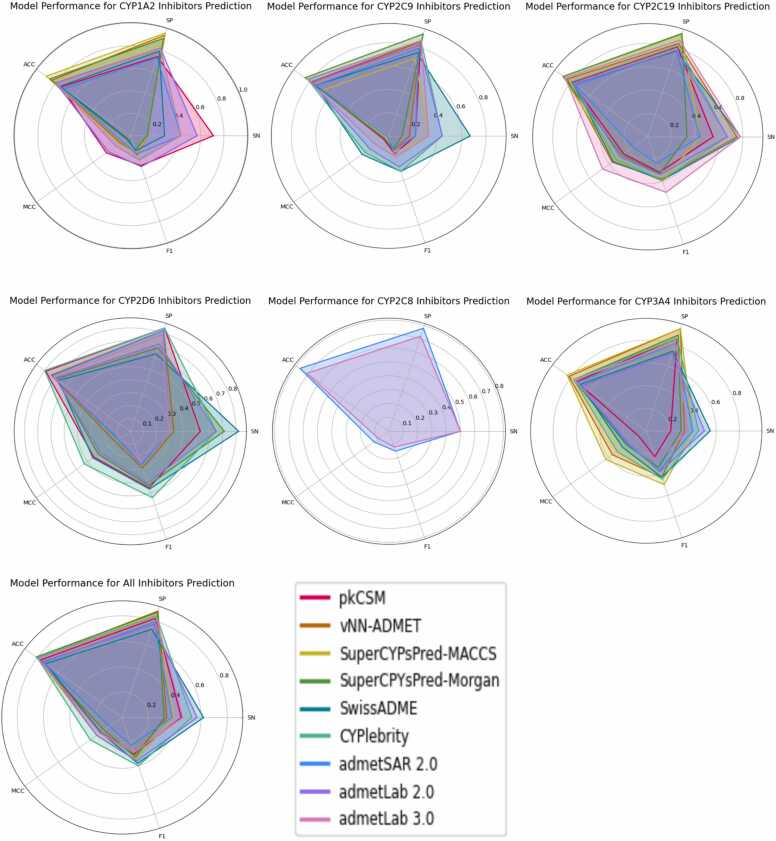
Radar charts of model performance for CYP450s inhibitors prediction. Plots with different colors represent the performance of each prediction model. The closer a plot is to the edge of the radar plot, the better the performance metrics. When a prediction model is unable to predict inhibitors for some isoforms, their performance metrics are not shown in the corresponding CYP450 isoform’s radar plot.

In Fig. 6, the radar charts display the results of inhibitor predictions under our test conditions. pkCSM exhibits the highest MCC and F1 score in predicting CYP1A2 inhibitors. Both vNN-ADMET and SwissADME demonstrate similarly strong predictive performance across all models except predicting CYP2D6 inhibitors. However, vNN-ADMET generated no predictions for approximately 40 % of drugs, indicating its prediction coverage is limited. CYPlebrity achieves the highest overall MCC and F1 score, suggesting the model prediction reliability. admetSAR 2.0 and admetLab 3.0 can predict more isoforms inhibitors than other models. admetSAR 2.0 is better at predicting CYP2C8 and CYP3A4 inhibitors, while ADMETlab 3.0 shows better prediction performance on other isoforms. Comparing ADMETlab 2.0 and 3.0, the newest version shows the best MCC and F1 score in predicting CYP2C19 inhibitors, but except for this aspect, its overall performance is lower than the ADMETlab 2.0 one. Nonetheless, these two models exhibit above-average performance across other metrics.

**Fig. 7:**
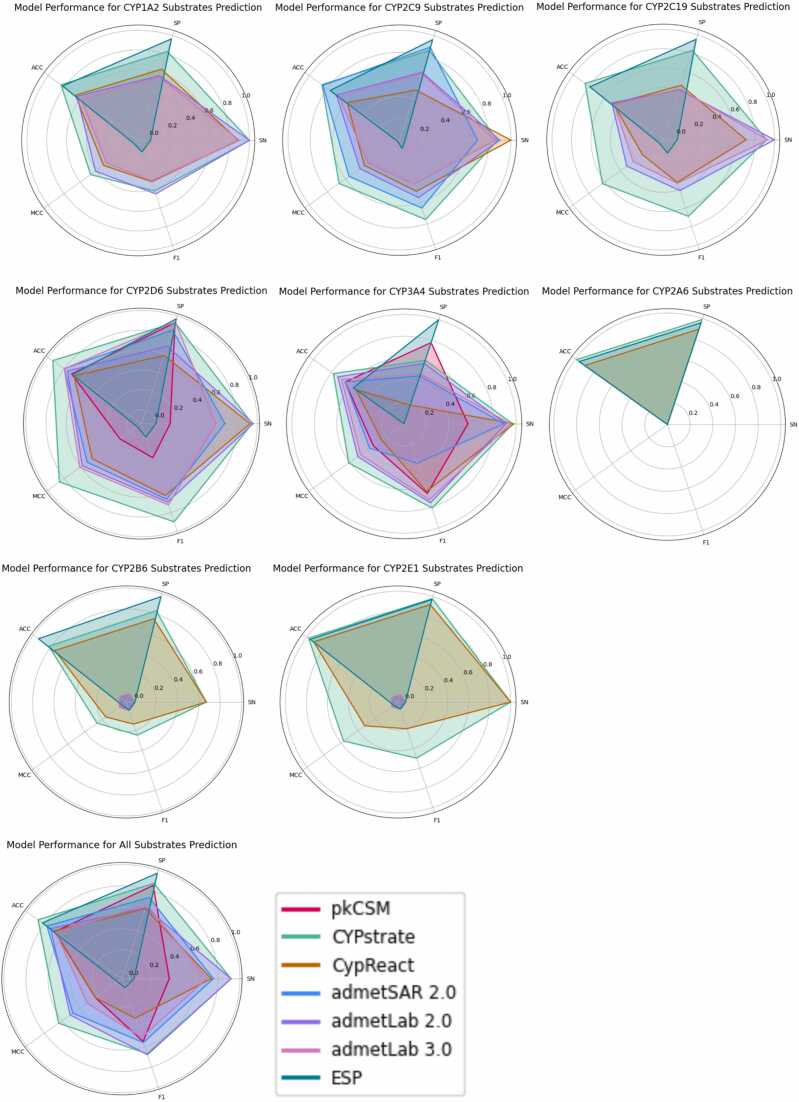
Radar charts of model performance for CYP450s substrates prediction. Plots with different colors represent the performance of each prediction model. The closer a plot is to the edge of the radar plot, the better the performance metrics. When a prediction model is unable to predict substrates for some isoforms, their performance metrics are not shown in the corresponding CYP450 isoform’s radar plot.

The radar charts, presenting the results of substrates prediction, are depicted in Fig. 7. pkCSM shows the good capability to predict both substrates and non-substrates for CYP2D6 and CYP3A4. CYPstrate achieves superior MCC and F1 scores overall, indicating the robustness of the model. However, it did not predict about the 25 % of tested drugs, revealing a limitation in chemical space coverage. CypReact, while slightly underperforming compared to CYPstrate, provides predictions for all drugs. admetSAR 2.0, ADMETlab 2.0 and 3.0 show above-average predictive performance among the models, but ADMETlab 3.0 performance metrics are also slightly worse than ADMETlab 2.0. ESP shows excellent specificity and accuracy. However, its MCC and F1 scores are notably low, some even negative, suggesting, under our test conditions, ESP predictions close to a random guessing.

Comparing classical machine learning-based prediction models (pkCSM, vNN-ADMET, SwissADME, CypReact, SuperCYPsPred, CYPlebrity, CYPstrate, admetSAR 2.0) to deep learning-based prediction models (ADMETlab 2.0 and 3.0, ESP), we can observe distinct advantages and drawbacks for each approach. Classical machine learning based models often work with smaller datasets, limiting their ability to predict properties for some molecules. Despite this limitation, they tend to exhibit higher reliability, as reflected in their better MCC and F1 scores compared to deep learning models. On the other hand, deep learning models, which were trained on larger datasets, generally achieve higher prediction accuracy. However, their MCC and F1 scores are usually lower than those of classical machine learning models, indicating that their prediction results might not be reliable. Importantly, under our test conditions, the performance metrics of all models were lower than the original published values, this evidence is also supported by a previous review [24]. Moreover, some prediction models did not consider the molecular binding affinity to the enzymes, but only produced a “yes” or “no” result, which would be problematic when dealing with molecules acting as weak substrates or inhibitors. Therefore, improving *in silico* prediction models to match the accuracy of experimental results remains a substantial challenge.

## 4 Future trends in *in silico* predictive models for CYP450s specificity prediction development

*In silico* prediction of CYP450s specificity is crucial for investigating the role of CYP450s in xenobiotic metabolism. This can significantly improve the pre-selection procedure of lead compounds for drug discovery, drug-drug interaction for patient treatment planning, and chemical toxicological studies. Both structure-based and ligand-based approaches are important and have their own characteristics in this field of study.

Structure-based approaches offer highly accurate predictions by directly investigating enzyme-molecule interactions at an atomistic level. However, their computational intensity limits their applications to larger molecular datasets and makes them less accessible to individuals without the relevant molecular modeling knowledge and computing resources. Therefore, these approaches are better suited for focusing on specific enzyme-substrate interactions or validating results obtained through ligand-based approaches. Molecular docking is the primary methodology in structure-based approaches, aiming to identify the most stable ligand binding poses. However, the most stable binding poses are not always the reactive ones. Therefore, using quantum mechanics calculation, such as free energy perturbation, can help verify the most accurate binding mode. Additionally, insufficient docking algorithms and simplistic empirical scoring function can affect the accuracy of docking results. Researchers can compare different molecular docking programs to study the interaction between CYP450s and chemicals. Meanwhile, machine learning techniques can be employed to develop more advanced molecular docking software. Unlike traditional molecular docking software, which relies on molecular physicochemical representations and limited datasets for training, machine learning methods can convert molecular structural information into numerical vectors. These vectors are easier to compute and can reveal hidden molecular chemical and structural features. Also, advanced algorithms used in machine learning can improve classification and scoring, then increasing the accuracy of docking results. Nevertheless, many machine learning-based docking programs are either new or still under development. Thus, improving the performance of these docking programs and employing CYP450s data to verify their efficacy in studying CYP450s specificity need many future efforts.

Ligand-based approaches, primarily QSAR models, study the relationship between molecular structure and function via mathematical models. Many ligand-based tools have been developed as web server or software package, and can be free accessed online. This accessibility allows for more efficient ligand-based prediction models and these tools are widely used for predicting large scales of CYP450s substrates and inhibitors. Machine learning techniques can efficiently process large-scale datasets, automatically extract molecular and protein features, and capture the non-linear relationships between molecular descriptors and biological activities. This has made machine learning play an important role in building the ligand-based prediction models. Collecting molecular and enzyme data from multiple sources to create raw datasets is crucial. Data augmentation techniques such as noise injection, data interpolation, and machine learning surrogate model [94] can increase quantity and diversity of training sets, thereby improving the chemical coverage of datasets and the performance and robustness of machine learning models. Most machine learning prediction models only considered physicochemical and topological descriptors, and molecular fingerprints, which are not sufficient for accurate enzyme prediction. Therefore, integrating molecular quantum-chemical descriptors into the input features can help improve prediction accuracy [95]. In addition, with the development of more beyond-rule-of-five drugs [96], it is essential to note that most published prediction models only deal with small molecules, which may not be adequate for contemporary drug discovery pipelines. Moreover, overlooking CYP450s pharmacogenetics and molecular chirality (only considering canonical SMILES strings) in model descriptors also requires improvement.

Nowadays, the size and complexity of datasets in biochemical field is rapidly growing. Deep learning, a type of machine learning that can identify complex patterns in big data and make accurate predictions based on them [97], has become the new trend for building CYP450s specificity prediction models. As the 3D structure of a protein or molecule is directly related to its function, GNNs treat proteins or molecules as graphs, embedding high-dimensional graph structure data into low-dimensional vector spaces. This allows researchers to study the enzyme-molecule interactions easily and accurately in space. PLMs treat protein sequences as human language, representing each amino acid as a character, and employ deep neural networks, such as Transformers, to learn statistical patterns within this “language”. While both GNNs and PLMs have achieved significant success in biological studies, their robustness and performance in predicting CYP450s specificity still need improvement. How to fine-tune these models to better fit CYP450 data is important for further research progress.

## 5 Conclusion

Accurate prediction of CYP450 specificity holds significant importance in drug discovery, chemical toxicology, and patient treatment planning. Computational approaches offer a means to expedite predictions, substantially reduce experimental costs and minimize environmental pollution. This review provides an overview of past 20 years of *in silico* studies on CYP450s specificity, categorized into structure-based and ligand-based approaches.

Structure-based approaches mainly use molecular docking, MD simulations, and QM calculations to explicitly study the enzyme-chemicals complexes at an atomistic level. These methods are highly accurate but computationally expensive. Additionally, they require users to have professional knowledge and access to specific computing resources. This limits the application of these approaches to large-scale datasets and a broad group of users. They are more commonly utilized in studying the protein-ligand interactions for specific molecule(s) with specific CYP450 isoform(s). QSAR modeling is the predominant ligand-based approach. It employs mathematical models to build correlations between molecular descriptors of chemicals and their biological activities, thereby explaining the inherent relationships at a molecular level. Nowadays, machine learning techniques are the mainstay for building prediction models in ligand-based approaches. More and more machine learning-based prediction models have been developed as open-access web servers or software packages. People can easily use those prediction tools to have initial investigations on molecules of interest.

We then used 100 of the most prescribed drugs to assess 11 published prediction models. These prediction models were developed using various kind of classical machine learning or deep learning methods, and were published between 2015 and 2024. Our results indicate that both classical machine learning methods and deep learning methods can achieve a certain prediction accuracy, however, they are still not highly consistent with the experimental or the QM-calculated data. Among the 11 models, CYPlebrity showed the best overall MCC and F1 score for inhibitors prediction, while pkCSM demonstrated the best MCC and F1 score for CYP1A2. Additionally, admetSAR 2.0 and admetLab 3.0 were capable of predicting the largest number of CYP450 isoforms. For substrates prediction, CYPstrate achieved superior MCC and F1 scores overall, indicating robustness, but failed to predict about 25 % of the tested drugs, revealing a limitation in the chemical space coverage. Conversely, CypReact slightly underperformed compared to CYPstrate but provided predictions for all drugs. The quality and scope of datasets, as well as chemical descriptors, are critical for the performance of prediction models. Moreover, machine learning, particularly deep learning methods, are the important future research trend for both structure-based and ligand-based approaches. However, these methods are newly developed, and model robustness and performance still need to be improved and further validated by real experimental data.

We think this review provides readers with a comprehensive overview of *in silico* CYP450s specificity prediction studies. It aims to assist users, such as researchers conducting initial investigations into molecules of interest, as well as pharmacotherapy professionals to predict DDIs and create better therapeutic schemes by choosing suitable models for prediction. Additionally, it gives suggestions for researchers to do better development of prediction models in this field.

## Funding

Yao Wei, Luca Palazzolo, Uliano Guerrini, and Ivano Eberini were financially supported by the European Union’s Horizon Europe research and innovation program under the Marie Skłodowska-Curie grant agreement No. 101073546 (MSCA Doctoral Network Metal-containing Radical Enzymes – MetRaZymes). All the authors were supported by grants from MIUR - “Progetto Eccellenza 2023 – 2027". Luca Palazzolo was supported by PSR2022 – Azione A from Department of Pharmacological and Biomolecular Sciences, 10.13039/100012352Università degli Studi di Milano.

## CRediT authorship contribution statement

**Tommaso Laurenzi:** Writing – review & editing. **Davide Bianchi:** Writing – review & editing. **Ivano Eberini:** Writing – review & editing, Validation, Supervision, Resources, Project administration, Funding acquisition, Conceptualization. **Uliano Guerrini:** Writing – review & editing, Software. **Yao Wei:** Writing – review & editing, Writing – original draft, Visualization, Validation, Software, Methodology, Investigation, Formal analysis, Data curation, Conceptualization. **Omar Ben Mariem:** Writing – review & editing, Software. **Luca Palazzolo:** Writing – review & editing, Validation, Funding acquisition.

## Declaration of Competing Interest

The authors declare that they have no known competing financial interests or personal relationships that could have appeared to influence the work reported in this paper.
