# Supplementary material for: Investigation of in silico studies for cytochrome P450 isoforms specificity
Source: Comput Struct Biotechnol J. 2024 Aug 5;23:3090–103. doi: 10.1016/j.csbj.2024.08.002 (PMC11347072; doi:10.1016/j.csbj.2024.08.002)
Supplement: Supplementary file 1 — Supplementary material. [file mmc1.docx]

In Silico Prediction Tools for Cytochrome P450 Isoforms Specificity: A Comprehensive Review and Model Evaluation

**Supplementary Information**

Yao Wei, Luca Palazzolo, Omar Ben Mariem, Davide Bianchi, Tommaso Laurenzi, Uliano Guerrini, Ivano Eberini^*^

Dipartimento di Scienze Farmacologiche e Biomolecolari “Rodolfo Paoletti”, Università degli Studi di Milano, Via Giuseppe Balzaretti 9, 20133, Milano, Italy

^*^Corresponding author email: ivano.eberini@unimi.it

**Table S 1. Information on substrates, inhibitors, and inducers of drugs for different human CYP450 isoforms**

| **Drugs** | **1A2** | **2A6** | **2B6** | **2C8** | **2C9** | **2C19** | **2D6** | **2E1** | **3A4** |
| --- | --- | --- | --- | --- | --- | --- | --- | --- | --- |
| Atorvastatin |  | id [4] | id [4] | ih [2] | ih [3] | ih [3] | ih [3] |  | ss [1] |
|  |  |  |  |  |  |  |  |  | Ih [37] |
|  |  |  |  |  |  |  |  |  | id[56] |
| Levothyroxine |  |  | Ih [34] |  |  |  |  |  | ih[55] |
| Metformin |  |  |  |  |  |  |  |  |  |
| Lisinopril |  |  |  |  |  |  |  |  |  |
| Amlodipine |  |  | Ih [34] |  |  |  | ih[38] |  | ss [5] |
| Metoprolol |  |  |  |  |  |  | ss [5] |  | ih[5] |
|  |  |  |  |  |  |  | ih[58] |  |  |
| Albuterol |  |  |  |  |  |  | ih[38] |  | ih[39] |
|  |  |  |  |  |  |  |  |  | ss[66] |
| Omeprazole | id[8] | ih[41] |  |  |  | ss[5] | ih[41] |  | ss[5] |
|  |  |  |  |  |  | ih[6] | ss[61] |  | ih[7] |
|  |  |  |  |  |  |  |  |  | id[61] |
| Losartan |  |  |  | ss[61] | ss[5] | ih[42] |  |  | ss[5] |
| Gabapentin |  | ih[67] |  |  |  |  |  |  |  |
| Hydrochlorothiazide |  |  |  |  |  |  |  |  |  |
| Sertraline |  |  | ih[34] |  | ss[43] | ss[45] | ss[45] |  | ss[5] |
|  |  |  |  |  | ih[44] | ih[45] | ih[5] |  |  |
| Simvastatin |  |  | ih[34] |  | ih[3] |  | ss[46] |  | ss[5] |
|  |  |  |  |  |  |  |  |  | ih[61] |
| Montelukast |  |  | ih[34] | ih[10] | ss[47] |  |  |  | ss[5] |
| Escitalopram |  |  |  |  |  | ss[11] | ih[12] |  | ss[5] |
|  |  |  |  |  |  |  | ss[86] |  |  |
| Acetaminophen | ss[20] |  |  |  |  |  | ss[48] | ss[5] | ss[48] |
|  |  |  |  |  |  |  |  |  | ih[49] |
| Hydrocodone |  |  |  |  |  | ss[50] | ss[51] |  | ss[52] |
| Rosuvastatin |  |  |  |  |  |  |  |  | ss[5] |
| Bupropion | ss[85] |  | ss[13] |  |  | ss[60] | ih[5] |  | ss[85] |
| Furosemide |  |  |  |  |  |  |  |  |  |
| Pantoprazole |  |  |  |  | ih[15] | ss[14] |  |  | ih[15] |
| Trazodone |  |  |  |  |  | ss[56] | ss[56] |  | ss[16] |
| Dextroamphetamine |  |  |  |  |  |  | ss[57] |  |  |
| Dextroamphetamine Saccharate |  |  |  |  |  |  |  |  |  |
| Amphetamine Aspartate |  |  |  |  |  |  |  |  |  |
| Fluticasone |  |  |  |  |  |  |  |  | ss[68] |
| Tamsulosin |  |  |  |  |  |  | ss[53] |  | ss[53] |
| Fluoxetine | ih[20] |  |  | ss[61] | ih[5] | ih[19] | ih[5] |  | ih[61] |
|  |  |  |  |  | ss[17] | ss[84] | ss[18] |  |  |
| Carvedilol | ss[69] |  | ih[34] |  | ss[5] | ss[69] | ss[5] |  | ih[5] |
| Duloxetine | ss[20] |  |  |  |  | ih[70] | ih[5] |  | ih[70] |
|  | ih[70] |  |  |  |  |  | ss[56] |  |  |
| Meloxicam |  |  |  |  | ss[71] |  |  |  | ss[71] |
| Clopidogrel | ss[20] |  | ih[33] | ih[36] | ih[33] | ih[33] |  |  | ss[5] |
|  |  |  | ss[72] | ss[72] | ss[83] | ss[83] |  |  |  |
| Prednisone |  |  |  |  |  | id[82] |  |  | ss[61] |
|  |  |  |  |  |  |  |  |  | ih[61] |
|  |  |  |  |  |  |  |  |  | id[61] |
| Citalopram | ih[20] |  |  |  |  | ss[56] | ss[61] |  | ss[5] |
|  |  |  |  |  |  |  | ih[61] |  |  |
| Insulin Glargine | id[20] |  |  |  |  |  |  |  |  |
| Potassium Chloride |  |  |  |  |  |  |  |  |  |
| Pravastatin |  |  |  |  |  |  | id[61] |  | ss[5] |
|  |  |  |  |  |  |  |  |  | ih[61] |
| Tramadol |  |  |  |  |  |  | ss[5] |  | ih[5] |
| Aspirin |  |  |  |  | ss[73] | id[74] |  |  |  |
| Alprazolam |  |  |  |  | ss[47] |  |  |  | ss[5] |
| Ibuprofen |  |  |  | ss[61] | ss[5] | ss[54] |  |  | ss[54] |
| Cyclobenzaprine | ss[20] |  |  |  |  |  |  |  |  |
| Amoxicillin |  |  |  |  |  |  |  |  |  |
| Methylphenidate |  |  |  |  |  |  |  |  |  |
| Allopurinol | ih[63] |  |  |  |  |  |  |  |  |
| Venlafaxine |  |  |  |  | ss[76] | ss[76] | ss[56] |  | ss[76] |
|  |  |  |  |  |  |  | ih[56] |  |  |
| Clonazepam |  |  |  |  |  |  |  |  | ss[61] |
| Ethinyl Estradiol | ih[20] |  |  |  | ss[78] | ih[79] |  |  | ss[61] |
|  | ss[77] |  |  |  |  |  |  |  |  |
| Ergocalciferol |  |  |  |  |  |  |  |  |  |
| Zolpidem | ss[61] |  |  |  |  |  | ss[61] |  | ss[5] |
| Apixaban |  |  |  |  |  |  |  |  |  |
| Glipizide |  |  |  |  | ss[5] |  |  |  | ss[56] |
| Spironolactone |  |  |  |  |  |  |  |  |  |
| Cetirizine |  |  |  |  |  |  |  |  |  |
| Atenolol |  |  |  |  |  |  | ss[75] |  |  |
| Oxycodone |  |  |  |  |  |  | ss[5] |  | ss[80] |
| Buspirone |  |  |  |  |  |  |  |  | ss[5] |
| Salmeterol |  |  |  |  |  |  |  |  | ss[56] |
| Topiramate |  |  |  |  |  | ih[56] |  |  | id[56] |
| Warfarin | ss[20] |  |  | ih[61] | ss[17] | ss[61] | ss[61] |  | ss[5] |
|  |  |  |  |  |  |  |  |  | id[56] |
| Estradiol | ss[20] |  |  |  |  |  |  |  | ss[5] |
| Cholecalciferol |  |  |  |  |  |  |  |  |  |
| Budesonide |  |  |  |  |  |  |  |  | ss[61] |
| Formoterol |  |  |  |  |  |  |  |  |  |
| Lamotrigine |  |  |  |  |  |  |  |  |  |
| Norgestimate |  |  |  |  |  |  |  |  |  |
| Quetlapine |  |  |  |  |  |  | ss[88] |  | ss[88] |
| Lorazepam |  |  |  |  |  |  |  |  | ss[87] |

| Famotidine |  |  |  |  |  |  |  |  |  |
| --- | --- | --- | --- | --- | --- | --- | --- | --- | --- |
| Folic Acid |  |  |  |  |  |  |  |  |  |
| Azithromycin |  |  |  |  |  |  |  |  | ss[64] |
|  |  |  |  |  |  |  |  |  | ih[81] |
| Hydroxyzine |  |  |  |  |  |  | ih[65] |  |  |
| Insulin Lispro | id[20] |  |  |  |  |  |  |  |  |
| Diclofenac |  |  |  |  | ss[17] |  |  |  |  |
| Loratadine |  |  | ih[34] |  |  | ih[35] | ss[61] |  | ih[40] |
| Sitagliptin |  |  |  |  |  |  |  |  |  |
| Clonidine |  |  |  |  |  |  |  |  |  |
| Diltiazem | ih[20] |  |  |  |  |  | ss[5] |  | ss[5] |
|  |  |  |  |  |  |  |  |  | ih[37] |
| Latanoprost |  |  |  |  |  |  |  |  |  |
| Pregabalin |  |  |  |  |  |  |  |  |  |
| Doxycycline |  |  |  |  |  |  |  |  |  |
| Insulin Aspart | id[20] |  |  |  |  |  |  |  |  |
| Amitriptyline | ss[20] |  |  |  | ss[17] | ss[21] | ss[5] | ih[62] | ih[5] |
|  |  |  |  |  |  |  | ih[61] |  |  |
| Paroxetine | ih[20] |  | ih[34] |  |  |  | ih[5] |  | ih[5] |
|  |  |  |  |  |  |  | ss[22] |  |  |
| Ondansetron | ss[20] |  |  |  | ss[61] |  | ss[5] |  | ss[23] |
| Tizanidine | ss[24] |  |  |  |  |  |  |  |  |
| Lisdexamfetamine |  |  |  |  |  |  |  |  |  |
| Rivaroxaban |  |  |  |  |  |  |  |  | ss[25] |
| Glimepiride |  |  |  |  | ss[26] |  |  |  |  |
| Propranolol | ss[20] |  |  |  |  | ss[27] | ss[5] |  | ss[28] |
| Aripiprazole |  |  |  |  |  |  | ss[29] |  | ss[29] |
|  |  |  |  |  |  |  | ih[29] |  | ih[29] |
| Finasteride |  |  |  |  |  |  |  |  | ss[30] |
| Naproxen | ss[20] |  |  |  | ss[17] |  |  |  |  |
| Levetiracetam |  |  |  |  |  |  |  |  |  |
| Alendronate |  |  |  |  |  |  |  |  |  |
| Fenofibrate |  |  | ih[34] |  | ih[31] |  |  |  |  |
| Oxybutynin |  |  |  |  |  |  |  |  |  |
| Celecoxib |  |  |  |  | ss[5] |  | ih[32] |  |  |
|  |  |  |  |  | ih[56] |  |  |  |  |
| Lovastatin |  |  |  |  | ih[61] |  |  |  | ss[5] |
| Ezetimibe |  |  | ih[34] |  |  |  |  |  |  |

ss (green): substrate; ih (orange): inhibitor (blue); id: inducer

**Table S 2. pkCSM Testing Results** (Inhibitors and Substrates)

| **Drugs** | **1A2 - ihn** | **2C9 - ihn** | **2C19 -ihn** | **2D6 -ihn** | **3A4 -ihn** | **2D6 -sub** | **3A4 -sub** |
| --- | --- | --- | --- | --- | --- | --- | --- |
| Atorvastatin | n-i | i | n-i | n-i | n-i | n-s | s |
| Levothyroxine | n-i | n-i | n-i | n-i | n-i | n-s | n-s |
| Metformin | n-i | n-i | n-i | n-i | n-i | n-s | n-s |
| Lisinopril | n-i | n-i | n-i | n-i | n-i | n-s | n-s |
| Amlodipine | n-i | n-i | n-i | n-i | n-i | n-s | s |
| Metoprolol | i | n-i | n-i | n-i | n-i | n-s | n-s |
| Albuterol | n-i | n-i | n-i | n-i | n-i | n-s | n-s |
| Omeprazole | i | n-i | i | n-i | n-i | n-s | n-s |
| Losartan | i | i | i | n-i | i | s | s |
| Gabapentin | n-i | n-i | n-i | n-i | n-i | n-s | n-s |
| Hydrochlorothiazide | n-i | n-i | n-i | n-i | n-i | n-s | n-s |
| Sertraline | i | n-i | n-i | i | n-i | n-s | s |
| Simvastatin | n-i | n-i | n-i | n-i | i | n-s | s |
| Montelukast | n-i | i | i | n-i | n-i | n-s | s |
| Escitalopram | i | n-i | n-i | i | n-i | n-s | s |
| Acetaminophen | n-i | n-i | n-i | n-i | n-i | n-s | n-s |
| Hydrocodone | n-i | n-i | n-i | n-i | n-i | n-s | s |
| Rosuvastatin | n-i | n-i | n-i | n-i | n-i | n-s | s |
| Bupropion | i | n-i | n-i | i | n-i | n-s | n-s |
| Furosemide | n-i | n-i | n-i | n-i | n-i | n-s | n-s |
| Pantoprazole | i | n-i | n-i | n-i | n-i | n-s | n-s |
| Trazodone | i | n-i | n-i | n-i | n-i | s | s |
| Dextroamphetamine | i | n-i | n-i | n-i | n-i | n-s | n-s |
| Dextroamphetamine Saccharate | n-i | n-i | n-i | n-i | n-i | n-s | n-s |
| Amphetamine Aspartate | n-i | n-i | n-i | n-i | n-i | n-s | n-s |
| Fluticasone | n-i | n-i | n-i | n-i | n-i | n-s | s |
| Tamsulosin | n-i | n-i | n-i | i | i | n-s | s |
| Fluoxetine | i | n-i | i | i | n-i | n-s | s |
| Carvedilol | i | i | i | i | i | s | s |
| Duloxetine | i | n-i | i | i | n-i | n-s | s |
| Meloxicam | n-i | n-i | n-i | n-i | n-i | n-s | n-s |
| Clopidogrel | i | n-i | n-i | i | n-i | n-s | s |
| Prednisone | n-i | n-i | n-i | n-i | n-i | n-s | s |
| Citalopram | i | n-i | n-i | i | n-i | n-s | s |
| Insulin Glargine | n-i | n-i | n-i | n-i | n-i | n-s | s |
| Potassium Chloride | n-i | n-i | n-i | n-i | n-i | n-s | n-s |
| Pravastatin | n-i | n-i | n-i | n-i | n-i | n-s | n-s |
| Tramadol | n-i | n-i | n-i | i | n-i | n-s | s |
| Aspirin | n-i | n-i | n-i | n-i | n-i | n-s | n-s |
| Alprazolam | i | i | i | n-i | n-i | n-s | s |
| Ibuprofen | n-i | n-i | n-i | n-i | n-i | n-s | n-s |
| Cyclobenzaprine | n-i | n-i | n-i | i | n-i | n-s | s |
| Amoxicillin | n-i | n-i | n-i | n-i | n-i | n-s | n-s |
| Methylphenidate | n-i | n-i | n-i | i | n-i | n-s | n-s |
| Allopurinol | n-i | n-i | n-i | n-i | n-i | n-s | n-s |
| Venlafaxine | n-i | n-i | n-i | n-i | n-i | s | s |
| Clonazepam | i | i | i | n-i | n-i | n-s | s |
| Ethinyl Estradiol | i | n-i | i | n-i | n-i | n-s | s |
| Ergocalciferol | n-i | n-i | n-i | n-i | n-i | n-s | s |
| Zolpidem | i | n-i | i | n-i | n-i | n-s | s |
| Apixaban | n-i | i | i | n-i | i | n-s | s |
| Glipizide | n-i | i | n-i | n-i | n-i | n-s | n-s |
| Spironolactone | n-i | n-i | n-i | n-i | n-i | n-s | s |
| Cetirizine | n-i | n-i | n-i | n-i | n-i | n-s | s |
| Atenolol | n-i | n-i | n-i | n-i | n-i | n-s | n-s |
| Oxycodone | n-i | n-i | n-i | n-i | n-i | n-s | s |
| Buspirone | n-i | i | n-i | i | n-i | n-s | s |
| Salmeterol | n-i | n-i | i | i | i | s | s |
| Topiramate | n-i | n-i | n-i | n-i | n-i | n-s | n-s |
| Warfarin | i | i | i | n-i | i | n-s | s |
| Estradiol | i | n-i | i | n-i | n-i | n-s | s |
| Cholecalciferol | n-i | n-i | n-i | n-i | n-i | n-s | s |
| Budesonide | n-i | n-i | n-i | n-i | n-i | n-s | s |
| Formoterol | n-i | n-i | n-i | i | n-i | n-s | s |
| Lamotrigine | i | n-i | n-i | n-i | n-i | n-s | n-s |
| Norgestimate | n-i | n-i | n-i | n-i | n-i | n-s | s |
| Quetlapine | i | n-i | n-i | i | i | n-s | s |
| Lorazepam | i | i | i | n-i | n-i | n-s | s |
| Famotidine | n-i | n-i | n-i | n-i | n-i | n-s | n-s |
| Folic Acid | n-i | n-i | n-i | n-i | n-i | n-s | n-s |
| Azithromycin | n-i | n-i | n-i | n-i | n-i | n-s | s |
| Hydroxyzine | n-i | n-i | n-i | i | n-i | s | s |
| Insulin Lispro | n-i | n-i | n-i | n-i | n-i | n-s | s |
| Diclofenac | n-i | n-i | n-i | n-i | n-i | n-s | n-s |
| Loratadine | n-i | i | i | n-i | n-i | n-s | s |
| Sitagliptin | n-i | n-i | n-i | n-i | n-i | n-s | n-s |
| Clonidine | n-i | n-i | n-i | n-i | n-i | s | n-s |
| Diltiazem | n-i | n-i | i | n-i | i | n-s | s |
| Latanoprost | n-i | n-i | i | n-i | i | n-s | s |
| Pregabalin | n-i | n-i | n-i | n-i | n-i | n-s | n-s |
| Doxycycline | n-i | n-i | n-i | n-i | n-i | n-s | n-s |
| Insulin Aspart | n-i | n-i | n-i | n-i | n-i | n-s | s |
| Amitriptyline | n-i | n-i | n-i | i | n-i | n-s | s |
| Paroxetine | i | n-i | n-i | i | n-i | n-s | s |
| Ondansetron | i | n-i | n-i | i | n-i | n-s | n-s |
| Tizanidine | n-i | n-i | n-i | n-i | n-i | s | n-s |
| Lisdexamfetamine | n-i | n-i | n-i | n-i | n-i | n-s | n-s |
| Rivaroxaban | n-i | n-i | i | n-i | i | n-s | s |
| Glimepiride | n-i | i | n-i | n-i | n-i | n-s | s |
| Propranolol | i | n-i | n-i | i | n-i | n-s | s |
| Aripiprazole | n-i | n-i | n-i | i | i | s | s |
| Finasteride | n-i | i | n-i | n-i | n-i | n-s | s |
| Naproxen | n-i | n-i | n-i | n-i | n-i | n-s | n-s |
| Levetiracetam | n-i | n-i | n-i | n-i | n-i | n-s | n-s |
| Alendronate | n-i | n-i | n-i | n-i | n-i | n-s | n-s |
| Fenofibrate | i | n-i | i | n-i | n-i | n-s | s |
| Oxybutynin | i | n-i | n-i | i | i | n-s | s |
| Celecoxib | i | i | i | n-i | i | n-s | s |
| Lovastatin | n-i | n-i | n-i | n-i | n-i | n-s | s |
| Ezetimibe | n-i | i | i | n-i | i | n-s | s |

I (orange): inhibitor; n-i (green): non-inhibitor

**Table S 3. vNN-ADMET Testing Results** (Inhibitors)

| **Drugs** | **1A2** | **2C19** | **2C9** | **2D6** | **3A4** | **Drugs** | **1A2** | **2C19** | **2C9** | **2D6** | **3A4** |
| --- | --- | --- | --- | --- | --- | --- | --- | --- | --- | --- | --- |
| Atorvastatin | - | - | - | - | No | Apixaban | - | - | - | - | - |
| Levothyroxin | No | Yes | No | No | No | Glipizide | No | No | Yes | No | No |
| Metformin | No | No | No | No | No | Spironolactone | No | No | No | No | No |
| Lisinopril | No | No | No | No | No | Cetirizine | No | No | No | No | No |
| Amlodipine | No | Yes | Yes | No | Yes | Atenolol | No | No | No | No | No |
| Metoprolol | No | No | No | No | No | Oxycodone | No | No | No | Yes | No |
| Albuterol | No | No | No | No | No | Buspirone | No | No | No | No | No |
| Omeprazole | - | Yes | No | No | Yes | Salmeterol | No | No | No | Yes | No |
| Losartan | - | - | - | - | No | Topiramate | - | - | - | - | No |
| Gabapentin | No | No | No | No | No | Warfarin | No | No | Yes | No | No |
| Hydrochlorothiazide | No | No | No | No | No | Estradiol | No | No | No | No | No |
| Sertraline | Yes | Yes | No | No | No | Cholecalciferol | No | No | No | No | No |
| Simvastatin | No | No | No | No | Yes | Budesonide | No | No | No | No | Yes |
| Montelukast | - | - | - | - | - | Formoterol | - | Yes | No | Yes | - |
| Escitalopram | No | Yes | Yes | - | No | Lamotrigine | No | No | No | Yes | No |
| Acetaminophen | No | No | No | No | No | Norgestimate | - | - | - | - | - |
| Hydrocodone | - | - | - | - | - | Quetlapine | - | - | - | - | No |
| Rosuvastatin | - | - | - | - | - | Lorazepam | - | - | - | - | - |
| Bupropion | No | - | No | - | No | Famotidine | No | No | No | No | No |
| Furosemide | No | No | No | No | No | Folic Acid | No | No | No | No | No |
| Pantoprazole | - | Yes | No | No | Yes | Azithromycin | No | No | No | No | No |
| Trazodone | No | No | No | No | No | Hydroxyzine | No | No | No | Yes | No |
| Dextroamphetamine | No | No | No | - | No | Insulin Lispro | - | - | - | - | - |
| Dextroamphetamine Saccharate | No | No | No | - | No | Diclofenac | No | No | Yes | No | No |
| Amphetamine Aspartate | No | No | No | - | No | Loratadine | No | Yes | No | - | No |
| Fluticasone | No | No | No | No | Yes | Sitagliptin | - | - | - | - | - |
| Tamsulosin | - | - | - | - | - | Clonidine | No | No | No | Yes | No |
| Fluoxetine | - | Yes | No | Yes | No | Diltiazem | No | No | No | No | Yes |
| Carvedilol | - | - | - | - | Yes | Latanoprost | - | - | - | - | - |
| Duloxetine | - | - | - | - | - | Pregabalin | - | - | - | - | No |
| Meloxicam | No | No | Yes | No | No | Doxycycline | No | No | No | No | No |
| Clopidogrel | - | - | - | - | No | Insulin Aspart | - | - | - | - | - |
| Prednisone | No | No | No | No | No | Amitriptyline | No | No | No | Yes | No |
| Citalopram | No | Yes | Yes | - | No | Paroxetine | No | No | - | No | No |
| Insulin Glargine | - | - | - | - | - | Ondansetron | No | No | No | - | - |
| Potassium Chloride | - | - | - | - | - | Tizanidine | No | No | No | - | - |
| Pravastatin | - | - | - | - | No | Lisdexamfetamine | - | - | - | - | - |
| Tramadol | - | - | - | - | - | Rivaroxaban | - | - | - | - | - |
| Aspirin | - | - | - | - | - | Glimepiride | No | Yes | No | No | No |
| Alprazolam | - | - | - | - | - | Propranolol | Yes | No | No | Yes | No |
| Ibuprofen | No | No | - | No | No | Aripiprazole | - | - | - | - | - |
| Cyclobenzaprine | No | No | No | Yes | No | Finasteride | No | Yes | - | No | - |
| Amoxicillin | No | No | No | No | No | Naproxen | No | No | No | No | No |
| Methylphenidate | - | - | - | - | No | Levetiracetam | - | - | - | - | - |
| Allopurinol | - | - | - | - | - | Alendronate | - | - | - | - | No |
| Venlafaxine | - | - | - | - | No | Fenofibrate | - | No | Yes | No | No |
| Clonazepam | - | - | - | - | - | Oxybutynin | Yes | - | Yes | Yes | No |
| Ethinyl Estradiol | Yes | No | No | No | No | Celecoxib | - | - | - | - | No |
| Ergocalciferol | No | No | No | No | No | Lovastatin | No | No | No | No | No |
| Zolpidem | - | - | - | - | - | Ezetimibe | - | - | - | - | No |

Yes (orange): inhibitor; No (green): non-inhibitor

**Table S 4. SuperCYPsPred Testing Results** (Inhibitors)

| **Drugs** | **Fingerprint** | **1A2** | **2C9** | **2C19** | **2D6** | **3A4** |
| --- | --- | --- | --- | --- | --- | --- |
| Atorvastatin | MACCS | Inactive (0.955) | Inactive (0.571) | Inactive (0.841) | Inactive (0.771) | Inactive (0.73) |
|  | Morgan | Inactive (0.749) | Inactive (0.655) | Inactive (0.809) | Inactive (0.651) | Inactive (0.688) |
| Levothyroxin | MACCS | Inactive (0.947) | Active (0.502) | Inactive (0.926) | Inactive (0.711) | Inactive (0.976) |
|  | Morgan | Inactive (0.97) | Active (0.848) | Inactive (0.984) | Inactive (0.801) | Inactive (0.866) |
| Metformin | MACCS | Inactive (0.924) | Inactive (0.841) | Inactive (0.83) | Inactive (0.656) | Inactive (0.998) |
|  | Morgan | Inactive (0.99) | Inactive (0.98) | Inactive (0.974) | Inactive (0.759) | Inactive (0.933) |
| Lisinopril | MACCS | Inactive (0.995) | Inactive (0.965) | Inactive (0.957) | Inactive (0.928) | Inactive (0.994) |
|  | Morgan | Inactive (0.956) | Inactive (0.951) | Inactive (0.96) | Active (0.504) | Inactive (0.878) |
| Amlodipine | MACCS | Inactive (0.806) | Active (0.669) | Inactive (0.614) | Active (0.501) | Active (0.545) |
|  | Morgan | Inactive (0.723) | Active (0.781) | Active (0.676) | Active (0.588) | Active (0.826) |
| Metoprolol | MACCS | Inactive (0.894) | Inactive (0.874) | Inactive (0.982) | Active (0.585) | Inactive (0.986) |
|  | Morgan | Inactive (0.875) | Inactive (0.928) | Inactive (0.955) | Inactive (0.599) | Inactive (0.717) |
| Albuterol | MACCS | Inactive (0.971) | Inactive (0.964) | Inactive (0.983) | Inactive (0.594) | Inactive (0.999) |
|  | Morgan | Inactive (0.933) | Inactive (0.975) | Inactive (0.91) | Active (0.562) | Inactive (0.885) |
| Omeprazole | MACCS | Inactive (0.593) | Inactive (0.597) | Active (0.991) | Inactive (0.543) | Active (0.922) |
|  | Morgan | Active (0.603) | Inactive (0.914) | Active (0.704) | Inactive (0.553) | Active (0.848) |
| Losartan | MACCS | Inactive (0.799) | Active (0.507) | Inactive (0.683) | Active (0.512) | Inactive (0.899) |
|  | Morgan | Inactive (0.676) | Inactive (0.73) | Inactive (0.651) | Active (0.651) | Active (0.53) |
| Gabapentin | MACCS | Inactive (0.952) | Inactive (0.897) | Inactive (0.883) | Inactive (0.646) | Inactive (0.992) |
|  | Morgan | Inactive (0.993) | Inactive (0.975) | Inactive (0.97) | Inactive (0.84) | Inactive (0.954) |
| Hydrochlorothiazide | MACCS | Inactive (0.933) | Inactive (0.66) | Inactive (0.924) | Inactive (0.854) | Inactive (0.985) |
|  | Morgan | Inactive (0.992) | Inactive (0.985) | Inactive (0.975) | Inactive (0.7) | Inactive (0.846) |
| Sertraline | MACCS | Inactive (0.789) | Inactive (0.597) | Active (0.703) | Active (0.654) | Active (0.536) |
|  | Morgan | Inactive (0.7) | Inactive (0.825) | Active (0.52) | Active (0.702) | Active (0.868) |
| Simvastatin | MACCS | Inactive (0.995) | Inactive (0.962) | Inactive (0.995) | Inactive (0.959) | Active (0.999) |
|  | Morgan | Inactive (0.998) | Inactive (0.994) | Inactive (0.995) | Inactive (0.941) | Active (0.875) |
| Montelukast | MACCS | Inactive (0.901) | Active (0.7) | Inactive (0.733) | Inactive (0.706) | Inactive (0.667) |
|  | Morgan | Inactive (0.866) | Inactive (0.724) | Inactive (0.631) | Active (0.544) | Inactive (0.555) |
| Escitalopram | MACCS | Inactive (0.938) | Active (0.807) | Active (0.999) | Active (0.837) | Inactive (0.953) |
|  | Morgan | Inactive (0.896) | Active (0.836) | Active (0.71) | Active (0.538) | Inactive (0.665) |
| Acetaminophen | MACCS | Inactive (0.671) | Inactive (0.558) | Inactive (0.748) | Inactive (0.934) | Inactive (0.542) |
|  | Morgan | Inactive (0.87) | Inactive (0.62) | Inactive (0.979) | Inactive (0.826) | Inactive (0.862) |
| Hydrocodone | MACCS | Inactive (0.913) | Inactive (0.721) | Inactive (0.86) | Active (0.723) | Inactive (0.881) |
|  | Morgan | Inactive (0.858) | Inactive (0.843) | Inactive (0.918) | Inactive (0.502) | Inactive (0.705) |
| Rosuvastatin | MACCS | Inactive (0.881) | Active (0.522) | Inactive (0.808) | Inactive (0.737) | Inactive (0.747) |
|  | Morgan | Inactive (0.732) | Inactive (0.69) | Inactive (0.823) | Inactive (0.719) | Inactive (0.693) |
| Bupropion | MACCS | Inactive (0.699) | Inactive (0.581) | Active (0.576) | Inactive (0.635) | Inactive (0.87) |
|  | Morgan | Inactive (0.679) | Inactive (0.831) | Inactive (0.876) | Inactive (0.623) | Inactive (0.772) |
| Furosemide | MACCS | Inactive (0.952) | Inactive (0.567) | Inactive (0.825) | Inactive (0.774) | Inactive (0.983) |
|  | Morgan | Inactive (0.946) | Inactive (0.933) | Inactive (0.942) | Inactive (0.666) | Inactive (0.816) |
| Pantoprazole | MACCS | Inactive (0.624) | Inactive (0.517) | Active (0.843) | Inactive (0.583) | Active (0.666) |
|  | Morgan | Active (0.572) | Inactive (0.826) | Inactive (0.64) | Inactive (0.578) | Active (0.656) |
| Trazodone | MACCS | Inactive (0.691) | Active (0.507) | Inactive (0.791) | Inactive (0.586) | Inactive (0.956) |
|  | Morgan | Inactive (0.908) | Inactive (0.962) | Inactive (0.924) | Active (0.762) | Inactive (0.714) |
| Dextroamphetamine | MACCS | Inactive (0.629) | Inactive (0.808) | Inactive (0.694) | Active (0.64) | Inactive (0.941) |
|  | Morgan | Active (0.552) | Inactive (0.823) | Inactive (0.926) | Active (0.723) | Inactive (0.933) |
| Dextroamphetamine Saccharate | MACCS | Inactive (0.981) | Inactive (0.762) | Inactive (0.968) | Inactive (0.836) | Inactive (0.981) |
|  | Morgan | Inactive (0.871) | Inactive (0.859) | Inactive (0.88) | Inactive (0.739) | Inactive (0.923) |
| Amphetamine Aspartate | MACCS | Inactive (0.952) | Inactive (0.753) | Inactive (0.916) | Inactive (0.839) | Inactive (0.915) |
|  | Morgan | Inactive (0.952) | Inactive (0.905) | Inactive (0.966) | Inactive (0.819) | Inactive (0.945) |
| Fluticasone | MACCS | Inactive (0.995) | Active (0.508) | Inactive (0.897) | Inactive (0.677) | Active (0.78) |
|  | Morgan | Inactive (0.985) | Inactive (0.96) | Inactive (0.986) | Inactive (0.638) | Active (0.519) |
| Tamsulosin | MACCS | Inactive (0.859) | Active (0.528) | Inactive (0.528) | Inactive (0.52) | Active (0.61) |
|  | Morgan | Inactive (0.801) | Inactive (0.751) | Inactive (0.684) | Active (0.616) | Active (0.614) |
| Fluoxetine | MACCS | Inactive (0.516) | Inactive (0.522) | Active (0.993) | Active (0.95) | Active (0.513) |
|  | Morgan | Inactive (0.738) | Inactive (0.85) | Active (0.774) | Active (0.965) | Active (0.797) |
| Carvedilol | MACCS | Inactive (0.714) | Inactive (0.8) | Inactive (0.678) | Active (0.783) | Active (0.841) |
|  | Morgan | Active (0.647) | Inactive (0.929) | Inactive (0.698) | Active (0.794) | Active (0.827) |
| Duloxetine | MACCS | Active (0.555) | Inactive (0.609) | Inactive (0.51) | Active (0.86) | Inactive (0.612) |
|  | Morgan | Active (0.547) | Inactive (0.813) | Inactive (0.705) | Active (0.825) | Inactive (0.547) |
| Meloxicam | MACCS | Inactive (0.967) | Active (0.751) | Inactive (0.963) | Inactive (0.87) | Inactive (0.981) |
|  | Morgan | Inactive (0.894) | Inactive (0.554) | Inactive (0.93) | Inactive (0.622) | Inactive (0.688) |
| Clopidogrel | MACCS | Inactive (0.582) | Active (0.668) | Active (0.505) | Active (0.82) | Inactive (0.855) |
|  | Morgan | Inactive (0.639) | Inactive (0.748) | Inactive (0.714) | Active (0.581) | Inactive (0.631) |
| Prednisone | MACCS | Inactive (1.0) | Inactive (0.936) | Inactive (1.0) | Inactive (0.877) | Inactive (1.0) |
|  | Morgan | Inactive (0.981) | Inactive (0.943) | Inactive (0.965) | Inactive (0.787) | Inactive (0.819) |
| Citalopram | MACCS | Inactive (0.938) | Active (0.807) | Active (0.999) | Active (0.837) | Inactive (0.953) |
|  | Morgan | Inactive (0.896) | Active (0.836) | Active (0.71) | Active (0.538) | Inactive (0.665) |
| Insulin Glargine | MACCS | Inactive (0.957) | Inactive (0.75) | Inactive (0.912) | Inactive (0.815) | Inactive (0.829) |
|  | Morgan | Inactive (0.914) | Inactive (0.818) | Inactive (0.755) | Inactive (0.592) | Inactive (0.541) |
| Pravastatin | MACCS | Inactive (0.971) | Inactive (0.939) | Inactive (0.864) | Inactive (0.867) | Inactive (0.981) |
|  | Morgan | Inactive (0.952) | Inactive (0.886) | Inactive (0.918) | Inactive (0.79) | Inactive (0.857) |
| Tramadol | MACCS | Inactive (0.879) | Inactive (0.62) | Inactive (0.703) | Active (0.687) | Inactive (0.801) |
|  | Morgan | Inactive (0.826) | Inactive (0.73) | Inactive (0.705) | Active (0.744) | Inactive (0.556) |
| Aspirin | MACCS | Inactive (0.869) | Inactive (0.531) | Inactive (0.828) | Inactive (0.679) | Inactive (0.994) |
|  | Morgan | Inactive (0.903) | Inactive (0.872) | Inactive (0.972) | Inactive (0.926) | Inactive (0.978) |
| Alprazolam | MACCS | Active (0.623) | Inactive (0.541) | Inactive (0.641) | Active (0.58) | Inactive (0.595) |
|  | Morgan | Active (0.682) | Inactive (0.721) | Inactive (0.761) | Active (0.739) | Inactive (0.678) |
| Ibuprofen | MACCS | Inactive (0.929) | Inactive (0.626) | Inactive (0.835) | Inactive (0.961) | Inactive (0.998) |
|  | Morgan | Inactive (0.981) | Inactive (0.766) | Inactive (0.962) | Inactive (0.842) | Inactive (0.932) |
| Cyclobenzaprine | MACCS | Inactive (0.636) | Inactive (0.532) | Inactive (0.598) | Active (0.993) | Inactive (0.98) |
|  | Morgan | Inactive (0.75) | Inactive (0.944) | Inactive (0.982) | Active (0.977) | Inactive (0.802) |
| Amoxicillin | MACCS | Inactive (1.0) | Inactive (0.84) | Inactive (0.992) | Inactive (0.953) | Inactive (0.997) |
|  | Morgan | Inactive (0.995) | Inactive (0.983) | Inactive (0.989) | Inactive (0.865) | Inactive (0.939) |
| Methylphenidate | MACCS | Inactive (0.759) | Inactive (0.76) | Inactive (0.768) | Active (0.659) | Inactive (0.804) |
|  | Morgan | Inactive (0.771) | Inactive (0.806) | Inactive (0.885) | Active (0.544) | Inactive (0.594) |
| Allopurinol | MACCS | Inactive (0.652) | Inactive (0.631) | Inactive (0.664) | Inactive (0.772) | Inactive (0.891) |
|  | Morgan | Inactive (0.771) | Inactive (0.9) | Inactive (0.974) | Inactive (0.768) | Inactive (0.883) |
| Venlafaxine | MACCS | Inactive (0.856) | Inactive (0.669) | Inactive (0.751) | Active (0.747) | Inactive (0.918) |
|  | Morgan | Inactive (0.836) | Inactive (0.737) | Inactive (0.764) | Active (0.68) | Inactive (0.855) |
| Clonazepam | MACCS | Inactive (0.592) | Inactive (0.513) | Inactive (0.657) | Inactive (0.659) | Inactive (0.658) |
|  | Morgan | Active (0.597) | Inactive (0.57) | Inactive (0.683) | Active (0.506) | Inactive (0.614) |
| Ethinyl Estradiol | MACCS | Inactive (0.665) | Active (0.702) | Inactive (0.781) | Inactive (0.814) | Inactive (0.99) |
|  | Morgan | Inactive (0.636) | Inactive (0.688) | Inactive (0.661) | Active (0.593) | Inactive (0.735) |
| Ergocalciferol | MACCS | Inactive (0.998) | Active (0.744) | Inactive (0.992) | Inactive (0.933) | Inactive (0.997) |
|  | Morgan | Inactive (0.996) | Inactive (0.975) | Inactive (0.995) | Inactive (0.806) | Inactive (0.902) |
| Zolpidem | MACCS | Inactive (0.629) | Inactive (0.601) | Inactive (0.858) | Inactive (0.708) | Inactive (0.658) |
|  | Morgan | Inactive (0.686) | Inactive (0.711) | Inactive (0.886) | Inactive (0.714) | Inactive (0.704) |
| Apixaban | MACCS | Inactive (0.854) | Inactive (0.644) | Inactive (0.839) | Inactive (0.738) | Inactive (0.605) |
|  | Morgan | Inactive (0.728) | Inactive (0.793) | Inactive (0.753) | Inactive (0.612) | Inactive (0.63) |
| Glipizide | MACCS | Inactive (0.879) | Inactive (0.645) | Inactive (0.809) | Inactive (0.931) | Inactive (0.845) |
|  | Morgan | Inactive (0.972) | Active (0.593) | Inactive (0.963) | Inactive (0.698) | Inactive (0.799) |
| Spironolactone | MACCS | Inactive (0.984) | Inactive (0.634) | Inactive (0.913) | Inactive (0.9) | Inactive (0.958) |
|  | Morgan | Inactive (0.961) | Inactive (0.981) | Inactive (0.947) | Inactive (0.828) | Inactive (0.887) |
| Cetirizine | MACCS | Inactive (0.971) | Active (0.611) | Inactive (0.92) | Active (0.848) | Inactive (0.984) |
|  | Morgan | Inactive (0.989) | Inactive (0.908) | Inactive (0.868) | Active (0.862) | Inactive (0.856) |
| Atenolol | MACCS | Inactive (0.867) | Inactive (0.896) | Inactive (0.943) | Active (0.611) | Inactive (0.983) |
|  | Morgan | Inactive (0.847) | Inactive (0.973) | Inactive (0.954) | Inactive (0.804) | Inactive (0.758) |
| Oxycodone | MACCS | Inactive (0.968) | Inactive (0.823) | Inactive (0.927) | Active (0.67) | Inactive (0.886) |
|  | Morgan | Inactive (0.956) | Inactive (0.817) | Inactive (0.943) | Active (0.651) | Inactive (0.742) |
| Buspirone | MACCS | Inactive (0.929) | Inactive (0.66) | Inactive (0.806) | Inactive (0.689) | Inactive (0.815) |
|  | Morgan | Inactive (0.962) | Inactive (0.953) | Inactive (0.912) | Inactive (0.528) | Inactive (0.784) |
| Salmeterol | MACCS | Inactive (0.866) | Inactive (0.753) | Inactive (0.908) | Active (0.874) | Inactive (0.908) |
|  | Morgan | Inactive (0.892) | Inactive (0.937) | Inactive (0.879) | Active (0.877) | Inactive (0.821) |
| Topiramate | MACCS | Inactive (0.898) | Inactive (0.771) | Inactive (0.82) | Inactive (0.688) | Inactive (0.927) |
|  | Morgan | Inactive (0.952) | Inactive (0.932) | Inactive (0.927) | Inactive (0.834) | Inactive (0.941) |
| Warfarin | MACCS | Inactive (0.847) | Active (0.778) | Inactive (0.907) | Inactive (0.885) | Inactive (0.962) |
|  | Morgan | Inactive (0.835) | Active (0.868) | Inactive (0.915) | Inactive (0.697) | Inactive (0.757) |
| Estradiol | MACCS | Inactive (0.704) | Active (0.694) | Inactive (0.927) | Inactive (0.836) | Inactive (0.998) |
|  | Morgan | Inactive (0.531) | Inactive (0.954) | Inactive (0.973) | Active (0.533) | Inactive (0.977) |
| Cholecalciferol | MACCS | Inactive (0.999) | Active (0.688) | Inactive (0.991) | Inactive (0.945) | Inactive (0.989) |
|  | Morgan | Inactive (0.994) | Inactive (0.954) | Inactive (0.986) | Inactive (0.955) | Inactive (0.813) |
| Budesonide | MACCS | Inactive (0.999) | Inactive (0.929) | Inactive (0.994) | Inactive (0.892) | Active (0.738) |
|  | Morgan | Inactive (0.993) | Inactive (0.971) | Inactive (0.995) | Inactive (0.746) | Active (0.89) |
| Formoterol | MACCS | Inactive (0.686) | Inactive (0.732) | Inactive (0.811) | Active (0.778) | Inactive (0.753) |
|  | Morgan | Inactive (0.77) | Inactive (0.949) | Inactive (0.665) | Active (0.755) | Active (0.578) |
| Lamotrigine | MACCS | Inactive (0.657) | Inactive (0.637) | Inactive (0.631) | Active (0.746) | Inactive (0.94) |
|  | Morgan | Inactive (0.835) | Inactive (0.911) | Inactive (0.918) | Active (0.805) | Inactive (0.914) |
| Norgestimate | MACCS | Inactive (0.916) | Inactive (0.596) | Inactive (0.709) | Inactive (0.691) | Inactive (0.774) |
|  | Morgan | Inactive (0.89) | Inactive (0.796) | Inactive (0.686) | Inactive (0.682) | Inactive (0.659) |
| Quetlapine | MACCS | Inactive (0.618) | Inactive (0.679) | Inactive (0.689) | Active (0.77) | Inactive (0.745) |
|  | Morgan | Inactive (0.698) | Inactive (0.796) | Inactive (0.784) | Active (0.822) | Inactive (0.711) |
| Lorazepam | MACCS | Active (0.53) | Inactive (0.511) | Inactive (0.731) | Inactive (0.598) | Inactive (0.804) |
|  | Morgan | Inactive (0.523) | Inactive (0.644) | Inactive (0.737) | Inactive (0.655) | Inactive (0.622) |
| Famotidine | MACCS | Inactive (0.849) | Inactive (0.706) | Inactive (0.93) | Inactive (0.585) | Inactive (0.97) |
|  | Morgan | Inactive (0.93) | Inactive (0.97) | Inactive (0.954) | Inactive (0.843) | Inactive (0.8) |
| Folic Acid | MACCS | Inactive (0.976) | Inactive (0.788) | Inactive (0.952) | Inactive (0.816) | Inactive (0.987) |
|  | Morgan | Inactive (0.973) | Inactive (0.963) | Inactive (0.964) | Inactive (0.932) | Inactive (0.876) |
| Azithromycin | MACCS | Inactive (0.989) | Inactive (0.825) | Inactive (0.946) | Inactive (0.682) | Inactive (0.916) |
|  | Morgan | Inactive (0.969) | Inactive (0.969) | Inactive (0.935) | Inactive (0.88) | Inactive (0.707) |
| Hydroxyzine | MACCS | Inactive (0.954) | Active (0.588) | Inactive (0.793) | Active (0.949) | Inactive (0.97) |
|  | Morgan | Inactive (0.982) | Inactive (0.944) | Inactive (0.85) | Active (0.968) | Inactive (0.834) |
| Insulin Lispro | MACCS | Inactive (0.951) | Inactive (0.688) | Inactive (0.862) | Inactive (0.794) | Inactive (0.809) |
|  | Morgan | Inactive (0.92) | Inactive (0.847) | Inactive (0.753) | Inactive (0.648) | Inactive (0.557) |
| Diclofenac | MACCS | Inactive (0.595) | Active (0.885) | Inactive (0.902) | Inactive (0.891) | Inactive (0.974) |
|  | Morgan | Inactive (0.885) | Active (0.962) | Inactive (0.911) | Inactive (0.768) | Inactive (0.783) |
| Loratadine | MACCS | Inactive (0.785) | Active (0.713) | Active (0.984) | Active (0.688) | Inactive (0.859) |
|  | Morgan | Inactive (0.891) | Inactive (0.872) | Active (0.693) | Active (0.512) | Inactive (0.594) |
| Sitagliptin | MACCS | Inactive (0.821) | Active (0.534) | Inactive (0.708) | Active (0.739) | Inactive (0.603) |
|  | Morgan | Inactive (0.864) | Inactive (0.781) | Inactive (0.844) | Inactive (0.587) | Inactive (0.746) |
| Clonidine | MACCS | Inactive (0.745) | Inactive (0.645) | Inactive (0.87) | Active (0.889) | Inactive (0.993) |
|  | Morgan | Inactive (0.825) | Inactive (0.879) | Inactive (0.866) | Active (0.883) | Inactive (0.832) |
| Diltiazem | MACCS | Inactive (0.91) | Active (0.638) | Inactive (0.892) | Inactive (0.928) | Active (0.518) |
|  | Morgan | Inactive (0.957) | Inactive (0.975) | Inactive (0.895) | Inactive (0.795) | Inactive (0.509) |
| Latanoprost | MACCS | Inactive (0.886) | Inactive (0.719) | Inactive (0.834) | Inactive (0.837) | Inactive (0.809) |
|  | Morgan | Inactive (0.792) | Inactive (0.662) | Inactive (0.762) | Active (0.514) | Active (0.506) |
| Pregabalin | MACCS | Inactive (0.975) | Inactive (0.846) | Inactive (0.913) | Inactive (0.808) | Inactive (1.0) |
|  | Morgan | Inactive (0.989) | Inactive (0.938) | Inactive (0.979) | Inactive (0.879) | Inactive (0.992) |
| Doxycycline | MACCS | Inactive (0.996) | Inactive (0.771) | Inactive (1.0) | Inactive (0.866) | Inactive (1.0) |
|  | Morgan | Inactive (0.977) | Inactive (0.974) | Inactive (0.975) | Inactive (0.799) | Inactive (0.896) |
| Insulin Aspart | MACCS | Inactive (0.949) | Inactive (0.75) | Inactive (0.902) | Inactive (0.828) | Inactive (0.865) |
|  | Morgan | Inactive (0.926) | Inactive (0.812) | Inactive (0.73) | Inactive (0.582) | Inactive (0.544) |
| Amitriptyline | MACCS | Inactive (0.636) | Inactive (0.532) | Inactive (0.598) | Active (0.993) | Inactive (0.98) |
|  | Morgan | Inactive (0.903) | Inactive (0.939) | Inactive (0.96) | Active (0.985) | Inactive (0.829) |
| Paroxetine | MACCS | Inactive (0.865) | Active (0.556) | Inactive (0.811) | Active (0.857) | Active (0.545) |
|  | Morgan | Inactive (0.817) | Inactive (0.614) | Inactive (0.862) | Active (0.845) | Active (0.549) |
| Ondansetron | MACCS | Inactive (0.84) | Inactive (0.548) | Inactive (0.833) | Active (0.621) | Inactive (0.58) |
|  | Morgan | Inactive (0.85) | Inactive (0.915) | Inactive (0.934) | Inactive (0.515) | Inactive (0.637) |
| Tizanidine | MACCS | Inactive (0.638) | Inactive (0.523) | Inactive (0.689) | Active (0.647) | Inactive (0.813) |
|  | Morgan | Inactive (0.752) | Inactive (0.841) | Inactive (0.852) | Active (0.713) | Inactive (0.754) |
| Lisdexamfetamine | MACCS | Inactive (0.772) | Inactive (0.832) | Inactive (0.866) | Inactive (0.562) | Inactive (0.833) |
|  | Morgan | Inactive (0.801) | Inactive (0.826) | Inactive (0.805) | Inactive (0.532) | Inactive (0.721) |
| Rivaroxaban | MACCS | Inactive (0.9) | Inactive (0.547) | Inactive (0.698) | Inactive (0.615) | Inactive (0.542) |
|  | Morgan | Inactive (0.838) | Inactive (0.775) | Inactive (0.833) | Inactive (0.639) | Inactive (0.641) |
| Glimepiride | MACCS | Inactive (0.925) | Active (0.803) | Inactive (0.842) | Inactive (0.785) | Inactive (0.853) |
|  | Morgan | Inactive (0.967) | Active (0.898) | Inactive (0.942) | Inactive (0.677) | Inactive (0.523) |
| Propranolol | MACCS | Active (0.999) | Inactive (0.794) | Inactive (0.949) | Active (0.972) | Inactive (0.987) |
|  | Morgan | Active (1.0) | Inactive (0.98) | Inactive (0.946) | Active (0.971) | Inactive (0.841) |
| Aripiprazole | MACCS | Inactive (0.861) | Active (0.575) | Inactive (0.824) | Active (0.757) | Inactive (0.598) |
|  | Morgan | Inactive (0.843) | Inactive (0.788) | Inactive (0.729) | Active (0.699) | Inactive (0.568) |
| Finasteride | MACCS | Inactive (0.988) | Inactive (0.62) | Active (0.985) | Inactive (0.624) | Inactive (0.829) |
|  | Morgan | Inactive (0.949) | Inactive (0.591) | Active (0.669) | Inactive (0.817) | Inactive (0.759) |
| Naproxen | MACCS | Inactive (0.884) | Active (0.654) | Inactive (0.971) | Inactive (0.885) | Inactive (0.994) |
|  | Morgan | Inactive (0.865) | Inactive (0.832) | Inactive (0.894) | Inactive (0.918) | Inactive (0.827) |
| Levetiracetam | MACCS | Inactive (0.902) | Inactive (0.812) | Inactive (0.738) | Inactive (0.69) | Inactive (0.95) |
|  | Morgan | Inactive (0.943) | Inactive (0.966) | Inactive (0.962) | Inactive (0.728) | Inactive (0.934) |
| Alendronate | MACCS | Inactive (0.992) | Inactive (0.904) | Inactive (0.96) | Inactive (0.76) | Inactive (1.0) |
|  | Morgan | Inactive (0.99) | Inactive (0.948) | Inactive (0.96) | Inactive (0.922) | Inactive (0.975) |
| Fenofibrate | MACCS | Inactive (0.855) | Active (0.891) | Inactive (0.744) | Inactive (0.769) | Inactive (0.918) |
|  | Morgan | Inactive (0.76) | Active (0.844) | Inactive (0.949) | Inactive (0.714) | Inactive (0.771) |
| Oxybutynin | MACCS | Active (0.712) | Inactive (0.635) | Active (0.903) | Active (0.879) | Inactive (0.857) |
|  | Morgan | Active (0.88) | Inactive (0.869) | Active (0.658) | Active (0.908) | Active (0.558) |
| Celecoxib | MACCS | Inactive (0.758) | Active (0.713) | Inactive (0.562) | Inactive (0.582) | Inactive (0.779) |
|  | Morgan | Inactive (0.694) | Inactive (0.697) | Inactive (0.695) | Inactive (0.721) | Inactive (0.737) |
| Lovastatin | MACCS | Inactive (0.995) | Inactive (0.964) | Inactive (0.988) | Inactive (0.905) | Inactive (0.999) |
|  | Morgan | Inactive (0.992) | Inactive (0.996) | Inactive (0.989) | Inactive (0.896) | Inactive (0.854) |
| Ezetimibe | MACCS | Inactive (0.782) | Active (0.577) | Inactive (0.745) | Inactive (0.638) | Inactive (0.956) |
|  | Morgan | Inactive (0.866) | Inactive (0.79) | Inactive (0.845) | Inactive (0.768) | Inactive (0.729) |

Active (orange): inhibitor; Inactive (green): non-inhibitor

**Table S 5. SwissADME Testing Results** (Inhibitors)

| **Drugs** | **1A2** | **2C19** | **2C9** | **2D6** | **3A4** | **Drugs** | **1A2** | **2C19** | **2C9** | **2D6** | **3A4** |
| --- | --- | --- | --- | --- | --- | --- | --- | --- | --- | --- | --- |
| Atorvastatin | No | Yes | No | Yes | Yes | Apixaban | No | Yes | Yes | Yes | Yes |
| Levothyroxin | No | No | Yes | No | No | Glipizide | No | No | Yes | Yes | Yes |
| Metformin | No | No | No | No | No | Spironolactone | No | No | No | No | No |
| Lisinopril | No | No | No | No | No | Cetirizine | No | No | No | Yes | No |
| Amlodipine | Yes | Yes | Yes | No | Yes | Atenolol | No | No | No | No | No |
| Metoprolol | No | No | No | Yes | No | Oxycodone | No | No | No | Yes | No |
| Albuterol | No | No | No | No | No | Buspirone | Yes | Yes | Yes | No | No |
| Omeprazole | Yes | Yes | No | Yes | Yes | Salmeterol | No | No | No | No | Yes |
| Losartan | No | Yes | Yes | Yes | Yes | Topiramate | No | No | No | No | No |
| Gabapentin | No | No | No | No | No | Warfarin | No | Yes | Yes | No | No |
| Hydrochlorothiazide | No | No | No | No | No | Estradiol | No | No | No | Yes | No |
| Sertraline | Yes | Yes | No | Yes | No | Cholecalciferol | No | No | Yes | No | No |
| Simvastatin | No | No | Yes | No | Yes | Budesonide | No | No | No | No | No |
| Montelukast | No | Yes | No | Yes | Yes | Formoterol | No | No | No | Yes | No |
| Escitalopram | No | Yes | No | Yes | Yes | Lamotrigine | Yes | No | No | No | No |
| Acetaminophen | No | No | No | No | No | Norgestimate | No | No | Yes | No | No |
| Hydrocodone | No | No | No | Yes | No | Quetlapine | No | No | No | Yes | Yes |
| Rosuvastatin | No | No | No | No | No | Lorazepam | No | No | No | No | No |
| Bupropion | Yes | No | No | Yes | No | Famotidine | No | No | No | No | No |
| Furosemide | No | No | No | No | No | Folic Acid | No | No | No | No | No |
| Pantoprazole | Yes | Yes | Yes | Yes | Yes | Azithromycin | No | No | No | No | No |
| Trazodone | No | No | Yes | Yes | No | Hydroxyzine | No | No | No | Yes | No |
| Dextroamphetamine | Yes | No | No | No | No | Diclofenac | Yes | Yes | Yes | Yes | No |
| Dextroamphetamine Saccharate | No | No | No | No | No | Loratadine | Yes | Yes | Yes | Yes | Yes |
| Amphetamine Aspartate | No | No | No | No | No | Sitagliptin | No | No | No | No | No |
| Fluticasone | No | No | No | No | Yes | Clonidine | No | No | No | No | No |
| Tamsulosin | No | Yes | Yes | Yes | Yes | Diltiazem | No | Yes | Yes | Yes | Yes |
| Fluoxetine | Yes | Yes | No | Yes | Yes | Latanoprost | No | No | No | Yes | Yes |
| Carvedilol | Yes | Yes | Yes | Yes | Yes | Pregabalin | No | No | No | No | No |
| Duloxetine | Yes | Yes | Yes | Yes | Yes | Doxycycline | No | No | No | No | No |
| Meloxicam | No | No | Yes | No | Yes | Amitriptyline | No | No | No | Yes | No |
| Clopidogrel | No | Yes | Yes | Yes | No | Paroxetine | No | Yes | No | Yes | Yes |
| Prednisone | No | No | No | No | No | Ondansetron | Yes | Yes | No | Yes | Yes |
| Citalopram | No | No | No | Yes | Yes | Tizanidine | Yes | No | No | No | No |
| Pravastatin | No | No | No | No | Yes | Lisdexamfetamine | No | No | No | Yes | No |
| Tramadol | No | No | No | Yes | No | Rivaroxaban | No | Yes | Yes | No | Yes |
| Aspirin | No | No | No | No | No | Glimepiride | No | No | Yes | No | Yes |
| Alprazolam | Yes | No | No | No | No | Propranolol | Yes | No | No | Yes | No |
| Ibuprofen | No | No | No | No | No | Aripiprazole | No | Yes | No | Yes | No |
| Cyclobenzaprine | No | No | No | Yes | No | Finasteride | No | No | No | No | No |
| Amoxicillin | No | No | No | No | No | Naproxen | No | No | No | No | No |
| Methylphenidate | No | No | No | No | No | Levetiracetam | No | No | No | No | No |
| Allopurinol | No | No | No | No | No | Alendronate | No | No | No | No | No |
| Venlafaxine | No | No | No | Yes | No | Fenofibrate | Yes | Yes | Yes | Yes | No |
| Clonazepam | Yes | Yes | No | No | Yes | Oxybutynin | No | No | No | Yes | No |
| Ethinyl Estradiol | No | No | Yes | Yes | No | Celecoxib | Yes | No | Yes | No | No |
| Ergocalciferol | No | No | Yes | No | No | Lovastatin | No | No | Yes | No | Yes |
| Zolpidem | Yes | Yes | Yes | Yes | Yes | Ezetimibe | No | Yes | No | Yes | Yes |

Yes (orange): inhibitor; No (green): non-inhibitor

**Table S 6. CYPStrate Testing Results** (Substrates)

| **Drugs** | **1A2** | **2A6** | **2B6** | **2C8** | **2C9** | **2C19** | **2D6** | **2E1** | **3A4** |
| --- | --- | --- | --- | --- | --- | --- | --- | --- | --- |
| Atorvastatin | No | No | No | Yes | - | No | No | No | Yes |
| Levothyroxin | No | No | No | No | - | No | No | No | No |
| Metformin | No | No | No | No | No | No | No | No | No |
| Lisinopril | No | No | No | No | No | No | No | No | No |
| Amlodipine | No | No | - | - | No | No | - | No | Yes |
| Metoprolol | - | No | - | No | No | Yes | Yes | No | No |
| Albuterol | No | No | No | No | No | - | No | No | - |
| Omeprazole | - | No | No | Yes | Yes | Yes | - | No | Yes |
| Losartan | - | No | No | No | Yes | No | - | No | Yes |
| Gabapentin | No | No | No | No | No | No | No | No | No |
| Hydrochlorothiazide | No | No | - | - | - | - | No | No | - |
| Sertraline | Yes | No | Yes | Yes | Yes | - | Yes | Yes | Yes |
| Simvastatin | No | No | No | No | No | No | No | No | Yes |
| Montelukast | - | No | - | Yes | Yes | - | - | No | - |
| Escitalopram | No | No | - | - | - | Yes | Yes | No | Yes |
| Acetaminophen | Yes | No | No | - | - | No | - | Yes | - |
| Hydrocodone | No | No | - | - | No | - | Yes | No | Yes |
| Rosuvastatin | No | No | No | - | - | - | No | No | - |
| Bupropion | - | - | Yes | - | - | - | - | Yes | Yes |
| Furosemide | No | No | No | - | - | - | - | No | No |
| Pantoprazole | - | No | No | - | - | Yes | - | No | Yes |
| Trazodone | - | No | - | - | - | - | - | No | Yes |
| Dextroamphetamine | - | - | - | No | No | No | - | - | No |
| Dextroamphetamine Saccharate | - | - | - | No | No | No | - | - | No |
| Amphetamine Aspartate | - | - | - | No | No | No | - | - | No |
| Fluticasone | No | No | No | No | No | - | No | No | Yes |
| Tamsulosin | No | No | No | - | - | - | Yes | No | Yes |
| Fluoxetine | Yes | No | Yes | - | Yes | Yes | Yes | No | Yes |
| Carvedilol | Yes | No | No | No | - | No | Yes | - | Yes |
| Duloxetine | Yes | No | Yes | No | - | Yes | Yes | - | Yes |
| Meloxicam | No | No | No | No | Yes | No | No | No | - |
| Clopidogrel | - | No | Yes | Yes | Yes | Yes | Yes | No | Yes |
| Prednisone | No | No | No | No | No | No | No | No | - |
| Citalopram | No | No | - | - | - | Yes | Yes | No | Yes |
| Insulin Glargine | No | No | No | No | No | No | No | No | - |
| Potassium Chloride | - | - | - | - | - | - | - | - | - |
| Pravastatin | No | No | No | No | No | No | No | No | - |
| Tramadol | - | No | Yes | No | Yes | Yes | Yes | No | Yes |
| Aspirin | No | No | No | No | No | No | No | No | No |
| Alprazolam | - | No | Yes | - | - | - | - | No | Yes |
| Ibuprofen | No | No | - | - | No | - | No | No | No |
| Cyclobenzaprine | Yes | No | Yes | Yes | - | Yes | - | No | Yes |
| Amoxicillin | No | No | No | No | No | No | No | No | No |
| Methylphenidate | No | No | - | No | - | - | - | No | - |
| Allopurinol | - | No | - | No | No | No | No | No | No |
| Venlafaxine | No | No | - | - | Yes | Yes | Yes | No | Yes |
| Clonazepam | Yes | - | - | Yes | - | - | No | No | Yes |
| Ethinyl Estradiol | - | No | - | Yes | Yes | Yes | - | No | Yes |
| Ergocalciferol | No | No | No | No | No | No | No | No | No |
| Zolpidem | - | No | - | - | Yes | Yes | Yes | No | Yes |
| Apixaban | - | No | No | No | - | - | - | No | - |
| Glipizide | No | No | No | No | Yes | No | No | No | - |
| Spironolactone | No | No | No | No | No | No | No | No | - |
| Cetirizine | - | No | - | Yes | Yes | - | - | No | Yes |
| Atenolol | No | No | No | No | No | - | - | No | - |
| Oxycodone | - | No | - | Yes | No | - | - | No | Yes |
| Buspirone | No | No | No | No | No | No | - | No | Yes |
| Salmeterol | No | No | No | No | No | No | No | No | Yes |
| Topiramate | No | No | No | No | No | No | No | No | No |
| Warfarin | - | No | No | Yes | - | Yes | - | No | - |
| Estradiol | - | No | - | Yes | - | Yes | - | No | Yes |
| Cholecalciferol | No | No | No | No | No | No | No | No | No |
| Budesonide | No | No | No | No | No | No | No | No | Yes |
| Formoterol | No | No | - | - | - | - | - | No | - |
| Lamotrigine | Yes | No | - | - | - | - | No | No | - |
| Norgestimate | No | No | No | No | No | No | No | No | - |
| Quetlapine | No | No | No | No | - | - | Yes | No | Yes |
| Lorazepam | - | No | Yes | - | Yes | - | - | No | - |
| Famotidine | No | No | No | No | No | No | No | No | No |
| Folic Acid | No | No | No | No | No | No | No | No | No |
| Azithromycin | No | No | No | No | No | No | No | No | Yes |
| Hydroxyzine | - | No | - | Yes | - | - | Yes | No | Yes |
| Insulin Lispro | No | No | No | No | No | No | No | No | - |
| Diclofenac | - | No | Yes | Yes | Yes | Yes | - | No | Yes |
| Loratadine | Yes | No | Yes | Yes | Yes | Yes | Yes | No | Yes |
| Sitagliptin | - | No | No | No | - | No | - | No | - |
| Clonidine | Yes | - | Yes | - | - | - | - | No | Yes |
| Diltiazem | - | No | No | - | Yes | - | Yes | No | Yes |
| Latanoprost | No | No | No | No | No | No | No | No | - |
| Pregabalin | No | No | No | No | No | No | No | No | No |
| Doxycycline | No | No | No | No | No | No | No | No | No |
| Insulin Aspart | No | No | No | No | No | No | No | No | - |
| Amitriptyline | Yes | No | Yes | Yes | Yes | Yes | Yes | No | Yes |
| Paroxetine | - | No | - | No | - | - | Yes | No | - |
| Ondansetron | Yes | No | - | No | - | - | Yes | No | Yes |
| Tizanidine | Yes | - | - | - | No | - | - | No | Yes |
| Lisdexamfetamine | No | No | No | No | No | No | No | No | No |
| Rivaroxaban | No | No | No | - | - | - | - | No | Yes |
| Glimepiride | No | No | No | No | Yes | No | No | No | - |
| Propranolol | Yes | No | - | No | - | Yes | Yes | No | Yes |
| Aripiprazole | - | No | - | Yes | - | - | - | No | Yes |
| Finasteride | No | No | No | No | No | No | No | No | Yes |
| Naproxen | Yes | - | - | Yes | Yes | - | - | No | No |
| Levetiracetam | No | No | No | No | No | No | No | No | No |
| Alendronate | No | No | No | No | No | No | No | No | No |
| Fenofibrate | - | No | - | Yes | - | - | - | No | Yes |
| Oxybutynin | No | No | No | No | No | No | No | No | Yes |
| Celecoxib | - | No | No | No | Yes | Yes | Yes | No | - |
| Lovastatin | No | No | No | No | No | No | No | No | Yes |
| Ezetimibe | No | No | - | - | - | - | - | No | Yes |

Yes (orange): substrate; No (green): non-substrate

**Table S 7. CYPlebrity Testing Results** (Inhibitors)

| **Drugs** | **1A2** | **2C9** | **2C19** | **2D6** | **3A4** |
| --- | --- | --- | --- | --- | --- |
| Atorvastatin | 0.14 | 0.54 | 0.23 | 0.42 | 0.6 |
| Levothyroxin | 0.63 | 0.22 | 0.08 | 0.1 | 0.11 |
| Metformin | 0.11 | 0.05 | 0.06 | 0.15 | 0.05 |
| Lisinopril | 0.08 | 0.13 | 0.12 | 0.17 | 0.12 |
| Amlodipine | 0.55 | 0.71 | 0.71 | 0.27 | 0.56 |
| Metoprolol | 0.28 | 0.2 | 0.15 | 0.69 | 0.12 |
| Albuterol | 0.14 | 0.12 | 0.09 | 0.34 | 0.22 |
| Omeprazole | 0.73 | 0.34 | 0.8 | 0.49 | 0.56 |
| Losartan | 0.15 | 0.91 | 0.19 | 0.13 | 0.5 |
| Gabapentin | 0.04 | 0.05 | 0.05 | 0.05 | 0.05 |
| Hydrochlorothiazide | 0.1 | 0.13 | 0.1 | 0.12 | 0.07 |
| Sertraline | 0.58 | 0.44 | 0.71 | 0.83 | 0.52 |
| Simvastatin | 0.15 | 0.28 | 0.07 | 0.19 | 0.67 |
| Montelukast | 0.45 | 0.83 | 0.6 | 0.37 | 0.54 |
| Escitalopram | 0.7 | 0.68 | 0.67 | 0.66 | 0.41 |
| Acetaminophen | 0.3 | 0.17 | 0.21 | 0.1 | 0.24 |
| Hydrocodone | 0.34 | 0.2 | 0.31 | 0.39 | 0.36 |
| Rosuvastatin | 0.3 | 0.41 | 0.44 | 0.21 | 0.36 |
| Bupropion | 0.29 | 0.55 | 0.81 | 0.46 | 0.17 |
| Furosemide | 0.1 | 0.2 | 0.16 | 0.09 | 0.13 |
| Pantoprazole | 0.57 | 0.34 | 0.63 | 0.39 | 0.52 |
| Trazodone | 0.25 | 0.21 | 0.24 | 0.64 | 0.41 |
| Dextroamphetamine | 0.38 | 0.23 | 0.27 | 0.79 | 0.15 |
| Dextroamphetamine Saccharate | 0.06 | 0.07 | 0.06 | 0.1 | 0.04 |
| Amphetamine Aspartate | 0.38 | 0.23 | 0.27 | 0.79 | 0.15 |
| Fluticasone | 0.05 | 0.09 | 0.07 | 0.1 | 0.45 |
| Tamsulosin | 0.4 | 0.46 | 0.52 | 0.48 | 0.46 |
| Fluoxetine | 0.69 | 0.61 | 0.68 | 0.93 | 0.57 |
| Carvedilol | 0.21 | 0.19 | 0.17 | 0.35 | 0.44 |
| Duloxetine | 0.87 | 0.4 | 0.87 | 0.89 | 0.76 |
| Meloxicam | 0.43 | 0.47 | 0.16 | 0.11 | 0.17 |
| Clopidogrel | 0.53 | 0.57 | 0.7 | 0.52 | 0.53 |
| Prednisone | 0.04 | 0.08 | 0.07 | 0.07 | 0.1 |
| Citalopram | 0.7 | 0.68 | 0.67 | 0.66 | 0.41 |
| Insulin Glargine | - | - | - | - | - |
| Potassium Chloride | - | - | - | - | - |
| Pravastatin | 0.09 | 0.23 | 0.1 | 0.41 | 0.42 |
| Tramadol | 0.15 | 0.16 | 0.17 | 0.27 | 0.21 |
| Aspirin | 0.27 | 0.24 | 0.11 | 0.05 | 0.05 |
| Alprazolam | 0.17 | 0.18 | 0.23 | 0.19 | 0.23 |
| Ibuprofen | 0.11 | 0.2 | 0.1 | 0.14 | 0.11 |
| Cyclobenzaprine | 0.73 | 0.11 | 0.11 | 0.87 | 0.13 |
| Amoxicillin | 0.07 | 0.08 | 0.05 | 0.1 | 0.08 |
| Methylphenidate | 0.31 | 0.23 | 0.35 | 0.76 | 0.18 |
| Allopurinol | 0.28 | 0.1 | 0.08 | 0.08 | 0.08 |
| Venlafaxine | 0.52 | 0.38 | 0.42 | 0.62 | 0.45 |
| Clonazepam | 0.18 | 0.23 | 0.24 | 0.13 | 0.14 |
| Ethinyl Estradiol | 0.12 | 0.18 | 0.18 | 0.1 | 0.31 |
| Ergocalciferol | 0.08 | 0.22 | 0.31 | 0.15 | 0.25 |
| Zolpidem | 0.76 | 0.81 | 0.52 | 0.72 | 0.71 |
| Apixaban | 0.43 | 0.37 | 0.42 | 0.18 | 0.4 |
| Glipizide | 0.06 | 0.52 | 0.18 | 0.13 | 0.39 |
| Spironolactone | 0.08 | 0.15 | 0.34 | 0.1 | 0.31 |
| Cetirizine | 0.12 | 0.14 | 0.26 | 0.47 | 0.16 |
| Atenolol | 0.23 | 0.18 | 0.09 | 0.38 | 0.08 |
| Oxycodone | 0.23 | 0.17 | 0.24 | 0.56 | 0.32 |
| Buspirone | 0.15 | 0.07 | 0.13 | 0.22 | 0.2 |
| Salmeterol | 0.69 | 0.18 | 0.14 | 0.92 | 0.51 |
| Topiramate | 0.19 | 0.22 | 0.78 | 0.15 | 0.12 |
| Warfarin | 0.16 | 0.94 | 0.25 | 0.1 | 0.11 |
| Estradiol | 0.22 | 0.09 | 0.09 | 0.1 | 0.15 |
| Cholecalciferol | 0.06 | 0.2 | 0.31 | 0.21 | 0.15 |
| Budesonide | 0.05 | 0.07 | 0.08 | 0.09 | 0.21 |
| Formoterol | 0.33 | 0.16 | 0.75 | 0.88 | 0.4 |
| Lamotrigine | 0.26 | 0.19 | 0.24 | 0.26 | 0.13 |
| Norgestimate | 0.23 | 0.28 | 0.39 | 0.21 | 0.73 |
| Quetlapine | 0.21 | 0.18 | 0.15 | 0.56 | 0.17 |
| Lorazepam | 0.3 | 0.24 | 0.27 | 0.27 | 0.14 |
| Famotidine | 0.17 | 0.15 | 0.14 | 0.29 | 0.44 |
| Folic Acid | 0.05 | 0.09 | 0.07 | 0.07 | 0.07 |
| Azithromycin | 0.05 | 0.05 | 0.04 | 0.09 | 0.78 |
| Hydroxyzine | 0.14 | 0.12 | 0.19 | 0.9 | 0.19 |
| Insulin Lispro | - | - | - | - | - |
| Diclofenac | 0.21 | 0.58 | 0.15 | 0.08 | 0.18 |
| Loratadine | 0.15 | 0.47 | 0.82 | 0.26 | 0.36 |
| Sitagliptin | 0.31 | 0.32 | 0.42 | 0.48 | 0.57 |
| Clonidine | 0.26 | 0.25 | 0.28 | 0.52 | 0.13 |
| Diltiazem | 0.42 | 0.42 | 0.46 | 0.43 | 0.78 |
| Latanoprost | 0.31 | 0.34 | 0.39 | 0.37 | 0.37 |
| Pregabalin | 0.1 | 0.06 | 0.08 | 0.12 | 0.07 |
| Doxycycline | 0.07 | 0.1 | 0.07 | 0.1 | 0.24 |
| Insulin Aspart | - | - | - | - | - |
| Amitriptyline | 0.65 | 0.14 | 0.32 | 0.78 | 0.31 |
| Paroxetine | 0.56 | 0.6 | 0.69 | 0.75 | 0.78 |
| Ondansetron | 0.57 | 0.38 | 0.44 | 0.35 | 0.16 |
| Tizanidine | 0.41 | 0.3 | 0.38 | 0.44 | 0.22 |
| Lisdexamfetamine | 0.3 | 0.33 | 0.36 | 0.41 | 0.31 |
| Rivaroxaban | 0.41 | 0.29 | 0.3 | 0.45 | 0.59 |
| Glimepiride | 0.06 | 0.45 | 0.15 | 0.15 | 0.17 |
| Propranolol | 0.77 | 0.24 | 0.43 | 0.72 | 0.18 |
| Aripiprazole | 0.4 | 0.44 | 0.54 | 0.45 | 0.45 |
| Finasteride | 0.1 | 0.78 | 0.79 | 0.13 | 0.36 |
| Naproxen | 0.28 | 0.19 | 0.13 | 0.13 | 0.13 |
| Levetiracetam | 0.2 | 0.18 | 0.18 | 0.16 | 0.15 |
| Alendronate | 0.06 | 0.05 | 0.05 | 0.06 | 0.05 |
| Fenofibrate | 0.5 | 0.58 | 0.55 | 0.19 | 0.27 |
| Oxybutynin | 0.23 | 0.31 | 0.73 | 0.43 | 0.34 |
| Celecoxib | 0.42 | 0.27 | 0.59 | 0.71 | 0.15 |
| Lovastatin | 0.22 | 0.12 | 0.06 | 0.15 | 0.75 |
| Ezetimibe | 0.33 | 0.42 | 0.41 | 0.38 | 0.34 |

≥0.5(orange): inhibitors; <0.5 (green): non-inhibitors

**Table S 8. CypReact Testing Results** (Substrates)

| **Drugs** | **1A2** | **2B6** | **2A6** | **2C8** | **2C9** | **2C19** | **2D6** | **2E1** | **3A4** |
| --- | --- | --- | --- | --- | --- | --- | --- | --- | --- |
| Atorvastatin | No | No | No | Yes | Yes | No | No | No | Yes |
| Levothyroxine | No | No | No | No | Yes | No | No | No | Yes |
| Metformin | No | No | No | No | No | No | No | No | No |
| Lisinopril | No | No | No | No | No | No | No | No | Yes |
| Amlodipine | No | Yes | No | Yes | Yes | Yes | Yes | No | Yes |
| Metoprolol | No | No | No | No | No | Yes | Yes | No | Yes |
| Albuterol | No | No | No | No | No | No | Yes | No | Yes |
| Omeprazole | Yes | No | Yes | Yes | Yes | Yes | Yes | No | Yes |
| Losartan | No | No | No | No | Yes | No | Yes | No | Yes |
| Gabapentin | No | No | No | No | No | No | No | No | No |
| Hydrochlorothiazide | Yes | Yes | No | Yes | Yes | Yes | Yes | No | Yes |
| Sertraline | Yes | Yes | Yes | Yes | Yes | Yes | Yes | Yes | Yes |
| Simvastatin | No | No | No | No | No | No | No | No | Yes |
| Montelukast | Yes | Yes | Yes | No | Yes | No | Yes | No | Yes |
| Escitalopram | No | Yes | No | No | Yes | Yes | Yes | No | Yes |
| Acetaminophen | Yes | No | Yes | Yes | Yes | No | Yes | Yes | Yes |
| Hydrocodone | No | No | No | No | Yes | No | Yes | No | Yes |
| Rosuvastatin | No | No | No | Yes | Yes | No | Yes | No | Yes |
| Bupropion | No | Yes | No | No | Yes | No | Yes | Yes | Yes |
| Furosemide | Yes | No | No | Yes | Yes | Yes | No | No | Yes |
| Pantoprazole | Yes | No | No | No | Yes | Yes | Yes | No | Yes |
| Trazodone | Yes | No | No | No | Yes | No | Yes | No | Yes |
| Dextroamphetamine | Yes | No | No | No | No | No | Yes | Yes | No |
| Dextroamphetamine Saccharate | No | No | No | No | No | No | No | No | No |
| Amphetamine Aspartate | No | No | No | No | No | No | No | No | No |
| Fluticasone | No | No | No | No | No | Yes | No | No | Yes |
| Tamsulosin | No | No | No | No | Yes | No | Yes | No | Yes |
| Fluoxetine | Yes | Yes | No | No | Yes | Yes | Yes | No | Yes |
| Carvedilol | Yes | No | No | No | Yes | No | Yes | Yes | Yes |
| Duloxetine | Yes | Yes | No | No | Yes | Yes | Yes | Yes | Yes |
| Meloxicam | No | No | No | No | Yes | No | No | No | Yes |
| Clopidogrel | Yes | Yes | No | Yes | Yes | Yes | Yes | No | Yes |
| Prednisone | No | No | No | No | No | No | No | No | Yes |
| Citalopram | No | Yes | No | No | Yes | Yes | Yes | No | Yes |
| Insulin Glargine | No | No | No | No | No | No | No | No | Yes |
| Potassium Chloride | No | Yes | Yes | No | No | No | No | Yes | No |
| Pravastatin | No | No | No | Yes | No | No | No | No | Yes |
| Tramadol | No | Yes | No | No | Yes | Yes | Yes | No | Yes |
| Aspirin | No | No | Yes | No | Yes | Yes | No | No | Yes |
| Alprazolam | No | No | No | No | Yes | No | No | No | Yes |
| Ibuprofen | No | No | No | Yes | Yes | No | No | No | No |
| Cyclobenzaprine | Yes | Yes | No | Yes | Yes | Yes | Yes | No | Yes |
| Amoxicillin | No | No | No | No | No | No | No | No | Yes |
| Methylphenidate | Yes | Yes | No | No | Yes | Yes | Yes | No | Yes |
| Allopurinol | Yes | No | Yes | No | No | No | No | Yes | No |
| Venlafaxine | No | No | No | No | Yes | Yes | Yes | No | Yes |
| Clonazepam | Yes | Yes | Yes | Yes | Yes | Yes | Yes | No | Yes |
| Ethinyl Estradiol | Yes | No | No | Yes | Yes | Yes | No | No | Yes |
| Ergocalciferol | No | No | No | No | No | No | No | No | Yes |
| Zolpidem | Yes | No | No | No | Yes | Yes | Yes | No | Yes |
| Apixaban | No | No | No | No | Yes | Yes | Yes | No | Yes |
| Glipizide | Yes | No | No | Yes | Yes | Yes | Yes | No | Yes |
| Spironolactone | No | No | No | No | No | No | No | No | Yes |
| Cetirizine | No | Yes | No | Yes | Yes | Yes | Yes | No | Yes |
| Atenolol | No | No | No | No | No | Yes | Yes | No | Yes |
| Oxycodone | Yes | Yes | Yes | Yes | Yes | Yes | Yes | No | Yes |
| Buspirone | No | No | No | No | No | No | Yes | No | Yes |
| Salmeterol | No | No | No | No | No | No | Yes | No | Yes |
| Topiramate | No | No | No | No | No | No | No | No | Yes |
| Warfarin | Yes | No | No | Yes | Yes | Yes | No | No | Yes |
| Estradiol | Yes | Yes | Yes | Yes | Yes | Yes | Yes | Yes | Yes |
| Cholecalciferol | No | No | No | No | No | No | No | No | Yes |
| Budesonide | No | No | No | No | No | No | No | No | Yes |
| Formoterol | Yes | No | No | No | Yes | Yes | Yes | No | Yes |
| Lamotrigine | Yes | No | No | No | Yes | Yes | No | No | Yes |
| Norgestimate | No | No | No | No | No | No | No | No | Yes |
| Quetlapine | No | No | No | No | Yes | No | Yes | No | Yes |
| Lorazepam | Yes | Yes | No | Yes | Yes | Yes | Yes | No | Yes |
| Famotidine | Yes | No | No | No | No | Yes | No | No | Yes |
| Folic Acid | No | No | No | No | No | No | No | No | Yes |
| Azithromycin | No | No | No | No | No | No | No | No | Yes |
| Hydroxyzine | Yes | Yes | No | Yes | Yes | Yes | Yes | No | Yes |
| Insulin Lispro | No | No | No | No | No | No | No | No | Yes |
| Diclofenac | No | Yes | No | Yes | Yes | Yes | Yes | No | Yes |
| Loratadine | Yes | Yes | No | Yes | Yes | Yes | Yes | No | Yes |
| Sitagliptin | Yes | No | No | No | Yes | Yes | Yes | No | Yes |
| Clonidine | Yes | Yes | No | Yes | Yes | Yes | Yes | No | Yes |
| Diltiazem | No | No | No | Yes | Yes | No | Yes | No | Yes |
| Latanoprost | No | No | No | No | No | No | No | No | Yes |
| Pregabalin | No | No | No | No | No | No | No | No | No |
| Doxycycline | No | No | No | No | No | No | No | No | Yes |
| Insulin Aspart | No | No | No | No | No | No | No | No | Yes |
| Amitriptyline | Yes | Yes | No | Yes | Yes | Yes | Yes | No | Yes |
| Paroxetine | Yes | Yes | No | Yes | Yes | Yes | Yes | No | Yes |
| Ondansetron | Yes | No | No | No | Yes | Yes | Yes | No | Yes |
| Tizanidine | Yes | No | No | Yes | Yes | Yes | Yes | No | Yes |
| Lisdexamfetamine | Yes | No | No | No | No | Yes | Yes | No | Yes |
| Rivaroxaban | No | No | No | Yes | Yes | Yes | Yes | No | Yes |
| Glimepiride | No | No | No | Yes | Yes | No | No | No | Yes |
| Propranolol | Yes | No | No | No | Yes | Yes | Yes | No | Yes |
| Aripiprazole | No | No | No | Yes | Yes | Yes | Yes | No | Yes |
| Finasteride | No | No | No | No | No | No | No | No | Yes |
| Naproxen | Yes | No | No | Yes | Yes | Yes | No | No | No |
| Levetiracetam | No | No | No | No | No | No | No | No | Yes |
| Alendronate | No | No | No | No | No | No | No | No | No |
| Fenofibrate | Yes | Yes | No | Yes | Yes | Yes | No | No | Yes |
| Oxybutynin | No | No | No | No | Yes | No | Yes | No | Yes |
| Celecoxib | Yes | No | No | No | Yes | Yes | Yes | No | Yes |
| Lovastatin | No | No | No | No | No | No | No | No | Yes |
| Ezetimibe | Yes | No | No | Yes | Yes | Yes | Yes | No | Yes |

Yes (orange): substrate; No (green): non-substrate

**Table S 9. admetSAR Testing Results** (Inhibitors and Substrates)

| **Drugs** | **1A2 - ihn** | **2C8 - ihn** | **2C9 - ihn** | **2C19 -ihn** | **2D6 -ihn** | **3A4 -ihn** | **2C9 -sub** | **2D6 -sub** | **3A4 -sub** |
| --- | --- | --- | --- | --- | --- | --- | --- | --- | --- |
| Atorvastatin | n-i | n-i | n-i | n-i | n-i | n-i | s | n-s | s |
| Levothyroxine | n-i | i | n-i | n-i | n-i | n-i | n-s | n-s | n-s |
| Metformin | n-i | n-i | n-i | n-i | n-i | n-i | n-s | n-s | n-s |
| Lisinopril | n-i | n-i | n-i | n-i | n-i | n-i | n-s | n-s | s |
| Amlodipine | i | i | i | i | n-i | i | n-s | n-s | s |
| Metoprolol | n-i | n-i | n-i | n-i | n-i | n-i | n-s | s | n-s |
| Albuterol | n-i | n-i | n-i | n-i | n-i | n-i | n-s | s | n-s |
| Omeprazole | i | n-i | n-i | i | n-i | i | n-s | n-s | s |
| Losartan | i | i | n-i | i | n-i | i | n-s | n-s | s |
| Gabapentin | n-i | n-i | n-i | n-i | n-i | n-i | n-s | n-s | n-s |
| Hydrochlorothiazide | n-i | n-i | n-i | n-i | n-i | n-i | n-s | n-s | n-s |
| Sertraline | i | i | n-i | i | n-i | n-i | n-s | s | s |
| Simvastatin | n-i | i | n-i | n-i | n-i | i | n-s | n-s | s |
| Montelukast | n-i | i | n-i | n-i | n-i | n-i | s | n-s | s |
| Escitalopram | n-i | n-i | i | i | n-i | n-i | n-s | s | s |
| Acetaminophen | n-i | n-i | n-i | n-i | n-i | n-i | s | n-s | n-s |
| Hydrocodone | n-i | n-i | n-i | n-i | i | n-i | n-s | s | s |
| Rosuvastatin | n-i | n-i | n-i | n-i | n-i | n-i | n-s | n-s | s |
| Bupropion | n-i | n-i | n-i | i | i | n-i | n-s | n-s | s |
| Furosemide | n-i | n-i | n-i | n-i | n-i | n-i | n-s | n-s | n-s |
| Pantoprazole | i | n-i | n-i | i | n-i | n-i | n-s | n-s | s |
| Trazodone | i | n-i | n-i | i | n-i | n-i | n-s | n-s | s |
| Dextroamphetamine | n-i | n-i | n-i | n-i | n-i | n-i | s | s | s |
| Dextroamphetamine Saccharate | n-i | n-i | n-i | n-i | i | n-i | n-s | s | n-s |
| Amphetamine Aspartate | n-i | n-i | n-i | n-i | n-i | n-i | n-s | n-s | n-s |
| Fluticasone | n-i | n-i | n-i | n-i | n-i | n-i | n-s | n-s | n-s |
| Tamsulosin | n-i | n-i | n-i | n-i | n-i | i | n-s | n-s | s |
| Fluoxetine | n-i | i | i | n-i | n-i | i | n-s | s | s |
| Carvedilol | i | n-i | n-i | i | i | i | s | s | s |
| Duloxetine | i | i | n-i | n-i | n-i | n-i | s | s | s |
| Meloxicam | n-i | n-i | i | n-i | n-i | n-i | s | n-s | s |
| Clopidogrel | i | i | n-i | i | i | n-i | n-s | s | s |
| Prednisone | n-i | i | n-i | n-i | n-i | n-i | n-s | n-s | s |
| Citalopram | n-i | n-i | i | i | n-i | n-i | n-s | s | s |
| Insulin Glargine | n-i | i | n-i | n-i | n-i | n-i | n-s | n-s | s |
| Potassium Chloride | n-i | n-i | n-i | n-i | n-i | n-i | n-s | n-s | n-s |
| Pravastatin | n-i | n-i | n-i | n-i | n-i | i | n-s | n-s | s |
| Tramadol | n-i | n-i | n-i | n-i | i | n-i | n-s | s | s |
| Aspirin | n-i | n-i | n-i | n-i | n-i | n-i | s | n-s | n-s |
| Alprazolam | i | n-i | i | i | n-i | n-i | n-s | n-s | s |
| Ibuprofen | n-i | n-i | n-i | n-i | n-i | n-i | s | n-s | n-s |
| Cyclobenzaprine | i | n-i | n-i | n-i | i | i | n-s | s | s |
| Amoxicillin | n-i | n-i | n-i | n-i | n-i | n-i | n-s | n-s | s |
| Methylphenidate | n-i | n-i | n-i | n-i | n-i | n-i | n-s | s | s |
| Allopurinol | n-i | n-i | n-i | n-i | n-i | n-i | n-s | n-s | n-s |
| Venlafaxine | n-i | n-i | n-i | n-i | i | n-i | n-s | s | s |
| Clonazepam | i | n-i | i | i | n-i | i | n-s | n-s | s |
| Ethinyl Estradiol | i | i | n-i | n-i | n-i | i | s | n-s | s |
| Ergocalciferol | n-i | n-i | n-i | n-i | n-i | n-i | n-s | n-s | s |
| Zolpidem | i | n-i | n-i | n-i | n-i | n-i | s | n-s | s |
| Apixaban | n-i | n-i | i | n-i | n-i | i | n-s | n-s | s |
| Glipizide | n-i | n-i | i | n-i | n-i | n-i | s | n-s | s |
| Hydrochlorothiatide | n-i | n-i | n-i | n-i | n-i | n-i | n-s | n-s | n-s |
| Spironolactone | n-i | i | n-i | n-i | n-i | n-i | n-s | n-s | s |
| Cetirizine | n-i | n-i | n-i | n-i | n-i | n-i | n-s | n-s | n-s |
| Atenolol | n-i | n-i | n-i | n-i | n-i | n-i | n-s | s | n-s |
| Oxycodone | n-i | n-i | n-i | n-i | n-i | n-i | n-s | s | s |
| Buspirone | n-i | n-i | i | n-i | i | n-i | n-s | s | s |
| Salmeterol | i | i | n-i | n-i | i | n-i | n-s | s | s |
| Topiramate | n-i | n-i | n-i | n-i | n-i | n-i | n-s | n-s | n-s |
| Warfarin | n-i | n-i | i | n-i | n-i | n-i | s | n-s | s |
| Estradiol | i | i | n-i | i | n-i | n-i | s | s | s |
| Cholecalciferol | n-i | n-i | n-i | n-i | n-i | n-i | n-s | n-s | s |
| Budesonide | n-i | n-i | n-i | n-i | n-i | i | n-s | n-s | s |
| Formoterol | n-i | n-i | n-i | i | i | n-i | n-s | s | n-s |
| Lamotrigine | n-i | n-i | n-i | n-i | i | n-i | n-s | n-s | n-s |
| Norgestimate | n-i | n-i | n-i | n-i | n-i | n-i | n-s | n-s | s |
| Quetlapine | i | n-i | n-i | n-i | i | i | n-s | s | s |
| Lorazepam | i | n-i | n-i | i | n-i | n-i | n-s | n-s | s |
| Famotidine | n-i | n-i | n-i | n-i | n-i | n-i | n-s | n-s | n-s |
| Folic Acid | n-i | n-i | n-i | n-i | n-i | n-i | n-s | n-s | s |
| Azithromycin | n-i | n-i | n-i | n-i | n-i | n-i | n-s | n-s | s |
| Hydroxyzine | n-i | n-i | n-i | n-i | i | n-i | n-s | s | n-s |
| Insulin Lispro | n-i | i | n-i | n-i | n-i | i | n-s | n-s | s |
| Diclofenac | i | n-i | i | n-i | n-i | n-i | s | n-s | n-s |
| Loratadine | n-i | i | i | i | n-i | n-i | n-s | n-s | s |
| Sitagliptin | n-i | n-i | i | i | n-i | i | n-s | n-s | s |
| Clonidine | n-i | n-i | n-i | n-i | i | n-i | n-s | n-s | n-s |
| Diltiazem | n-i | n-i | n-i | n-i | n-i | i | s | s | s |
| Latanoprost | n-i | n-i | n-i | n-i | n-i | n-i | n-s | n-s | s |
| Pregabalin | n-i | n-i | n-i | n-i | n-i | n-i | s | n-s | n-s |
| Doxycycline | n-i | n-i | n-i | n-i | n-i | n-i | n-s | n-s | s |
| Insulin Aspart | n-i | i | n-i | n-i | n-i | n-i | n-s | n-s | s |
| Amitriptyline | i | n-i | n-i | n-i | i | n-i | s | s | s |
| Paroxetine | i | n-i | i | n-i | n-i | i | n-s | s | n-s |
| Ondansetron | i | n-i | n-i | n-i | i | n-i | s | n-s | s |
| Tizanidine | n-i | n-i | n-i | n-i | n-i | n-i | n-s | n-s | s |
| Lisdexamfetamine | n-i | n-i | n-i | n-i | n-i | n-i | n-s | n-s | n-s |
| Rivaroxaban | n-i | n-i | n-i | i | n-i | n-i | n-s | n-s | s |
| Glimepiride | n-i | n-i | i | n-i | n-i | n-i | s | n-s | s |
| Propranolol | i | n-i | n-i | n-i | i | n-i | n-s | s | n-s |
| Aripiprazole | i | n-i | i | i | i | i | n-s | s | s |
| Finasteride | n-i | n-i | i | i | n-i | n-i | n-s | n-s | s |
| Naproxen | i | n-i | n-i | n-i | n-i | n-i | s | n-s | n-s |
| Levetiracetam | n-i | n-i | n-i | n-i | n-i | n-i | n-s | n-s | n-s |
| Alendronate | n-i | n-i | n-i | n-i | n-i | n-i | s | n-s | n-s |
| Fenofibrate | i | i | n-i | i | n-i | n-i | n-s | n-s | s |
| Oxybutynin | i | i | i | i | i | i | n-s | s | s |
| Celecoxib | i | i | i | i | n-i | i | s | n-s | s |
| Lovastatin | n-i | i | n-i | n-i | n-i | i | n-s | n-s | s |
| Ezetimibe | n-i | i | i | n-i | n-i | i | n-s | n-s | s |

i (orange): inhibitor; n-i (green): non-inhibitor; s (orange): substrate; n-s (green): non-substrate

**Table S 10. admetLab 2.0 Testing Results** (Inhibitors and Substrates)

| **Drugs** | **1A2-inh** | **2C19-inh** | **2C9-inh** | **2D6-inh** | **3A4-inh** | **1A2-sub** | **2C19-sub** | **2C9-sub** | **2D6-sub** | **3A4-sub** |
| --- | --- | --- | --- | --- | --- | --- | --- | --- | --- | --- |
| Atorvastatin | n-i (0.311) | n-i (0.428) | i (0.857) | n-i (0.074) | n-i (0.075) | n-s (0.099) | n-s (0.095) | s (0.961) | n-s (0.299) | s (0.729) |
| Levothyroxine | n-i (0.383) | n-i (0.027) | n-i (0.345) | n-i (0.108) | n-i (0.059) | n-s (0.112) | n-s (0.065) | n-s (0.099) | n-s (0.219) | n-s (0.267) |
| Metformin | n-i (0.015) | n-i (0.015) | n-i (0.002) | n-i (0.013) | n-i (0.001) | n-s (0.071) | n-s (0.066) | n-s (0.029) | s (0.858) | n-s (0.077) |
| Lisinopril | n-i (0.013) | n-i (0.036) | n-i (0.013) | n-i (0.013) | n-i (0.013) | n-s (0.032) | n-s (0.049) | n-s (0.325) | n-s (0.219) | n-s (0.011) |
| Amlodipine | n-i (0.438) | i (0.523) | n-i (0.21) | n-i (0.017) | i (0.896) | n-s (0.248) | s (0.832) | n-s (0.029) | n-s (0.15) | s (0.857) |
| Metoprolol | n-i (0.137) | n-i (0.056 | n-i (0.01) | n-i (0.353) | n-i (0.012) | n-s (0.105) | s (0.912) | n-s (0.078) | s (0.889) | s (0.765) |
| Albuterol | n-i (0.02 | n-i (0.021 | n-i (0.001 | n-i (0.17 | n-i (0.005 | n-s (0.1) | s (0.761) | n-s (0.469) | s (0.812) | s (0.693) |
| Omeprazole | i  (0.92) | n-i (0.497) | n-i (0.443) | n-i (0.166) | i (0.879) | s (0.953) | s (0.813) | s (0.864) | s (0.896) | s (0.874) |
| Losartan | i  (0.795) | i  (0.968) | i  (0.945) | i  (0.698) | i  (0.919) | n-s (0.094) | n-s (0.06) | s (0.946) | n-s (0.025) | n-s (0.435) |
| Gabapentin | n-i (0.024) | n-i (0.031) | n-i (0.027) | n-i (0.06) | n-i (0.026) | n-s (0.115) | n-s (0.098) | n-s (0.498) | n-s (0.48) | n-s (0.052 |
| Hydrochlorothiazide | n-i (0.03) | n-i (0.022) | n-i (0.015) | n-i (0.006) | n-i (0.033) | s  (0.7) | n-s (0.117) | n-s (0.102) | n-s (0.107) | n-s (0.077) |
| Sertraline | n-i (0.342) | i (0.914) | n-i (0.421) | i (0.944) | i (0.853) | s (0.968) | s (0.949) | s (0.869) | s (0.927) | s (0.916) |
| Simvastatin | n-i (0.013) | n-i (0.022) | n-i (0.032) | n-i (0.003) | i  (0.9) | n-s (0.06 | s (0.834) | n-s (0.329) | n-s (0.363) | s (0.718) |
| Montelukast | n-i (0.36) | i (0.633) | n-i (0.332) | n-i (0.444) | n-i (0.322) | n-s (0.481) | n-s (0.084) | s (0.943) | s (0.897) | n-s (0.387) |
| Escitalopram | n-i (0.067) | n-i (0.086) | n-i (0.095) | i (0.517) | n-i (0.206) | s  (0.87) | s (0.974) | n-s (0.169) | s (0.921) | s  (0.92) |
| Acetaminophen | n-i (0.29) | n-i (0.103) | n-i (0.024) | n-i (0.039) | n-i (0.037) | s  (0.72) | n-s  (0.26) | s  (0.856) | s  (0.67) | n-s  (0.373) |
| Hydrocodone | n-i (0.056) | n-i (0.033) | n-i (0.008) | i  (0.89) | n-i (0.022) | s  (0.505) | s  (0.965) | n-s  (0.264) | s  (0.919) | s  (0.878) |
| Rosuvastatin | n-i (0.073) | n-i (0.016) | n-i (0.013) | n-i (0.004) | n-i (0.016) | n-s  (0.174) | n-s  (0.365) | s  (0.549) | n-s  (0.117) | s  (0.915) |
| Bupropion | n-i (0.342) | n-i (0.239) | n-i (0.042) | i (0.829) | n-i (0.08) | s  (0.889) | s  (0.864) | n-s  (0.353) | s  (0.87) | s  (0.64) |
| Furosemide | n-i (0.07) | n-i (0.036) | n-i (0.339) | n-i (0.008) | n-i (0.021) | n-s (0.08) | n-s  (0.053) | n-s  (0.21) | n-s  (0.135) | n-s  (0.081) |
| Pantoprazole | i  (0.96) | n-i (0.258) | n-i (0.277) | n-i (0.098) | i (0.649) | s  (0.963) | s  (0.526) | s  (0.597) | s  (0.776) | s  (0.643) |
| Trazodone | n-i (0.216) | n-i (0.351) | n-i (0.068) | i (0.917) | n-i (0.058) | s  (0.645) | s  (0.823) | n-s  (0.179) | s  (0.87) | s  (0.903) |
| Dextroamphetamine | i (0.633) | n-i (0.043) | n-i (0.01) | i (0.921) | n-i (0.025) | n-s  (0.155) | s  (0.731) | n-s  (0.065) | s  (0.881) | n-s  (0.338) |
| Dextroamphetamine Saccharate | n-i (0.007) | n-i (0.015) | n-i (0.002) | n-i (0.002) | n-i (0.005) | n-s  (0.021) | n-s  (0.054) | n-s  (0.063) | n-s  (0.094) | n-s  (0.006) |
| Amphetamine Aspartate | i (0.633) | n-i (0.043) | n-i (0.01) | i (0.921) | n-i (0.025) | n-s  (0.155) | s  (0.731) | n-s  (0.065) | s  (0.881) | n-s  (0.338) |
| Fluticasone | n-i (0.015) | n-i (0.115) | n-i (0.082) | n-i (0.276) | i (0.926) | s  (0.578) | s  (0.795) | n-s  (0.15) | n-s  (0.055) | s  (0.925) |
| Tamsulosin | n-i (0.297) | n-i (0.179) | n-i (0.045) | i  (0.96) | i (0.666) | s  (0.811) | s  (0.917) | n-s  (0.493) | s  (0.912) | s  (0.891) |
| Fluoxetine | i (0.765) | i  (0.75) | n-i (0.265) | i (0.943) | i (0.858) | s  (0.912) | s  (0.949) | s  (0.792) | s  (0.913) | n-s  (0.492) |
| Carvedilol | i  (0.984) | i  (0.673) | n-i  (0.062) | i  (0.987) | i  (0.943) | s  (0.891) | s  (0.849) | s  (0.753) | s  (0.95) | s  (0.825) |
| Duloxetine | i  (0.91) | i  (0.762) | n-i  (0.203) | i  (0.982) | i  (0.935) | s  (0.944) | s  (0.937) | s  (0.595) | s  (0.94) | s  (0.681) |
| Meloxicam | n-i  (0.231) | i  (0.529) | i  (0.635) | n-i  (0.151) | i  (0.776) | s  (0.62) | s  (0.695) | s  (0.929) | n-s  (0.14) | s  (0.831) |
| Clopidogrel | i  (0.862) | i  (0.951) | i  (0.651) | i  (0.891) | i  (0.667) | s  (0.917) | s  (0.86) | s  (0.826) | s  (0.886) | s  (0.912) |
| Prednisone | n-i  (0.01) | n-i  (0.039) | n-i  (0.037) | n-i  (0.01) | n-i  (0.411) | s  (0.801) | s  (0.778) | n-s  (0.085) | n-s  (0.063) | s  (0.923) |
| Citalopram | n-i  (0.067) | n-i  (0.086) | n-i  (0.095) | i  (0.517) | n-i  (0.206) | s  (0.87) | s  (0.974) | n-s  (0.169) | s  (0.921) | s  (0.92) |
| Insulin Glargine | n-i  (0.000) | n-i  (0.000) | n-i  (0.000) | n-i  (0.000) | n-i  (0.000) | n-s  (0.000) | n-s  (0.000) | n-s  (0.001) | n-s  (0.000) | n-s  (0.000) |
| Potassium Chloride | - | - | - | - | - | - | - | - | - | - |
| Pravastatin | n-i  (0.005) | n-i  (0.011) | n-i  (0.002) | n-i  (0.001) | n-i  (0.045) | n-s  (0.061) | s  (0.821) | s  (0.75) | n-s  (0.109) | s  (0.679) |
| Tramadol | n-i  (0.075) | n-i  (0.035) | n-i  (0.009) | i  (0.932) | n-i  (0.026) | s  (0.901) | s  (0.977) | s  (0.614) | s  (0.937) | s  (0.797) |
| Aspirin | n-i  (0.059) | n-i  (0.03) | n-i  (0.059) | n-i  (0.015) | n-i  (0.016) | n-s  (0.057) | n-s  (0.056) | n-s  (0.143) | n-s  (0.104) | n-s  (0.114) |
| Alprazolam | i  (0.523 | n-i  (0.299) | n-i  (0.396) | n-i  (0.008) | n-i  (0.114) | s  (0.968) | s  (0.892) | n-s  (0.152) | n-s  (0.031) | s  (0.956) |
| Ibuprofen | n-i  (0.079) | n-i  (0.057) | n-i  (0.416) | n-i  (0.004) | n-i  (0.014) | n-s  (0.317) | s  (0.91) | s  (0.982) | n-s  (0.101) | n-s  (0.195) |
| Cyclobenzaprine | n-i  (0.337) | n-i  (0.039) | n-i  (0.002) | i  (0.979) | n-i  (0.046) | s  (0.933) | s  (0.985) | s  (0.588) | s  (0.966) | s  (0.872) |
| Amoxicillin | n-i  (0.004 | n-i  (0.032 | n-i  (0.057 | n-i  (0.181 | n-i  (0.034 | n-s  (0.037 | n-s  (0.06 | s  (0.751 | n-s  (0.122 | n-s  (0.045 |
| Methylphenidate | n-i  (0.151 | n-i  (0.229 | n-i  (0.014 | n-i  (0.532 | n-i  (0.301 | n-s  (0.473 | s  (0.937 | n-s  (0.174 | s  (0.783 | s  (0.601 |
| Allopurinol | n-i  (0.202 | n-i  (0.064 | n-i  (0.013 | n-i  (0.009 | n-i  (0.039 | s  (0.819 | n-s  (0.062) | s  (0.845) | n-s  (0.095) | n-s  (0.155) |
| Venlafaxine | n-i  (0.079) | n-i  (0.047) | n-i  (0.022) | i  (0.791) | n-i  (0.04) | s  (0.946) | s  (0.976) | s  (0.721) | s  (0.934) | s  (0.601) |
| Clonazepam | i  (0.706) | i  (0.714) | i  (0.76) | n-i  (0.476) | i  (0.52) | s  (0.798) | s  (0.616) | s  (0.909) | s  (0.543) | s  (0.912) |
| Ethinyl Estradiol | i  (0.808) | i  (0.952) | i  (0.797) | i  (0.681) | i  (0.749) | s  (0.94) | s  (0.887) | s  (0.923) | s  (0.764) | s  (0.932) |
| Ergocalciferol | n-i  (0.103) | n-i  (0.208) | n-i  (0.152) | n-i  (0.084) | n-i  (0.277) | s  (0.583) | s  (0.926) | n-s  (0.088) | n-s  (0.254) | s  (0.907) |
| Zolpidem | i  (0.842) | i  (0.875) | i  (0.662) | n-i  (0.032) | n-i  (0.425) | s  (0.939) | s  (0.897) | s  (0.578) | s  (0.836) | s  (0.788) |
| Apixaban | n-i  (0.291) | n-i  (0.444) | i  (0.801) | n-i  (0.154) | i  (0.719) | s  (0.763) | n-s  (0.364) | s  (0.823) | s  (0.608) | s  (0.602) |
| Glipizide | n-i  (0.021) | n-i  (0.072) | n-i  (0.439) | n-i  (0.006) | n-i  (0.168) | n-s  (0.065) | n-s  (0.333) | s  (0.96) | n-s  (0.129) | s  (0.607) |
| Hydrochlorothiatide | n-i  (0.03) | n-i  (0.022) | n-i  (0.015) | n-i  (0.006) | n-i  (0.033) | s  (0.7) | n-s  (0.117) | n-s  (0.102) | n-s  (0.107) | n-s  (0.077) |
| Spironolactone | n-i  (0.04) | n-i  (0.242) | n-i  (0.241) | n-i  (0.006) | i  (0.742) | s  (0.528) | s  (0.855) | n-s  (0.216) | n-s  (0.357) | s  (0.75) |
| Cetirizine | n-i  (0.059) | n-i  (0.246) | n-i  (0.19) | i  (0.668) | n-i  (0.061) | n-s  (0.108) | s  (0.808) | s  (0.528) | s  (0.683) | s  (0.913) |
| Atenolol | n-i  (0.048) | n-i  (0.034) | n-i  (0.01) | n-i  (0.097) | n-i  (0.014) | n-s  (0.071) | s  (0.734) | n-s  (0.129) | s  (0.793) | n-s  (0.399) |
| Oxycodone | n-i  (0.037) | n-i  (0.023) | n-i  (0.013) | n-i  (0.278) | n-i  (0.019) | n-s  (0.375) | s  (0.948) | n-s  (0.242) | s  (0.907) | s  (0.825) |
| Buspirone | n-i  (0.073) | n-i  (0.168) | n-i  (0.219) | i  (0.667) | i  (0.765) | n-s  (0.377) | s  (0.668) | n-s  (0.116) | s  (0.7) | s  (0.726) |
| Salmeterol | n-i  (0.105) | n-i  (0.092) | n-i  (0.013) | i  (0.969) | i  (0.571) | n-s  (0.205) | n-s  (0.48) | n-s  (0.056) | n-s  (0.211) | s  (0.668) |
| Topiramate | n-i  (0.001) | n-i  (0.012) | n-i  (0.004) | n-i  (0.005) | n-i  (0.005) | s  (0.518) | s  (0.85) | n-s  (0.016) | n-s  (0.088) | n-s  (0.288) |
| Warfarin | n-i  (0.452) | i  (0.909) | i  (0.92) | n-i  (0.026) | n-i  (0.229) | s  (0.941) | s  (0.89) | s  (0.966) | s  (0.6) | s  (0.836) |
| Estradiol | n-i  (0.352) | n-i  (0.196) | n-i  (0.269) | n-i  (0.077) | n-i  (0.084) | s  (0.826) | s  (0.837) | s  (0.904) | s  (0.908) | n-s  (0.407) |
| Cholecalciferol | n-i  (0.129) | n-i  (0.237) | n-i  (0.146) | n-i  (0.037) | n-i  (0.158) | n-s  (0.435) | s  (0.853) | n-s  (0.362) | n-s  (0.162) | s  (0.7) |
| Budesonide | n-i  (0.003) | n-i  (0.03) | n-i  (0.029) | n-i  (0.002) | i  (0.869) | s  (0.682) | s  (0.815) | n-s  (0.119) | n-s  (0.05) | s  (0.884) |
| Formoterol | n-i  (0.307) | n-i  (0.28) | n-i  (0.134) | i  (0.917) | n-i  (0.058) | s  (0.932) | s  (0.869) | s  (0.859) | s  (0.916) | s  (0.853) |
| Lamotrigine | n-i  (0.204) | n-i  (0.323) | n-i  (0.152) | n-i  (0.013) | n-i  (0.068) | s  (0.784) | n-s  (0.035) | n-s  (0.014) | n-s  (0.088) | n-s  (0.226) |
| Norgestimate | n-i  (0.352) | i  (0.909) | i  (0.894) | n-i  (0.317) | i  (0.909) | s  (0.811) | s  (0.78) | n-s  (0.29) | n-s  (0.159) | s  (0.915) |
| Quetlapine | i  (0.517) | n-i  (0.383) | n-i  (0.072) | i  (0.828) | n-i  (0.301) | n-s  (0.16) | s  (0.855) | s  (0.501) | s  (0.916) | s  (0.818) |
| Lorazepam | i  (0.553) | i  (0.566) | i  (0.64) | n-i  (0.342) | n-i  (0.431) | n-s  (0.325) | s  (0.604) | s  (0.951) | n-s  (0.178) | s  (0.786) |
| Famotidine | n-i  (0.009) | n-i  (0.051) | n-i  (0.045) | n-i  (0.048) | n-i  (0.014) | s  (0.804) | n-s  (0.054) | n-s  (0.082) | n-s  (0.174 | n-s  (0.252 |
| Folic Acid | n-i  (0.005) | n-i  (0.037) | n-i  (0.01) | n-i  (0) | n-i  (0.026) | n-s  (0.074) | n-s  (0.026) | n-s  (0.007) | n-s  (0.044) | n-s  (0.005) |
| Azithromycin | n-i  (0.000) | n-i  (0.002) | n-i  (0.000) | n-i  (0.003) | n-i  (0.013) | n-s  (0.057) | s  (0.984) | n-s  (0.047) | s  (0.606) | s  (0.908) |
| Hydroxyzine | n-i  (0.06) | i  (0.677) | n-i  (0.404) | i  (0.948) | i  (0.611) | n-s  (0.119) | s  (0.709) | n-s  (0.422) | n-s  (0.456) | s  (0.926) |
| Insulin Lispro | n-i  (0.000) | n-i  (0.000) | n-i  (0.004) | n-i  (0.000) | n-i  (0.009) | n-s  (0.000) | n-s  (0.001) | n-s  (0.029) | n-s  (0.000) | n-s  (0.000) |
| Diclofenac | n-i  (0.43) | n-i  (0.242) | i  (0.694) | n-i  (0.007) | n-i  (0.145) | s  (0.884) | s  (0.812) | s  (0.973) | n-s  (0.309) | n-s  (0.326) |
| Loratadine | n-i  (0.336) | i  (0.922) | i  (0.841) | i  (0.555) | i  (0.663) | s  (0.919) | s  (0.708) | s  (0.735) | s  (0.635) | s  (0.831) |
| Sitagliptin | n-i  (0.063) | n-i  (0.141) | n-i  (0.1) | n-i  (0.166) | n-i  (0.198) | n-s  (0.091) | s  (0.731) | n-s  (0.146) | s  (0.519) | n-s  (0.424) |
| Clonidine | n-i  (0.257) | n-i  (0.12) | n-i  (0.026) | i  (0.863) | n-i  (0.119) | s  (0.94) | s  (0.862) | s  (0.818) | s  (0.9) | n-s  (0.322) |
| Diltiazem | n-i  (0.092) | n-i  (0.392) | n-i  (0.087) | n-i  (0.297) | i  (0.87) | s  (0.659) | s  (0.967) | s  (0.716) | s  (0.9) | s  (0.917) |
| Latanoprost | n-i  (0.164) | n-i  (0.057) | n-i  (0.32) | n-i  (0.001) | n-i  (0.053) | n-s  (0.145) | n-s  (0.056) | s  (0.925) | n-s  (0.049) | n-s  (0.292) |
| Pregabalin | n-i  (0.04) | n-i  (0.018) | n-i  (0.008) | n-i  (0.045) | n-i  (0.015) | n-s  (0.086) | n-s  (0.272) | s  (0.875) | n-s  (0.316) | n-s  (0.097) |
| Doxycycline | n-i  (0.009) | n-i  (0.017) | n-i  (0.004) | n-i  (0.006) | n-i  (0.1) | n-s  (0.092) | n-s  (0.096) | n-s  (0.091) | n-s  (0.137) | s  (0.669) |
| Insulin Aspart | n-i  (0.000) | n-i  (0.000) | n-i  (0.000) | n-i  (0.000) | n-i  (0.000) | n-s  (0.000) | n-s  (0.000) | n-s  (0.010) | n-s  (0.000) | n-s  (0.000) |
| Amitriptyline | n-i  (0.353) | n-i  (0.076) | n-i  (0.008) | i  (0.99) | n-i  (0.084) | s  (0.881) | s  (0.983) | s  (0.578) | s  (0.953) | s  (0.885) |
| Paroxetine | i  (0.93) | i  (0.701) | n-i  (0.1) | i  (0.989) | i  (0.961) | n-s  (0.366) | s  (0.925) | n-s  (0.385) | s  (0.941) | s  (0.633) |
| Ondansetron | i  (0.93) | i  (0.767) | n-i  (0.333) | i  (0.864) | i  (0.72) | s  (0.893) | s  (0.864) | s  (0.797) | s  (0.881) | s  (0.695) |
| Tizanidine | n-i  (0.453) | n-i  (0.341) | n-i  (0.077) | i  (0.872) | n-i  (0.246) | s  (0.93) | s  (0.866) | s  (0.818 | s  (0.903) | n-s  (0.231) |
| Lisdexamfetamine | n-i  (0.1) | n-i  (0.053) | n-i  (0.015) | i  (0.517) | n-i  (0.419) | n-s  (0.078) | n-s  (0.137) | n-s  (0.063) | s  (0.712) | n-s  (0.237) |
| Rivaroxaban | n-i  (0.171) | i  (0.704) | i  (0.764) | n-i  (0.2) | i  (0.586) | n-s  (0.116) | n-s  (0.498) | n-s  (0.249) | n-s  (0.278) | s  (0.568) |
| Glimepiride | n-i  (0.029) | n-i  (0.133) | i  (0.864) | n-i  (0.055) | n-i  (0.302) | n-s  (0.121) | s  (0.632) | s  (0.968) | n-s  (0.16) | s  (0.757) |
| Propranolol | i  (0.952) | n-i  (0.105) | n-i  (0.011) | i  (0.9) | n-i  (0.034) | s  (0.545) | s  (0.911) | n-s  (0.118) | s  (0.906) | s  (0.687) |
| Aripiprazole | n-i  (0.325) | i  (0.83) | i  (0.632) | i  (0.958) | i  (0.715) | s  (0.967) | s  (0.786) | s  (0.508) | s  (0.934) | s  (0.879) |
| Finasteride | n-i  (0.043) | i  (0.776) | n-i  (0.303) | n-i  (0.129) | i  (0.907) | s  (0.757) | s  (0.823) | n-s  (0.187) | n-s  (0.455) | s  (0.82) |
| Naproxen | n-i  (0.207) | n-i  (0.087) | n-i  (0.116) | n-i  (0.025) | n-i  (0.039) | s  (0.929) | s  (0.621) | s  (0.952) | n-s  (0.314) | n-s  (0.134) |
| Levetiracetam | n-i  (0.012) | n-i  (0.027) | n-i  (0.011) | n-i  (0.007) | n-i  (0.007) | n-s  (0.124) | n-s  (0.151) | n-s  (0.422) | n-s  (0.158) | n-s  (0.155) |
| Alendronate | n-i  (0.000) | n-i  (0.025) | n-i  (0.016) | n-i  (0.014) | n-i  (0.004) | n-s  (0.066) | n-s  (0.032) | s  (0.86) | n-s  (0.099) | n-s  (0.006) |
| Fenofibrate | i  (0.777) | i  (0.776) | i  (0.713) | n-i  (0.104) | n-i  (0.097) | n-s  (0.279) | n-s  (0.066) | n-s  (0.169) | n-s  (0.032) | s  (0.76) |
| Oxybutynin | i  (0.632) | i  (0.874) | i  (0.572) | i  (0.946) | i  (0.526) | s  (0.743) | s  (0.841) | n-s  (0.175) | s  (0.529) | s  (0.761) |
| Celecoxib | i  (0.835) | i  (0.714) | i  (0.858) | n-i  (0.052) | n-i  (0.145) | s  (0.602) | s  (0.624) | s  (0.696) | s  (0.599) | s  (0.854) |
| Lovastatin | n-i  (0.017) | n-i  (0.016) | n-i  (0.018) | n-i  (0.003) | i  (0.891) | n-s  (0.059) | s  (0.796) | n-s  (0.128) | n-s  (0.122) | s  (0.765) |
| Ezetimibe | n-i  (0.192) | i  (0.627) | i  (0.554) | n-i  (0.202) | i  (0.544) | s  (0.618) | n-s  (0.189) | s  (0.96) | n-s  (0.483) | s  (0.893) |

i (≥0.5) (orange): inhibitor; n-i (<0.5) (green): non-inhibitor; s (≥0.5) (orange): substrate; n-s (<0.5) (green): non-substrate

**Table S 11. admetLab 3.0 Testing Results** (Inhibitors and Substrates)

| **Drugs** | **CYP1A2-inh** | **CYP2C19-inh** | **CYP2C9-inh** | **CYP2D6-inh** | **CYP3A4-inh** | **CYP2B6-inh** | **CYP2C8-inh** | **CYP1A2-sub** | **CYP2C19-sub** | **CYP2C9-sub** | **CYP2D6-sub** | **CYP3A4-sub** |
| --- | --- | --- | --- | --- | --- | --- | --- | --- | --- | --- | --- | --- |
| Atorvastatin | 0,000 | 0,000 | 0,026 | 0,000 | 0,000 | 0,000 | 0,000 | 0,000 | 0,002 | 0,000 | 0,000 | 0,000 |
| Levothyroxine | 0,000 | 0,000 | 1,000 | 0,000 | 0,000 | 1,000 | 1,000 | 0,000 | 1,000 | 1,000 | 1,000 | 1,000 |
| Metformin | 0,000 | 0,000 | 0,000 | 0,000 | 0,000 | 0,000 | 0,000 | 0,978 | 0,052 | 0,011 | 0,345 | 0,345 |
| Lisinopril | 0,000 | 0,000 | 0,000 | 0,000 | 0,000 | 0,000 | 0,000 | 0,000 | 0,006 | 0,999 | 0,551 | 0,551 |
| Amlodipine | 0,004 | 0,554 | 0,239 | 0,202 | 0,984 | 1,000 | 0,993 | 0,513 | 0,198 | 0,000 | 0,000 | 0,000 |
| Metoprolol | 0,000 | 0,000 | 0,000 | 0,284 | 0,000 | 0,000 | 0,000 | 1,000 | 1,000 | 0,112 | 1,000 | 1,000 |
| Albuterol | 0,000 | 0,000 | 0,000 | 0,000 | 0,008 | 0,000 | 0,000 | 0,000 | 1,000 | 0,000 | 1,000 | 1,000 |
| Omeprazole | 1,000 | 1,000 | 0,000 | 0,001 | 1,000 | 0,000 | 0,904 | 1,000 | 1,000 | 0,997 | 1,000 | 1,000 |
| Losartan | 0,010 | 0,750 | 0,026 | 0,000 | 0,004 | 0,000 | 0,709 | 0,519 | 0,721 | 1,000 | 0,000 | 0,000 |
| Gabapentin | 0,000 | 0,000 | 0,000 | 0,001 | 0,000 | 0,000 | 0,000 | 0,037 | 0,364 | 0,404 | 0,083 | 0,083 |
| Hydrochlorothiazide | 0,000 | 0,000 | 0,000 | 0,000 | 0,000 | 0,000 | 0,001 | 0,000 | 0,000 | 0,013 | 0,000 | 0,000 |
| Sertraline | 0,991 | 0,961 | 0,036 | 0,991 | 0,235 | 1,000 | 0,994 | 1,000 | 1,000 | 0,964 | 0,994 | 0,994 |
| Simvastatin | 0,000 | 0,001 | 0,005 | 0,000 | 1,000 | 0,000 | 1,000 | 0,000 | 0,362 | 0,000 | 0,000 | 0,000 |
| Montelukast | 0,244 | 0,413 | 0,005 | 0,002 | 0,670 | 1,000 | 1,000 | 1,000 | 1,000 | 1,000 | 0,161 | 0,161 |
| Escitalopram | 0,000 | 0,000 | 0,000 | 0,151 | 0,001 | 0,000 | 0,000 | 0,485 | 1,000 | 0,005 | 0,998 | 0,998 |
| Acetaminophen | 0,017 | 0,017 | 0,032 | 0,000 | 0,003 | 0,001 | 0,007 | 0,065 | 0,988 | 0,378 | 0,926 | 0,926 |
| Hydrocodone | 0,000 | 0,001 | 0,000 | 0,011 | 0,010 | 0,002 | 0,000 | 0,679 | 1,000 | 0,913 | 0,721 | 0,721 |
| Rosuvastatin | 0,000 | 0,000 | 0,337 | 0,000 | 0,000 | 0,000 | 0,000 | 0,001 | 0,887 | 1,000 | 0,000 | 0,000 |
| Bupropion | 0,061 | 0,003 | 0,300 | 0,999 | 0,002 | 0,001 | 0,000 | 0,998 | 1,000 | 0,994 | 0,931 | 0,931 |
| Furosemide | 0,000 | 0,000 | 0,000 | 0,000 | 0,000 | 0,000 | 0,012 | 0,000 | 0,000 | 0,144 | 0,000 | 0,000 |
| Pantoprazole | 0,979 | 0,984 | 0,014 | 0,000 | 0,710 | 0,000 | 0,006 | 1,000 | 0,981 | 0,000 | 0,000 | 0,000 |
| Trazodone | 0,000 | 0,000 | 0,000 | 0,001 | 0,000 | 0,000 | 0,004 | 0,997 | 0,177 | 0,468 | 0,794 | 0,794 |
| Dextroamphetamine | 0,000 | 0,000 | 0,000 | 0,729 | 0,001 | 0,000 | 0,000 | 0,977 | 0,997 | 0,000 | 1,000 | 1,000 |
| Dextroamphetamine Saccharate | 0,002 | 0,000 | 0,000 | 0,000 | 0,000 | 0,000 | 0,961 | 0,000 | 0,000 | 0,630 | 0,029 | 0,029 |
| Amphetamine Aspartate | 0,000 | 0,000 | 0,000 | 0,729 | 0,001 | 0,000 | 0,000 | 0,977 | 0,997 | 0,000 | 1,000 | 1,000 |
| Fluticasone | 0,000 | 0,008 | 0,008 | 0,000 | 0,989 | 0,000 | 0,042 | 0,032 | 0,014 | 0,178 | 0,000 | 0,000 |
| Tamsulosin | 0,953 | 0,001 | 0,000 | 0,994 | 0,027 | 0,922 | 0,986 | 0,108 | 0,344 | 0,011 | 1,000 | 1,000 |
| Fluoxetine | 0,321 | 0,957 | 0,000 | 0,986 | 0,999 | 0,876 | 0,000 | 1,000 | 1,000 | 0,999 | 1,000 | 1,000 |
| Carvedilol | 1,000 | 0,491 | 0,001 | 1,000 | 0,816 | 0,339 | 0,948 | 0,986 | 0,992 | 1,000 | 1,000 | 1,000 |
| Duloxetine | 1,000 | 1,000 | 0,008 | 1,000 | 0,095 | 1,000 | 0,000 | 1,000 | 1,000 | 0,998 | 1,000 | 1,000 |
| Meloxicam | 0,000 | 1,000 | 1,000 | 0,000 | 0,992 | 0,036 | 0,998 | 0,000 | 0,635 | 1,000 | 0,000 | 0,000 |
| Clopidogrel | 1,000 | 0,999 | 0,996 | 0,837 | 1,000 | 1,000 | 1,000 | 1,000 | 0,998 | 1,000 | 0,999 | 0,999 |
| Prednisone | 0,000 | 0,000 | 0,000 | 0,000 | 0,013 | 0,000 | 0,955 | 0,011 | 0,622 | 0,057 | 0,000 | 0,000 |
| Citalopram | 0,000 | 0,000 | 0,000 | 0,151 | 0,001 | 0,000 | 0,000 | 0,485 | 1,000 | 0,005 | 0,998 | 0,998 |
| Insulin Glargine | 0,000 | 0,000 | 0,000 | 0,000 | 0,000 | 0,000 | 0,000 | 0,000 | 0,180 | 0,000 | 0,000 | 0,000 |
| Potassium Chloride | 0,754 | 0,977 | 0,606 | 0,035 | 0,001 | 0,045 | 0,280 | 0,330 | 0,041 | 0,115 | 0,047 | 0,047 |
| Pravastatin | 0,000 | 0,000 | 0,000 | 0,000 | 0,000 | 0,000 | 0,000 | 0,005 | 0,052 | 0,044 | 0,000 | 0,000 |
| Tramadol | 0,000 | 0,000 | 0,000 | 0,209 | 0,223 | 0,000 | 0,000 | 0,688 | 1,000 | 0,905 | 1,000 | 1,000 |
| Aspirin | 0,000 | 0,000 | 0,001 | 0,000 | 0,000 | 0,000 | 0,006 | 0,000 | 0,000 | 0,182 | 0,000 | 0,000 |
| Alprazolam | 0,913 | 0,001 | 0,001 | 0,000 | 0,965 | 0,000 | 0,000 | 1,000 | 1,000 | 0,001 | 0,000 | 0,000 |
| Ibuprofen | 0,000 | 0,000 | 0,001 | 0,000 | 0,000 | 0,000 | 0,000 | 0,000 | 1,000 | 1,000 | 0,000 | 0,000 |
| Cyclobenzaprine | 0,000 | 0,000 | 0,000 | 1,000 | 0,000 | 0,000 | 0,021 | 1,000 | 1,000 | 0,000 | 0,997 | 0,997 |
| Amoxicillin | 0,000 | 0,000 | 0,000 | 0,151 | 0,000 | 0,000 | 0,000 | 0,000 | 1,000 | 0,000 | 0,009 | 0,009 |
| Methylphenidate | 0,000 | 0,000 | 0,000 | 0,002 | 0,001 | 0,000 | 0,000 | 0,989 | 0,056 | 0,004 | 0,000 | 0,000 |
| Allopurinol | 0,009 | 0,000 | 0,000 | 0,000 | 0,000 | 0,000 | 0,000 | 0,000 | 0,000 | 0,011 | 0,000 | 0,000 |
| Venlafaxine | 0,000 | 0,003 | 0,000 | 0,001 | 0,057 | 0,000 | 0,000 | 1,000 | 1,000 | 1,000 | 0,999 | 0,999 |
| Clonazepam | 0,764 | 0,096 | 0,000 | 0,000 | 0,217 | 0,000 | 0,016 | 1,000 | 0,077 | 0,000 | 0,000 | 0,000 |
| Ethinyl Estradiol | 0,601 | 0,994 | 0,701 | 0,072 | 0,455 | 0,002 | 0,994 | 0,995 | 1,000 | 0,968 | 0,000 | 0,000 |
| Ergocalciferol | 0,000 | 0,000 | 0,009 | 0,000 | 0,002 | 0,011 | 0,607 | 0,000 | 0,982 | 0,957 | 0,011 | 0,011 |
| Zolpidem | 0,000 | 0,000 | 0,014 | 0,000 | 0,004 | 0,008 | 0,000 | 0,981 | 1,000 | 0,998 | 0,947 | 0,947 |
| Apixaban | 0,000 | 0,005 | 0,007 | 0,000 | 0,691 | 0,001 | 0,996 | 1,000 | 0,004 | 0,962 | 0,029 | 0,029 |
| Glipizide | 0,000 | 0,000 | 0,272 | 0,000 | 0,001 | 0,000 | 0,001 | 0,000 | 1,000 | 1,000 | 0,000 | 0,000 |
| Spironolactone | 0,000 | 0,004 | 0,000 | 0,000 | 0,001 | 0,000 | 1,000 | 0,001 | 1,000 | 0,141 | 0,000 | 0,000 |
| Cetirizine | 0,000 | 0,000 | 0,000 | 0,961 | 0,000 | 0,000 | 0,000 | 0,400 | 0,937 | 0,000 | 0,000 | 0,000 |
| Atenolol | 0,000 | 0,000 | 0,000 | 0,000 | 0,000 | 0,000 | 0,000 | 0,391 | 0,633 | 0,001 | 0,999 | 0,999 |
| Oxycodone | 0,000 | 0,000 | 0,000 | 0,000 | 0,000 | 0,000 | 0,000 | 0,391 | 0,633 | 0,001 | 0,999 | 0,999 |
| Buspirone | 0,000 | 0,016 | 0,000 | 0,000 | 0,000 | 0,000 | 0,000 | 0,980 | 0,635 | 0,000 | 0,933 | 0,933 |
| Salmeterol | 0,000 | 0,000 | 0,000 | 0,858 | 0,002 | 0,379 | 0,504 | 0,029 | 0,092 | 0,000 | 0,018 | 0,018 |
| Topiramate | 0,000 | 0,000 | 0,000 | 0,000 | 0,000 | 0,000 | 0,000 | 0,000 | 0,936 | 0,001 | 0,000 | 0,000 |
| Warfarin | 0,000 | 0,003 | 0,999 | 0,000 | 0,018 | 0,072 | 0,014 | 0,999 | 1,000 | 1,000 | 0,987 | 0,987 |
| Estradiol | 0,104 | 0,013 | 0,030 | 0,010 | 0,001 | 0,000 | 0,893 | 0,787 | 0,945 | 0,996 | 1,000 | 1,000 |
| Cholecalciferol | 0,000 | 0,001 | 0,053 | 0,000 | 0,000 | 0,999 | 0,999 | 0,001 | 0,967 | 0,139 | 0,002 | 0,002 |
| Budesonide | 0,000 | 0,000 | 0,000 | 0,000 | 1,000 | 0,000 | 0,000 | 0,903 | 0,973 | 1,000 | 0,064 | 0,064 |
| Formoterol | 0,541 | 0,661 | 0,013 | 1,000 | 0,517 | 0,002 | 0,947 | 0,908 | 1,000 | 0,990 | 1,000 | 1,000 |
| Lamotrigine | 0,153 | 0,000 | 0,000 | 0,000 | 0,000 | 0,000 | 0,001 | 0,936 | 0,000 | 0,012 | 0,000 | 0,000 |
| Norgestimate | 0,012 | 1,000 | 1,000 | 0,028 | 0,001 | 0,000 | 1,000 | 0,018 | 0,575 | 1,000 | 0,818 | 0,818 |
| Quetlapine | 0,002 | 0,001 | 0,000 | 1,000 | 0,002 | 0,000 | 0,000 | 1,000 | 0,999 | 1,000 | 1,000 | 1,000 |
| Lorazepam | 0,995 | 0,000 | 0,000 | 0,000 | 0,001 | 0,000 | 0,000 | 1,000 | 0,000 | 0,000 | 0,000 | 0,000 |
| Famotidine | 0,001 | 0,000 | 0,000 | 0,000 | 0,000 | 0,000 | 0,000 | 0,000 | 0,000 | 0,000 | 0,000 | 0,000 |
| Folic Acid | 0,000 | 0,000 | 0,000 | 0,000 | 0,000 | 0,000 | 0,000 | 0,000 | 0,000 | 0,957 | 0,000 | 0,000 |
| Azithromycin | 0,000 | 0,000 | 0,000 | 0,000 | 0,000 | 0,000 | 0,000 | 0,332 | 1,000 | 0,001 | 0,000 | 0,000 |
| Hydroxyzine | 0,000 | 0,000 | 0,000 | 1,000 | 0,001 | 0,000 | 0,000 | 0,855 | 0,957 | 0,000 | 0,000 | 0,000 |
| Insulin Lispro | 0,000 | 0,000 | 0,000 | 0,000 | 0,000 | 0,000 | 0,000 | 0,000 | 0,000 | 0,000 | 0,000 | 0,000 |
| Diclofenac | 0,000 | 0,000 | 1,000 | 0,019 | 0,000 | 0,016 | 0,000 | 1,000 | 1,000 | 1,000 | 0,073 | 0,073 |
| Loratadine | 0,000 | 0,978 | 0,998 | 0,008 | 0,007 | 1,000 | 0,287 | 0,170 | 1,000 | 0,996 | 0,991 | 0,991 |
| Sitagliptin | 0,000 | 0,000 | 0,000 | 0,000 | 0,000 | 0,000 | 0,142 | 0,572 | 0,954 | 0,003 | 0,000 | 0,000 |
| Clonidine | 0,000 | 0,000 | 0,000 | 1,000 | 0,000 | 0,000 | 0,000 | 1,000 | 0,796 | 1,000 | 0,997 | 0,997 |
| Diltiazem | 0,000 | 0,000 | 0,002 | 0,001 | 0,986 | 0,000 | 0,035 | 0,999 | 1,000 | 0,999 | 0,999 | 0,999 |
| Latanoprost | 0,000 | 0,000 | 0,000 | 0,000 | 0,012 | 0,000 | 0,960 | 0,000 | 0,014 | 0,148 | 0,003 | 0,003 |
| Pregabalin | 0,000 | 0,000 | 0,000 | 0,000 | 0,000 | 0,000 | 0,000 | 0,000 | 0,991 | 0,899 | 0,665 | 0,665 |
| Doxycycline | 0,000 | 0,000 | 0,000 | 0,000 | 0,000 | 0,000 | 0,000 | 0,001 | 0,000 | 0,877 | 0,000 | 0,000 |
| Insulin Aspart | 0,000 | 0,000 | 0,000 | 0,000 | 0,000 | 0,000 | 0,000 | 0,000 | 1,000 | 0,000 | 0,000 | 0,000 |
| Amitriptyline | 0,003 | 0,000 | 0,000 | 1,000 | 0,000 | 0,000 | 0,000 | 1,000 | 1,000 | 0,885 | 1,000 | 1,000 |
| Paroxetine | 1,000 | 0,008 | 0,000 | 0,044 | 1,000 | 1,000 | 0,000 | 0,989 | 1,000 | 0,001 | 1,000 | 1,000 |
| Ondansetron | 1,000 | 0,001 | 0,000 | 0,982 | 0,380 | 0,000 | 0,002 | 1,000 | 0,985 | 0,996 | 0,995 | 0,995 |
| Tizanidine | 0,007 | 0,000 | 0,000 | 0,668 | 0,000 | 0,000 | 0,000 | 1,000 | 0,969 | 0,227 | 0,975 | 0,975 |
| Lisdexamfetamine | 0,020 | 0,001 | 0,000 | 0,002 | 0,027 | 0,235 | 0,000 | 0,000 | 0,449 | 0,003 | 0,591 | 0,591 |
| Rivaroxaban | 0,764 | 1,000 | 1,000 | 0,723 | 0,994 | 0,985 | 1,000 | 0,999 | 0,978 | 1,000 | 0,000 | 0,000 |
| Glimepiride | 0,000 | 0,000 | 0,835 | 0,000 | 0,000 | 0,000 | 0,000 | 0,000 | 1,000 | 1,000 | 0,000 | 0,000 |
| Propranolol | 1,000 | 0,000 | 0,000 | 0,969 | 0,000 | 0,000 | 0,000 | 0,999 | 1,000 | 0,067 | 1,000 | 1,000 |
| Aripiprazole | 0,000 | 0,010 | 0,000 | 0,059 | 0,001 | 0,001 | 0,012 | 0,993 | 0,111 | 0,000 | 0,999 | 0,999 |
| Finasteride | 0,000 | 0,752 | 0,000 | 0,000 | 0,769 | 0,000 | 0,000 | 0,001 | 1,000 | 0,741 | 0,566 | 0,566 |
| Naproxen | 0,022 | 0,001 | 0,005 | 0,000 | 0,000 | 0,000 | 0,001 | 0,991 | 0,999 | 1,000 | 0,052 | 0,052 |
| Levetiracetam | 0,000 | 0,008 | 0,000 | 0,000 | 0,000 | 0,000 | 0,281 | 0,010 | 0,995 | 0,001 | 0,001 | 0,001 |
| Alendronate | 0,000 | 0,000 | 0,011 | 0,000 | 0,000 | 0,000 | 0,000 | 0,000 | 0,000 | 0,879 | 0,000 | 0,000 |
| Fenofibrate | 0,998 | 0,995 | 0,987 | 0,000 | 0,000 | 1,000 | 1,000 | 0,028 | 0,000 | 0,000 | 0,000 | 0,000 |
| Oxybutynin | 1,000 | 0,997 | 0,982 | 1,000 | 1,000 | 0,000 | 1,000 | 0,507 | 0,002 | 0,000 | 0,000 | 0,000 |
| Celecoxib | 0,837 | 0,895 | 0,974 | 0,000 | 0,008 | 0,000 | 1,000 | 0,887 | 0,000 | 1,000 | 1,000 | 1,000 |
| Lovastatin | 0,000 | 0,000 | 0,003 | 0,000 | 1,000 | 0,000 | 0,993 | 0,004 | 0,842 | 0,002 | 0,000 | 0,000 |
| Ezetimibe | 0,000 | 0,048 | 0,725 | 0,005 | 0,432 | 0,015 | 0,999 | 0,012 | 0,127 | 0,009 | 0,339 | 0,339 |

>0.5 (orange): inhibitor/substrate; <0.5 (green): non-inhibitor/non-substrate

**Table S 12. ESP Testing Results** (Substrates)

| **2** | **1A2** | **2C19** | **2C9** | **2A6** | **2B6** | **2D6** | **2C8** | **2E1** | **3A4** |
| --- | --- | --- | --- | --- | --- | --- | --- | --- | --- |
| Atorvastatin | 0.0060 | 0.0043 | 0.0010 | 0.0052 | 0.0138 | 0.0250 | 0.0009 | 0.0103 | 0.0017 |
| Levothyroxine | 0.0165 | 0.0212 | 0.0127 | 0.0103 | 0.0180 | 0.0148 | 0.0062 | 0.0081 | 0.0171 |
| Metformin | 0.0904 | 0.0109 | 0.0067 | 0.0146 | 0.0366 | 0.0300 | 0.0101 | 0.0167 | 0.0449 |
| Lisinopril | 0.0100 | 0.0053 | 0.0046 | 0.0062 | 0.0171 | 0.0082 | 0.0103 | 0.0101 | 0.0414 |
| Amlodipine | 0.0051 | 0.0032 | 0.0032 | 0.0042 | 0.0066 | 0.0018 | 0.0021 | 0.0015 | 0.0029 |
| Metoprolol | 0.0223 | 0.0161 | 0.0045 | 0.0131 | 0.0579 | 0.0137 | 0.0154 | 0.0151 | 0.0146 |
| Albuterol | 0.0184 | 0.0085 | 0.0078 | 0.0120 | 0.0257 | 0.0349 | 0.0130 | 0.0106 | 0.0133 |
| Omeprazole | 0.0241 | 0.0221 | 0.0038 | 0.0233 | 0.0170 | 0.0123 | 0.0060 | 0.0137 | 0.0385 |
| Losartan | 0.1127 | 0.0237 | 0.0124 | 0.0400 | 0.0568 | 0.0376 | 0.0194 | 0.0154 | 0.0229 |
| Gabapentin | 0.2164 | 0.1487 | 0.0647 | 0.0894 | 0.1234 | 0.1957 | 0.0903 | 0.0875 | 0.0491 |
| Hydrochlorothiazide | 0.1377 | 0.1411 | 0.1030 | 0.3858 | 0.1951 | 0.1717 | 0.2444 | 0.3937 | 0.0705 |
| Sertraline | 0.2796 | 0.1396 | 0.2354 | 0.1964 | 0.1582 | 0.1346 | 0.1782 | 0.1165 | 0.0774 |
| Simvastatin | 0.0499 | 0.0564 | 0.0439 | 0.0476 | 0.0400 | 0.0244 | 0.0351 | 0.0491 | 0.0171 |
| Montelukast | 0.0015 | 0.0012 | 0.0011 | 0.0023 | 0.0002 | 0.0002 | 0.0012 | 0.0017 | 0.0013 |
| Escitalopram | 0.0807 | 0.0377 | 0.0182 | 0.0680 | 0.0169 | 0.0714 | 0.0198 | 0.0332 | 0.0025 |
| Acetaminophen | 0.2080 | 0.1373 | 0.1031 | 0.0847 | 0.0516 | 0.0967 | 0.1181 | 0.0528 | 0.0472 |
| Hydrocodone | 0.1116 | 0.0723 | 0.0227 | 0.0628 | 0.0601 | 0.0602 | 0.0638 | 0.0275 | 0.0075 |
| Rosuvastatin | 0.0157 | 0.0082 | 0.0049 | 0.0215 | 0.0535 | 0.0084 | 0.0031 | 0.0228 | 0.0114 |
| Bupropion | 0.1045 | 0.1827 | 0.2705 | 0.1847 | 0.1259 | 0.1264 | 0.3854 | 0.1369 | 0.2651 |
| Furosemide | 0.0130 | 0.0075 | 0.0045 | 0.0083 | 0.0105 | 0.0047 | 0.0023 | 0.0035 | 0.0025 |
| Pantoprazole | 0.0238 | 0.0069 | 0.0067 | 0.0102 | 0.0068 | 0.0036 | 0.0012 | 0.0090 | 0.0291 |
| Trazodone | 0.0556 | 0.0458 | 0.0296 | 0.0480 | 0.0685 | 0.0409 | 0.0520 | 0.0445 | 0.1896 |
| Dextroamphetamine | 0.4966 | 0.3889 | 0.2300 | 0.2333 | 0.3691 | 0.2988 | 0.3782 | 0.1765 | 0.2455 |
| Dextroamphetamine Saccharate | 0.0600 | 0.0490 | 0.0250 | 0.0228 | 0.0388 | 0.0326 | 0.0329 | 0.0100 | 0.1251 |
| Amphetamine Aspartate | 0.1014 | 0.0718 | 0.0419 | 0.0614 | 0.0572 | 0.0853 | 0.0679 | 0.0415 | 0.0087 |
| Fluticasone | 0.0080 | 0.0078 | 0.0320 | 0.0034 | 0.0067 | 0.0010 | 0.0020 | 0.0016 | 0.0027 |
| Tamsulosin | 0.1313 | 0.0035 | 0.0034 | 0.0048 | 0.0324 | 0.0073 | 0.0049 | 0.0103 | 0.0011 |
| Fluoxetine | 0.4995 | 0.2554 | 0.1039 | 0.3074 | 0.5345 | 0.3332 | 0.6603 | 0.2877 | 0.0507 |
| Carvedilol | 0.0462 | 0.0228 | 0.0152 | 0.0239 | 0.0975 | 0.0438 | 0.0131 | 0.0257 | 0.0155 |
| Duloxetine | 0.0419 | 0.0247 | 0.0125 | 0.0591 | 0.1009 | 0.0302 | 0.0259 | 0.0266 | 0.0097 |
| Meloxicam | 0.1229 | 0.0342 | 0.0268 | 0.0284 | 0.0167 | 0.0449 | 0.0344 | 0.0276 | 0.0331 |
| Clopidogrel | 0.1223 | 0.0949 | 0.0381 | 0.0708 | 0.0320 | 0.0116 | 0.1814 | 0.0209 | 0.0822 |
| Prednisone | 0.0926 | 0.0937 | 0.0920 | 0.1170 | 0.0265 | 0.0287 | 0.0538 | 0.0593 | 0.0039 |
| Citalopram | 0.0807 | 0.0377 | 0.0182 | 0.0680 | 0.0169 | 0.0714 | 0.0198 | 0.0332 | 0.0025 |
| Insulin Glargine | 0.0000 | 0.0000 | 0.0000 | 0.0000 | 0.0000 | 0.0000 | 0.0000 | 0.0000 | 0.0000 |
| Potassium Chloride | 0.9681 | 0.6427 | 0.5058 | 0.8177 | 0.7932 | 0.7774 | 0.8941 | 0.7694 | 0.4885 |
| Pravastatin | 0.1438 | 0.0299 | 0.0055 | 0.0165 | 0.0837 | 0.0818 | 0.0165 | 0.0701 | 0.0122 |
| Tramadol | 0.1520 | 0.0758 | 0.0195 | 0.0222 | 0.1091 | 0.0743 | 0.0175 | 0.0395 | 0.0225 |
| Aspirin | 0.4510 | 0.6555 | 0.6341 | 0.5363 | 0.2787 | 0.4503 | 0.6277 | 0.2883 | 0.3153 |
| Alprazolam | 0.6879 | 0.7297 | 0.7157 | 0.5581 | 0.3900 | 0.2658 | 0.6176 | 0.3670 | 0.2619 |
| Ibuprofen | 0.0605 | 0.0226 | 0.0238 | 0.0177 | 0.0359 | 0.0115 | 0.0143 | 0.0137 | 0.0447 |
| Cyclobenzaprine | 0.2515 | 0.0842 | 0.0226 | 0.1071 | 0.1081 | 0.1911 | 0.0624 | 0.1941 | 0.0054 |
| Amoxicillin | 0.0037 | 0.0075 | 0.0040 | 0.0051 | 0.0129 | 0.0018 | 0.0069 | 0.0050 | 0.0013 |
| Methylphenidate | 0.3376 | 0.1958 | 0.1055 | 0.3522 | 0.1892 | 0.4378 | 0.4464 | 0.2417 | 0.1024 |
| Allopurinol | 0.9066 | 0.6109 | 0.6070 | 0.8166 | 0.7133 | 0.7563 | 0.8123 | 0.7513 | 0.2536 |
| Venlafaxine | 0.0414 | 0.0105 | 0.0040 | 0.0121 | 0.0531 | 0.0112 | 0.0078 | 0.0101 | 0.0115 |
| Clonazepam | 0.2257 | 0.1113 | 0.0377 | 0.2348 | 0.1291 | 0.3056 | 0.1840 | 0.5766 | 0.0006 |
| Ethinyl Estradiol | 0.4819 | 0.1444 | 0.0978 | 0.0903 | 0.1282 | 0.1760 | 0.1941 | 0.1289 | 0.0169 |
| Ergocalciferol | 0.0092 | 0.0452 | 0.0477 | 0.0870 | 0.0361 | 0.0811 | 0.3267 | 0.0392 | 0.0169 |
| Zolpidem | 0.2402 | 0.1309 | 0.0817 | 0.0672 | 0.0781 | 0.1035 | 0.0768 | 0.0714 | 0.2078 |
| Apixaban | 0.0106 | 0.0402 | 0.0458 | 0.0207 | 0.0174 | 0.0163 | 0.0473 | 0.0063 | 0.0209 |
| Glipizide | 0.0103 | 0.0015 | 0.0008 | 0.0037 | 0.0065 | 0.0050 | 0.0010 | 0.0130 | 0.0174 |
| Spironolactone | 0.3486 | 0.2091 | 0.4529 | 0.2466 | 0.0847 | 0.1959 | 0.5153 | 0.1784 | 0.3653 |
| Cetirizine | 0.0025 | 0.0008 | 0.0002 | 0.0003 | 0.0017 | 0.0026 | 0.0015 | 0.0008 | 0.0012 |
| Atenolol | 0.0112 | 0.0195 | 0.0079 | 0.0115 | 0.0161 | 0.0110 | 0.0105 | 0.0123 | 0.0338 |
| Oxycodone | 0.2162 | 0.0722 | 0.0484 | 0.0853 | 0.1285 | 0.0684 | 0.0565 | 0.0554 | 0.0170 |
| Buspirone | 0.0089 | 0.0102 | 0.0028 | 0.0063 | 0.0014 | 0.0028 | 0.0066 | 0.0040 | 0.0317 |
| Salmeterol | 0.0021 | 0.0004 | 0.0003 | 0.0003 | 0.0036 | 0.0012 | 0.0014 | 0.0021 | 0.0001 |
| Topiramate | 0.0277 | 0.0092 | 0.0031 | 0.0057 | 0.0119 | 0.0048 | 0.0034 | 0.0075 | 0.0192 |
| Warfarin | 0.0483 | 0.1020 | 0.0619 | 0.0195 | 0.0378 | 0.0914 | 0.0757 | 0.0219 | 0.0268 |
| Estradiol | 0.2543 | 0.0779 | 0.1442 | 0.1242 | 0.4825 | 0.0898 | 0.0964 | 0.1549 | 0.0599 |
| Cholecalciferol | 0.0020 | 0.0415 | 0.0133 | 0.0094 | 0.0335 | 0.0231 | 0.0436 | 0.0097 | 0.0097 |
| Budesonide | 0.0002 | 0.0013 | 0.0009 | 0.0013 | 0.0023 | 0.0014 | 0.0006 | 0.0005 | 0.0015 |
| Formoterol | 0.0072 | 0.0004 | 0.0001 | 0.0003 | 0.0006 | 0.0009 | 0.0001 | 0.0007 | 0.0008 |
| Lamotrigine | 0.1527 | 0.0544 | 0.0465 | 0.1889 | 0.0823 | 0.0584 | 0.1823 | 0.1101 | 0.1542 |
| Norgestimate | 0.0505 | 0.3472 | 0.2833 | 0.0434 | 0.0684 | 0.0716 | 0.0971 | 0.0997 | 0.0192 |
| Quetlapine | 0.0044 | 0.0006 | 0.0003 | 0.0020 | 0.0342 | 0.0063 | 0.0011 | 0.0036 | 0.0021 |
| Lorazepam | 0.2023 | 0.1437 | 0.0736 | 0.2466 | 0.2756 | 0.1361 | 0.2352 | 0.2402 | 0.1263 |
| Famotidine | 0.2770 | 0.0095 | 0.0080 | 0.0183 | 0.0515 | 0.0086 | 0.0141 | 0.0083 | 0.0360 |
| Folic Acid | 0.0039 | 0.0012 | 0.0005 | 0.0015 | 0.0015 | 0.0044 | 0.0011 | 0.0004 | 0.0005 |
| Azithromycin | 0.0029 | 0.0024 | 0.0038 | 0.0014 | 0.0032 | 0.0030 | 0.0021 | 0.0009 | 0.0002 |
| Hydroxyzine | 0.0029 | 0.0004 | 0.0001 | 0.0006 | 0.0055 | 0.0080 | 0.0010 | 0.0016 | 0.0003 |
| Insulin Lispro | 0.0005 | 0.0004 | 0.0003 | 0.0001 | 0.0007 | 0.0005 | 0.0001 | 0.0001 | 0.0000 |
| Diclofenac | 0.0354 | 0.0092 | 0.0070 | 0.0073 | 0.0142 | 0.0294 | 0.0058 | 0.0125 | 0.0092 |
| Loratadine | 0.0053 | 0.0428 | 0.0079 | 0.0106 | 0.0040 | 0.0036 | 0.0148 | 0.0116 | 0.0388 |
| Sitagliptin | 0.0540 | 0.0497 | 0.0163 | 0.0635 | 0.0577 | 0.0432 | 0.0090 | 0.0311 | 0.0186 |
| Clonidine | 0.3280 | 0.0799 | 0.0373 | 0.1608 | 0.1279 | 0.0648 | 0.1877 | 0.2611 | 0.0483 |
| Diltiazem | 0.0008 | 0.0019 | 0.0007 | 0.0016 | 0.0037 | 0.0012 | 0.0003 | 0.0017 | 0.0048 |
| Latanoprost | 0.0020 | 0.0010 | 0.0008 | 0.0004 | 0.0049 | 0.0006 | 0.0003 | 0.0009 | 0.0003 |
| Pregabalin | 0.0687 | 0.0879 | 0.0495 | 0.0623 | 0.0719 | 0.0737 | 0.0490 | 0.0276 | 0.0393 |
| Doxycycline | 0.0016 | 0.0011 | 0.0004 | 0.0005 | 0.0007 | 0.0004 | 0.0013 | 0.0008 | 0.0090 |
| Insulin Aspart | 0.0000 | 0.0000 | 0.0000 | 0.0000 | 0.0000 | 0.0000 | 0.0000 | 0.0000 | 0.0000 |
| Amitriptyline | 0.4301 | 0.0918 | 0.0428 | 0.1033 | 0.0957 | 0.1977 | 0.0781 | 0.2097 | 0.0074 |
| Paroxetine | 0.0034 | 0.0037 | 0.0007 | 0.0016 | 0.0039 | 0.0020 | 0.0049 | 0.0058 | 0.0014 |
| Ondansetron | 0.1374 | 0.2729 | 0.1737 | 0.1470 | 0.1084 | 0.0842 | 0.2531 | 0.0886 | 0.0749 |
| Tizanidine | 0.1124 | 0.0094 | 0.0027 | 0.0140 | 0.0127 | 0.0086 | 0.0229 | 0.0425 | 0.0086 |
| Lisdexamfetamine | 0.0399 | 0.0496 | 0.0200 | 0.0194 | 0.0434 | 0.0090 | 0.0358 | 0.0172 | 0.0670 |
| Rivaroxaban | 0.0120 | 0.0012 | 0.0011 | 0.0018 | 0.0081 | 0.0037 | 0.0014 | 0.0016 | 0.0043 |
| Glimepiride | 0.0629 | 0.0113 | 0.0069 | 0.0188 | 0.0147 | 0.0328 | 0.0102 | 0.0799 | 0.0166 |
| Propranolol | 0.1487 | 0.0482 | 0.0176 | 0.1049 | 0.2370 | 0.0583 | 0.0197 | 0.0960 | 0.0983 |
| Aripiprazole | 0.0112 | 0.0125 | 0.0025 | 0.0051 | 0.0023 | 0.0042 | 0.0133 | 0.0044 | 0.0051 |
| Finasteride | 0.1090 | 0.0519 | 0.0400 | 0.1007 | 0.0211 | 0.0296 | 0.1408 | 0.0405 | 0.0051 |
| Naproxen | 0.1031 | 0.1941 | 0.1798 | 0.2809 | 0.1094 | 0.0949 | 0.3093 | 0.1794 | 0.0795 |
| Levetiracetam | 0.0422 | 0.0516 | 0.0311 | 0.0418 | 0.0428 | 0.0430 | 0.0295 | 0.0247 | 0.1164 |
| Alendronate | 0.2574 | 0.0774 | 0.0382 | 0.0845 | 0.1148 | 0.0433 | 0.0677 | 0.1190 | 0.1111 |
| Fenofibrate | 0.0529 | 0.0450 | 0.0472 | 0.0376 | 0.0231 | 0.0137 | 0.1382 | 0.0490 | 0.0635 |
| Oxybutynin | 0.0130 | 0.0265 | 0.0155 | 0.0121 | 0.0227 | 0.0063 | 0.0265 | 0.0184 | 0.0074 |
| Celecoxib | 0.1633 | 0.1109 | 0.0540 | 0.2552 | 0.2693 | 0.0832 | 0.1055 | 0.1585 | 0.2655 |
| Lovastatin | 0.0982 | 0.1043 | 0.0919 | 0.1030 | 0.1430 | 0.0397 | 0.0744 | 0.1501 | 0.0109 |
| Ezetimibe | 0.0019 | 0.0022 | 0.0007 | 0.0007 | 0.0035 | 0.0014 | 0.0023 | 0.0013 | 0.0016 |

≥0.5 (orange): substrate; <0.5 (green): non-substrate

**Table S 12. Summary of mo3del performance metrics for CYP450s inhibitors prediction**

| **Isoforms** | **1A2** | **2C9** | **2C19** | **2D6** | **3A4** | **2C8** | **All** |
| --- | --- | --- | --- | --- | --- | --- | --- |
| **pkCSM** |  | | | | | | |
| TP | 5 | 2 | 5 | 9 | 4 |  | 25 |
| TN | 69 | 77 | 74 | 70 | 70 |  | 360 |
| FP | 24 | 14 | 17 | 13 | 11 |  | 79 |
| FN | 2 | 7 | 4 | 8 | 15 |  | 36 |
| Sum | 100 | 100 | 100 | 100 | 100 |  | 500 |
| SN | 0.71 | 0.22 | 0.55 | 0.53 | 0.21 |  | 0.45 |
| SP | 0.74 | 0.85 | 0.81 | 0.84 | 0.86 |  | 0.82 |
| ACC | 0.74 | 0.79 | 0.79 | 0.79 | 0.74 |  | 0.77 |
| MCC | 0.26 | 0.05 | 0.25 | 0.34 | 0.08 |  | 0.20 |
| F1 | 0.28 | 0.16 | 0.32 | 0.46 | 0.24 |  | 0.30 |
| **vNN-ADMET** |  |  |  |  |  |  |  |
| TP | 1 | 1 | 3 | 3 | 5 |  | 13 |
| TN | 48 | 46 | 48 | 37 | 54 |  | 233 |
| FP | 3 | 8 | 9 | 8 | 3 |  | 31 |
| FN | 6 | 5 | 1 | 6 | 10 |  | 28 |
| No Prediction | 42 | 40 | 39 | 46 | 28 |  | 195 |
| Sum | 100 | 100 | 100 | 100 | 100 |  | 500 |
| SN | 0.14 | 0.17 | 0.75 | 0.33 | 0.33 |  | 0.34 |
| SP | 0.94 | 0.85 | 0.84 | 0.82 | 0.95 |  | 0.88 |
| ACC | 0.84 | 0.78 | 0.84 | 0.74 | 0.82 |  | 0.80 |
| MCC | 0.11 | 0.02 | 0.37 | 0.14 | 0.36 |  | 0.20 |
| F1 | 0.18 | 0.13 | 0.38 | 0.30 | 0.43 |  | 0.31 |
| **SwissADME** |  | | | | | | |
| TP | 2 | 6 | 7 | 14 | 11 |  | 40 |
| TN | 70 | 65 | 67 | 50 | 56 |  | 308 |
| FP | 19 | 22 | 20 | 29 | 20 |  | 110 |
| FN | 5 | 3 | 2 | 3 | 9 |  | 22 |
| No Prediction | 4 | 4 | 4 | 4 | 4 |  | 20 |
| Sum | 100 | 100 | 100 | 100 | 100 |  | 500 |
| SN | 0.29 | 0.67 | 0.78 | 0.82 | 0.55 |  | 0.62 |
| SP | 0.79 | 0.75 | 0.77 | 0.63 | 0.74 |  | 0.73 |
| ACC | 0.75 | 0.74 | 0.77 | 067 | 0,70 |  | 0.72 |
| MCC | 0.04 | 0.27 | 0.36 | 0.35 | 0.25 |  | 0.20 |
| F1 | 0.14 | 0.32 | 0.39 | 0.47 | 0.43 |  | 0.38 |
| **admetSAR 2.0** |  | | | | | | |
| TP | 3 | 2 | 4 | 5 | 8 | 2 | 24 |
| TN | 68 | 72 | 71 | 68 | 65 | 75 | 419 |
| FP | 25 | 19 | 20 | 15 | 15 | 21 | 115 |
| FN | 4 | 7 | 5 | 12 | 12 | 2 | 42 |
| Sum | 100 | 100 | 100 | 100 | 100 | 100 | 600 |
| SN | 0.43 | 0.22 | 0.44 | 0.29 | 0.40 | 0.50 | 0.38 |
| SP | 0.73 | 0.79 | 0.78 | 0.82 | 0.81 | 0.78 | 0.79 |
| ACC | 0.71 | 0.74 | 0.75 | 0.73 | 0.73 | 0.77 | 0.74 |
| MCC | 0.09 | 0.01 | 0.15 | 0.11 | 0.20 | 0.13 | 0.12 |
| F1 | 0.17 | 0.13 | 0.24 | 0.27 | 0.37 | 0.15 | 0.23 |
| **SuperCYPsPred** | MACCS fingerprints | | | | | | |
| TP | 1 | 3 | 4 | 11 | 8 |  | 27 |
| TN | 88 | 63 | 83 | 58 | 75 |  | 367 |
| FP | 4 | 27 | 7 | 24 | 4 |  | 66 |
| FN | 6 | 6 | 5 | 6 | 12 |  | 35 |
| No Prediction | 1 | 1 | 1 | 1 | 1 |  | 5 |
| Sum | 100 | 100 | 100 | 100 | 100 |  | 500 |
| SN | 0.14 | 0.33 | 0.44 | 0.65 | 0.40 |  | 0.39 |
| SP | 0.96 | 0.70 | 0.92 | 0.71 | 0.95 |  | 0.85 |
| ACC | 0.90 | 0.67 | 0.88 | 0.70 | 0.84 |  | 0.80 |
| MCC | 0.12 | 0.02 | 0.34 | 0.28 | 0.43 |  | 0.24 |
| F1 | 0.17 | 0.15 | 0.40 | 0.42 | 0.50 |  | 0.35 |
| **SuperCPYsPred** | Morgan fingerprints | | | | | | |
| TP | 1 | 1 | 3 | 12 | 6 |  | 23 |
| TN | 84 | 83 | 84 | 56 | 70 |  | 377 |
| FP | 8 | 7 | 6 | 26 | 9 |  | 56 |
| FN | 6 | 8 | 6 | 5 | 14 |  | 39 |
| No Prediction | 1 | 1 | 1 | 1 | 1 |  | 5 |
| Sum | 100 | 100 | 100 | 100 | 100 |  | 500 |
| SN | 0.14 | 0.11 | 0.33 | 0.71 | 0.30 |  | 0.32 |
| SP | 0.91 | 0.92 | 0.93 | 0.68 | 0.89 |  | 0.87 |
| ACC | 0.86 | 0.85 | 0.88 | 0.69 | 0.77 |  | 0.81 |
| MCC | 0.05 | 0.04 | 0.27 | 0.30 | 0.21 |  | 0.17 |
| F1 | 0.13 | 0.12 | 0.33 | 0.44 | 0.34 |  | 0.33 |
| **CYPlebrity** |  | | | | | | |
| TP | 3 | 4 | 6 | 11 | 9 |  | 33 |
| TN | 73 | 75 | 71 | 66 | 65 |  | 350 |
| FP | 16 | 12 | 16 | 13 | 11 |  | 68 |
| FN | 4 | 5 | 3 | 6 | 11 |  | 29 |
| No Prediction | 4 | 4 | 4 | 4 | 4 |  | 20 |
| Sum | 100 | 100 | 100 | 100 | 100 |  | 500 |
| SN | 0.43 | 0.44 | 0.67 | 0.65 | 0.45 |  | 0.53 |
| SP | 0.82 | 0.86 | 0.82 | 0.84 | 0.86 |  | 0.84 |
| ACC | 0.79 | 0.82 | 0.80 | 0.80 | 0.77 |  | 0.80 |
| MCC | 0.16 | 0.24 | 0.33 | 0.43 | 0.31 |  | 0.30 |
| F1 | 0.23 | 0.32 | 0.39 | 0.54 | 0.45 |  | 0.40 |
| **admetLab 2.0** |  | | | | | | |
| TP | 4 | 4 | 6 | 11 | 10 |  | 35 |
| TN | 75 | 74 | 70 | 58 | 57 |  | 334 |
| FP | 17 | 16 | 20 | 24 | 22 |  | 99 |
| FN | 3 | 5 | 3 | 6 | 10 |  | 27 |
| No Prediction | 1 | 1 | 1 | 1 | 1 |  | 5 |
| Sum | 100 | 100 | 100 | 100 | 100 |  | 500 |
| SN | 0.57 | 0.44 | 0.67 | 0.65 | 0.50 |  | 0.57 |
| SP | 0.82 | 0.82 | 0.78 | 0.71 | 0.72 |  | 0.77 |
| ACC | 0.80 | 0.79 | 0.77 | 0,.70 | 0.68 |  | 0.75 |
| MCC | 0.24 | 0.19 | 0.29 | 0.28 | 0.19 |  | 0.24 |
| F1 | 0.29 | 0.28 | 0.34 | 0.42 | 0.38 |  | 0.36 |
| **admetLab 3.0** |  | | | | | | |
| TP | 3 | 2 | 7 | 5 | 5 | 2 | 29 |
| TN | 76 | 81 | 79 | 66 | 71 | 69 | 528 |
| FP | 17 | 13 | 12 | 17 | 15 | 27 | 109 |
| FN | 4 | 4 | 2 | 12 | 9 | 2 | 34 |
| Sum | 100 | 100 | 100 | 100 | 100 | 100 | 700 |
| SN | 0.43 | 0.33 | 0.78 | 0.29 | 0.36 | 0.5 | 0.46 |
| SP | 0.82 | 0.86 | 0.87 | 0.79 | 0.82 | 0.72 | 0.83 |
| ACC | 0.79 | 0.83 | 0.86 | 0.71 | 0.76 | 0.71 | 0.79 |
| MCC | 0.16 | 0.13 | 0.47 | 0.08 | 0.16 | 0.09 | 0.21 |
| F1 | 0.22 | 0.19 | 0.5 | 0.25 | 0.29 | 0.12 | 0.29 |

**Table S 13. Summary of model performance metrics for CYP450s substrates prediction**

| **Isoforms** | **1A2** | **2C9** | **2C19** | **2D6** | **3A4** | **2A6** | **2B6** | **2C8** | **2E1** | **All** |
| --- | --- | --- | --- | --- | --- | --- | --- | --- | --- | --- |
| **pkCSM** |  | | | | | | | | | |
| TP |  |  |  | 4 | 35 |  |  |  |  | 39 |
| TN |  |  |  | 67 | 31 |  |  |  |  | 98 |
| FP |  |  |  | 5 | 9 |  |  |  |  | 14 |
| FN |  |  |  | 24 | 26 |  |  |  |  | 50 |
| Sum |  |  |  | 100 | 101 |  |  |  |  | 201 |
| SN |  |  |  | 0.14 | 0.57 |  |  |  |  | 0.36 |
| SP |  |  |  | 0.93 | 0.78 |  |  |  |  | 0.85 |
| ACC |  |  |  | 0.71 | 0.65 |  |  |  |  | 0.68 |
| MCC |  |  |  | 0.12 | 0.34 |  |  |  |  | 0.23 |
| F1 |  |  |  | 0.22 | 0.67 |  |  |  |  | 0.55 |
| **CypReact** |  |  |  |  |  |  |  |  |  |  |
| TP | 8 | 20 | 12 | 26 | 43 | 0 | 2 | 2 | 1 | 89 |
| TN | 58 | 38 | 37 | 42 | 10 | 90 | 73 | 64 | 91 | 90 |
| FP | 33 | 42 | 46 | 30 | 46 | 10 | 24 | 32 | 8 | 118 |
| FN | 1 | 0 | 5 | 2 | 1 | 0 | 1 | 2 | 0 | 3 |
| Sum | 100 | 100 | 100 | 100 | 100 | 100 | 100 | 100 | 100 | 300 |
| SN | 0.89 | 1.00 | 0.71 | 0.93 | 0.98 | - | 0.67 | 0.50 | 1.00 | 0.74 |
| SP | 0.64 | 0.48 | 0.45 | 0.58 | 0.18 | 0.90 | 0.75 | 0.67 | 0.92 | 0.62 |
| ACC | 0.66 | 0.58 | 0.49 | 0.68 | 0.53 | 0.90 | 0.75 | 0.66 | 0.92 | 0.69 |
| MCC | 0.31 | 0.39 | 0.12 | 0.46 | 0.25 | - | 0.16 | 0.07 | 0.32 | 0.23 |
| F1 | 0.32 | 0.49 | 0.32 | 0.62 | 0.65 | - | 0.14 | 0.11 | 0.2 | 0.32 |
| **admetSAR** |  |  |  |  |  |  |  |  |  |  |
| TP |  | 14 |  | 19 | 39 |  |  |  |  | 72 |
| TN |  | 71 |  | 61 | 26 |  |  |  |  | 158 |
| FP |  | 9 |  | 11 | 30 |  |  |  |  | 50 |
| FN |  | 6 |  | 9 | 5 |  |  |  |  | 20 |
| Sum |  | 100 |  | 100 | 100 |  |  |  |  | 300 |
| SN |  | 0.70 |  | 0.68 | 0.89 |  |  |  |  | 0.76 |
| SP |  | 0.89 |  | 0.85 | 0.46 |  |  |  |  | 0.73 |
| ACC |  | 0.85 |  | 0.80 | 0.65 |  |  |  |  | 0.77 |
| MCC |  | 0.56 |  | 0.52 | 0.38 |  |  |  |  | 0.48 |
| F1 |  | 0.65 |  | 0.66 | 0.38 |  |  |  |  | 0.56 |
| **CYPstrate** |  |  |  |  |  |  |  |  |  |  |
| TP | 4 | 14 | 11 | 19 | 34 | 0 | 2 | 1 | 1 | 86 |
| TN | 54 | 42 | 44 | 42 | 23 | 90 | 53 | 55 | 91 | 494 |
| FP | 11 | 7 | 9 | 3 | 15 | 1 | 11 | 18 | 2 | 77 |
| FN | 0 | 2 | 1 | 1 | 1 | 0 | 1 | 1 | 0 | 7 |
| No prediction | 31 | 35 | 35 | 35 | 27 | 9 | 33 | 25 | 6 | 236 |
| Sum | 100 | 100 | 100 | 100 | 100 | 100 | 100 | 100 | 100 | 900 |
| SN | 1.00 | 0.88 | 0.92 | 0.95 | 0.97 | - | 0.67 | 0.50 | 1.00 | 0.92 |
| SP | 0.83 | 0.86 | 0.83 | 0.93 | 0.61 | 0.99 | 0.83 | 0.75 | 0.98 | 0.87 |
| ACC | 0.84 | 0.86 | 0.85 | 0.94 | 0.78 | 0.99 | 0.82 | 0.75 | 0.98 | 0.87 |
| MCC | 0.47 | 0.67 | 0.63 | 0.86 | 0.61 | - | 0.26 | 0.09 | 0.57 | 0.64 |
| F1 | 0.42 | 0.76 | 0.69 | 0.90 | 0.81 | 0 | 0..25 | 0.10 | 0.5 | 0.67 |
| **admetLab 2.0** |  |  |  |  |  |  |  |  |  |  |
| TP | 15 | 18 | 17 | 27 | 40 |  |  |  |  | 85 |
| TN | 48 | 51 | 33 | 48 | 32 |  |  |  |  | 131 |
| FP | 36 | 28 | 49 | 23 | 23 |  |  |  |  | 74 |
| FN | 0 | 2 | 0 | 1 | 4 |  |  |  |  | 7 |
| No prediction | 1 | 1 | 1 | 1 | 1 |  |  |  |  | 3 |
| Sum | 100 | 100 | 100 | 100 | 100 |  |  |  |  | 300 |
| SN | 1.00 | 0.90 | 1.00 | 0.96 | 0.91 |  |  |  |  | 0.92 |
| SP | 0.57 | 0.65 | 0.40 | 0.68 | 0.58 |  |  |  |  | 0.63 |
| ACC | 0.64 | 0.70 | 0.51 | 0.76 | 0.73 |  |  |  |  | 0.73 |
| MCC | 0.41 | 0.44 | 0.32 | 0.58 | 0.51 |  |  |  |  | 0.51 |
| F1 | 0.45 | 0.55 | 0.41 | 0.69 | 0.76 |  |  |  |  | 0.68 |
| **admetLab 3.0** |  |  |  |  |  |  |  |  |  |  |
| TP | 9 | 12 | 13 | 26 | 42 |  | 2 |  |  | 104 |
| TN | 50 | 55 | 35 | 54 | 27 |  | 78 |  |  | 298 |
| FP | 40 | 31 | 51 | 2 | 29 |  | 0 |  |  | 154 |
| FN | 1 | 2 | 1 | 18 | 2 |  | 20 |  |  | 44 |
| Sum | 100 | 100 | 100 | 100 | 100 |  | 100 |  |  | 600 |
| SN | 0.90 | 0.86 | 0.93 | 0.59 | 0.95 |  | 0.09 |  |  | 0.70 |
| SP | 0.56 | 0.64 | 0.41 | 0.94 | 0.48 |  | 1 |  |  | 0.66 |
| ACC | 0.59 | 0.67 | 0.48 | 0.80 | 0.69 |  | 0.8 |  |  | 0.67 |
| MCC | 0.27 | 0.35 | 0.24 | 0.61 | 0.48 |  | 0.27 |  |  | 0.32 |
| F1 | 0.31 | 0.42 | 0.33 | 0.72 | 0.73 |  | 0.17 |  |  | 0.51 |
| **ESP** |  |  |  |  |  |  |  |  |  |  |
| TP | 0 | 1 | 0 | 0 | 0 | 0 | 0 | 1 | 0 | 2 |
| TN | 82 | 76 | 79 | 70 | 56 | 96 | 94 | 91 | 96 | 740 |
| FP | 3 | 3 | 4 | 2 | 0 | 4 | 3 | 5 | 3 | 27 |
| FN | 15 | 20 | 17 | 28 | 44 | 0 | 3 | 3 | 1 | 131 |
| Sum | 100 | 100 | 100 | 100 | 100 | 100 | 100 | 100 | 100 | 900 |
| SN | 0.00 | 0.05 | 0.00 | 0.00 | 0.00 | - | 0.00 | 0.25 | 0.00 | 0.04 |
| SP | 0,96 | 0,96 | 0,95 | 0.97 | 1.00 | 0.96 | 0.97 | 0.95 | 0.97 | 0.97 |
| ACC | 0.82 | 0.77 | 0.79 | 0.70 | 0.56 | 0.96 | 0.94 | 0.92 | 0.96 | 0.82 |
| MCC | -0.07 | 0.02 | -0.09 | -0.09 | - | - | -0.03 | 0.16 | -0.02 | -0.02 |
| F1 | 0 | 0.08 | 0 | 0 | 0 | 0 | 0 | 0.2 | 0 | 0.02 |

**Reference:**

1. Jacobson, T. A. (2004). Comparative pharmacokinetic interaction profiles of pravastatin, simvastatin, and atorvastatin when coadministered with cytochrome P450 inhibitors. The American Journal of Cardiology, 94(9), 1140–1146. <https://doi.org/10.1016/j.amjcard.2004.07.080>
2. Tornio, A., Pasanen, M. K., Laitila, J., Neuvonen, P. J., & Backman, J. T. (2005). Comparison of 3‐Hydroxy‐3‐methylglutaryl Coenzyme A (HMG‐CoA) Reductase Inhibitors (Statins) as Inhibitors of Cytochrome P450 2C8. Basic & Clinical Pharmacology & Toxicology, 97(2), 104–108. <https://doi.org/10.1111/j.1742-7843.2005.pto_134.x>
3. Cohen, L. H., van Leeuwen, R. E. W., van Thiel, G. C. F., van Pelt, J. F., & Yap, S. H. (2000). Equally potent inhibitors of cholesterol synthesis in human hepatocytes have distinguishable effects on different cytochrome P450 enzymes. Biopharmaceutics & Drug Disposition, 21(9), 353–364. <https://doi.org/10.1002/bdd.249>
4. Korhonova, M., Doricakova, A., & Dvorak, Z. (2015). Optical Isomers of Atorvastatin, Rosuvastatin and Fluvastatin Enantiospecifically Activate Pregnane X Receptor PXR and Induce CYP2A6, CYP2B6 and CYP3A4 in Human Hepatocytes. PloS One, 10(9), e0137720-. <https://doi.org/10.1371/journal.pone.0137720>
5. Gilani, B., and Cassagnol, M. (2021). “Biochemistry, Cytochrome P450,” in StatPearls. Treasure Island (FL): StatPearls Publishing Copyright © 2021 (StatPearls Publishing LLC.).
6. Funck-Brentano, C., Becquemont, L., Lenevu, A., Roux, A., Jaillon, P., & Beaune, P. (1997). Inhibition by omeprazole of proguanil metabolism: mechanism of the interaction in vitro and prediction of in vivo results from the in vitro experiments. The Journal of Pharmacology and Experimental Therapeutics, 280(2), 730–738.
7. Shirasaka, Y., Sager, J. E., Lutz, J. D., Davis, C., & Isoherranen, N. (2013). Inhibition of CYP2C19 and CYP3A4 by omeprazole metabolites and their contribution to drug-drug interactions. Drug Metabolism and Disposition, 41(7), 1414–1424. <https://doi.org/10.1124/dmd.113.051722>
8. Han, X., Ouyang, D., Chen, X., Shu, Y., Jiang, C., Tan, Z., & Zhou, H. (2002). Inducibility of CYP1A2 by omeprazole in vivo related to the genetic polymorphism of CYP1A2. British Journal of Clinical Pharmacology, 54(5), 540–543. <https://doi.org/10.1046/j.1365-2125.2002.01686.x>
9. McCrea, J. B., Cribb, A., Rushmore, T., Osborne, B., Gillen, L., Lo, M., Waldman, S., Bjornsson, T., Spielberg, S., & Goldberg, M. R. (1999). Phenotypic and genotypic investigations of a healthy volunteer deficient in the conversion of losartan to its active metabolite E-3174. Clinical Pharmacology and Therapeutics, 65(3), 348–352. <https://doi.org/10.1016/S0009-9236(99)70114-1>
10. WALSKY, R. L., OBACH, R. S., GAMAN, E. A., GLEESON, J.-P. R., & PROCTOR, W. R. (2005). SELECTIVE INHIBITION OF HUMAN CYTOCHROME P4502C8 BY MONTELUKAST. Drug Metabolism and Disposition, 33(3), 413–418. <https://doi.org/10.1124/dmd.104.002766>
11. PRESKORN, H., GREENBLATT, D. J., FLOCKHART, D., YAN LUO, PERLOFF, E. S., HARMATZ, J. S., BAKER, B., KLICK-DAVIS, A., DESTA, Z., & BURT, T. (2007). Comparison of duloxetine, escitalopram, and sertraline effects on cytochrome P450 2D6 function in healthy volunteers. Journal of Clinical Psychopharmacology, 27(1), 28–34. <https://doi.org/10.1097/00004714-200702000-00005>
12. PRESKORN, H., GREENBLATT, D. J., FLOCKHART, D., YAN LUO, PERLOFF, E. S., HARMATZ, J. S., BAKER, B., KLICK-DAVIS, A., DESTA, Z., & BURT, T. (2007). Comparison of duloxetine, escitalopram, and sertraline effects on cytochrome P450 2D6 function in healthy volunteers. Journal of Clinical Psychopharmacology, 27(1), 28–34. <https://doi.org/10.1097/00004714-200702000-00005>
13. FAUCETTE, S. R., HAWKE, R. L., LECLUYSE, E. L., SHORD, S. S., BINGFANG YAN, LAETHEM, R. M., & LINDLEY, C. M. (2000). Validation of Bupropion Hydroxylation as a Selective Marker of Human Cytochrome P450 2B6 Catalytic Activity. Drug Metabolism and Disposition, 28(10), 1222–1230.
14. Tanaka, M., Ohkubo, T., Otani, K., Suzuki, A., Kaneko, S., Sugawara, K., Ryokawa, Y., Hakusui, H., Yamamori, S., & Ishizaki, T. (1997). Metabolic disposition of pantoprazole, a proton pump inhibitor, in relation to S‐mephenytoin 4′‐hydroxylation phenotype and genotype. Clinical Pharmacology and Therapeutics, 62(6), 619–628. <https://doi.org/10.1016/S0009-9236(97)90081-3>
15. Li, X.-Q., Andersson, T. B., Ahlström, M., & Weidolf, L. (2004). COMPARISON OF INHIBITORY EFFECTS OF THE PROTON PUMP-INHIBITING DRUGS OMEPRAZOLE, ESOMEPRAZOLE, LANSOPRAZOLE, PANTOPRAZOLE, AND RABEPRAZOLE ON HUMAN CYTOCHROME P450 ACTIVITIES. Drug Metabolism and Disposition, 32(8), 821–827. <https://doi.org/10.1124/dmd.32.8.821>
16. Susan Rotzinger, Jian Fang, & Glen B. Baker. (1998). Trazodone Is Metabolized tom-Chlorophenylpiperazine by CYP3A4 from Human Sources. Drug Metabolism and Disposition, 26(6), 572-575.
17. Miners, J. O., & Birkett, D. J. (1998). Cytochrome P4502C9: an enzyme of major importance in human drug metabolism. British Journal of Clinical Pharmacology, 45(6), 525–538. <https://doi.org/10.1046/j.1365-2125.1998.00721.x>
18. Hamelin, B. A., Turgeon, J., Vallée, F., Bélanger, P., Paquet, F., & LeBel, M. (1996). The disposition of fluoxetine but not sertraline is altered in poor metabolizers of debrisoquin. Clinical Pharmacology and Therapeutics, 60(5), 512–521. <https://doi.org/10.1016/S0009-9236(96)90147-2>
19. MCGINNITY, D. F., BERRY, A. J., KENNY, J. R., GRIME, K., & RILEY, R. J. (2006). EVALUATION OF TIME-DEPENDENT CYTOCHROME P450 INHIBITION USING CULTURED HUMAN HEPATOCYTES. Drug Metabolism and Disposition, 34(8), 1291–1300. <https://doi.org/10.1124/dmd.106.009969>
20. Ayano, G. (2016). Psychotropic medications metabolized by cytochromes P450 (CYP1A2) enzyme and relevant drug interactions: Review of articles. Austin J Pharmacol Ther, 4(2), 1085.
21. Venkatakrishnan, K., Greenblatt, D. J., von Moltke, L. L., Schmider, J., Harmatz, J. S., & Shader, R. I. (1998). Five Distinct Human Cytochromes Mediate Amitriptyline N-Demethylation In Vitro: Dominance of CYP 2C19 and 3A4. Journal of Clinical Pharmacology, 38(2), 112–121. <https://doi.org/10.1002/j.1552-4604.1998.tb04399.x>
22. LAINE, K., TYBRING, G., HÄRTTER, S., ANDERSSON, K., SVENSSON, J.-O., WIDEN, J., & BERTILSSON, L. (2001). Inhibition of cytochrome P4502D6 activity with paroxetine normalizes the ultrarapid metabolizer phenotype as measured by nortriptyline pharmacokinetics and the debrisoquin test. Clinical Pharmacology and Therapeutics, 70(4), 327–335. <https://doi.org/10.1067/mcp.2001.118523>
23. DIXON, C. M., COLTHUP, P. V., SERABJIT-SINGH, C. J., KERR, B. M., BOEHLERT, C. C., PARK, G. R., & TARBIT, M. H. (1995). Multiple forms of cytochrome P450 are involved in the metabolism of ondansetron in humans. Drug Metabolism and Disposition, 23(11), 1225–1230.
24. GRANFORS, M. T., BACKMAN, J. T., NEUVONEN, M., & NEUVONEN, P. J. (2004). Ciprofloxacin greatly increases concentrations and hypotensive effect of tizanidine by inhibiting its cytochrome P450 1A2-mediated presystemic metabolism. Clinical Pharmacology and Therapeutics, 76(6), 598–606. <https://doi.org/10.1016/j.clpt.2004.08.018>
25. Otsuka, Y., Poondru, S., Bonate, P. L., Rose, R. H., Jamei, M., Ushigome, F., & Minematsu, T. (2023). Physiologically-based pharmacokinetic modeling to predict drug-drug interaction of enzalutamide with combined P-gp and CYP3A substrates. Journal of Pharmacokinetics and Pharmacodynamics, 50(5), 365–376. <https://doi.org/10.1007/s10928-023-09867-7>
26. Suzuki, K., Yanagawa, T., Shibasaki, T., Kaniwa, N., Hasegawa, R., & Tohkin, M. (2006). Effect of CYP2C9 genetic polymorphisms on the efficacy and pharmacokinetics of glimepiride in subjects with type 2 diabetes. Diabetes Research and Clinical Practice, 72(2), 148–154. <https://doi.org/10.1016/j.diabres.2005.09.019>
27. Otton, S., Gillam, E., Lennard, M., Tucker, G., & Woods, H. (1990). Propranolol oxidation by human liver microsomes‐the use of cumene hydroperoxide to probe isoenzyme specificity and regio‐ and stereoselectivity. British Journal of Clinical Pharmacology, 30(5), 751–760. <https://doi.org/10.1111/j.1365-2125.1990.tb03846.x>
28. Tateishi, T., Ohashi, K., Fujimura, A., & Ebihara, A. (1992). The Influence of Diltiazem Versus Cimetidine on Propranolol Metabolism. Journal of Clinical Pharmacology, 32(12), 1099–1104. <https://doi.org/10.1177/009127009203201207>
29. Dean L. Aripiprazole Therapy and CYP2D6 Genotype. 2016 Sep 22. In: Pratt VM, Scott SA, Pirmohamed M, et al., editors. Medical Genetics Summaries [Internet]. Bethesda (MD): National Center for Biotechnology Information (US); 2012-. <https://www.ncbi.nlm.nih.gov/books/>
30. HUSKEY, S.-E. W., DEAN, D. C., MILLER, R. R., RASMUSSON, G. H., & SHUET-HING LEE CHIU. (1995). Identification of human cytochrome P450 isozymes responsible for the in vitro oxidative metabolism of finasteride. Drug Metabolism and Disposition, 23(10), 1126–1135.
31. Kim, K. Y., & Mancano, M. A. (2003). Fenofibrate Potentiates Warfarin Effects. The Annals of Pharmacotherapy, 37(2), 212–215. <https://doi.org/10.1177/106002800303700210>
32. Werner, U., Werner, D., Rau, T., Fromm, M. F., Hinz, B., & Brune, K. (2003). Celecoxib inhibits metabolism of cytochrome P450 2D6 substrate metoprolol in humans. Clinical Pharmacology and Therapeutics, 74(2), 130–137. <https://doi.org/10.1016/S0009-9236(03)00120-6>
33. Richter, T., Mürdter, T. E., Heinkele, G., Pleiss, J., Tatzel, S., Schwab, M., Eichelbaum, M., & Zanger, U. M. (2004). Potent Mechanism-Based Inhibition of Human CYP2B6 by Clopidogrel and Ticlopidine. The Journal of Pharmacology and Experimental Therapeutics, 308(1), 189–197. <https://doi.org/10.1124/jpet.103.056127>
34. Walsky, R. L., Astuccio, A. V., & Obach, R. S. (2006). Evaluation of 227 Drugs for In Vitro Inhibition of Cytochrome P450 2B6. Journal of Clinical Pharmacology, 46(12), 1426–1438. <https://doi.org/10.1177/0091270006293753>
35. Nicolas, J.-M., Whomsley, R., Collart, P., & Roba, J. (1999). In vitro inhibition of human liver drug metabolizing enzymes by second generation antihistamines. Chemico-Biological Interactions, 123(1), 63–79. <https://doi.org/10.1016/S0009-2797(99)00131-3>
36. Itkonen, M. K., Tornio, A., Neuvonen, M., Neuvonen, P. J., Niemi, M., & Backman, J. T. (2019). Clopidogrel and Gemfibrozil Strongly Inhibit the CYP2C8-Dependent Formation of 3-Hydroxydesloratadine and Increase Desloratadine Exposure In Humans. Drug Metabolism and Disposition, 47(4), 377–385. <https://doi.org/10.1124/dmd.118.084665>
37. Neuvonen, P. J., Niemi, M., & Backman, J. T. (2006). Drug interactions with lipid-lowering drugs: Mechanisms and clinical relevance. Clinical Pharmacology and Therapeutics, 80(6), 565–581. <https://doi.org/10.1016/j.clpt.2006.09.003>
38. Augustin, M., Schoretsanitis, G., Gründer, G., Haen, E., & Paulzen, M. (2018). How to Treat Hypertension in Venlafaxine-Medicated Patients-Pharmacokinetic Considerations in Prescribing Amlodipine and Ramipril. Journal of Clinical Psychopharmacology, 38(5), 498–501. <https://doi.org/10.1097/JCP.0000000000000929>
39. Ajimura, C. M., Jagan, N., Morrow, L. E., & Malesker, M. A. (2018). Drug Interactions With Oral Inhaled Medications. Journal of Pharmacy Technology, 34(6), 273–280. <https://doi.org/10.1177/8755122518788809>
40. BARECKI, M. E., CASCIANO, C. N., JOHNSON, W. W., & CLEMENT, R. P. (2001). In Vitro Characterization of the Inhibition Profile of Loratadine, Desloratadine, and 3-OH-Desloratadine for Five Human Cytochrome P-450 Enzymes. Drug Metabolism and Disposition, 29(9), 1173–1175.
41. Furuta, S., Kamada, E., Suzuki, T., Sugimoto, T., Kawabata, Y., Shinozaki, Y., & Sano, H. (2001). Inhibition of drug metabolism in human liver microsomes by nizatidine, cimetidine and omeprazole. Xenobiotica, 31(1), 1–10. <https://doi.org/10.1080/00498250110035615>
42. Taavitsainen, P., Kiukaanniemi, K., & Pelkonen, O. (2000). In vitro inhibition screening of human hepatic P 450 enzymes by five angiotensin-II receptor antagonists. European Journal of Clinical Pharmacology, 56(2), 135–140. <https://doi.org/10.1007/s002280050731>
43. OBACH, R. S., COX, L. M., & TREMAINE, L. M. (2005). SERTRALINE IS METABOLIZED BY MULTIPLE CYTOCHROME P450 ENZYMES, MONOAMINE OXIDASES, AND GLUCURONYL TRANSFERASES IN HUMAN: AN IN VITRO STUDY. Drug Metabolism and Disposition, 33(2), 262–270. <https://doi.org/10.1124/dmd.104.002428>
44. Schmider, J., Greenblatt, D. J., Von Moltke, L. L., Karsov, D., & Shader, R. I. (1997). Inhibition of CYP2C9 by selective serotonin reuptake inhibitors in vitro: studies of phenytoin p‐hydroxylation. British Journal of Clinical Pharmacology, 44(5), 495–498. <https://doi.org/10.1046/j.1365-2125.1997.00601.x>
45. Röjdmark, S., & von Bahr, C. (2002). Metabolic interaction between psychopharmaceuticals. Probable cause of exacerbation of hypothyroidism according to a case report. Läkartidningen, 99(25), 2854-6.
46. Vermes, A., & Vermes, I. (2004). Genetic polymorphisms in cytochrome P450 enzymes: effect on efficacy and tolerability of HMG-CoA reductase inhibitors. American Journal of Cardiovascular Drugs : Drugs, Devices, and Other Interventions, 4(4), 247–255. <https://doi.org/10.2165/00129784-200404040-00005>
47. Zhou, S.-F., Zhou, Z.-W., Yang, L.-P., & Cai, J.-P. (2009). Substrates, inducers, inhibitors and structure-activity relationships of human Cytochrome P450 2C9 and implications in drug development. Current Medicinal Chemistry, 16(27), 3480–3675. <https://doi.org/10.2174/092986709789057635>
48. Laine, J. E., Auriola, S., Pasanen, M., & Juvonen, R. O. (2009). Acetaminophen bioactivation by human cytochrome P450 enzymes and animal microsomes. Xenobiotica, 39(1), 11–21. <https://doi.org/10.1080/00498250802512830>
49. Feierman, D. E., Melnikov, Z., & Zhang, J. (2002). The Paradoxical Effect of Acetaminophen on CYP3A4 Activity and Content in Transfected HepG2 Cells. Archives of Biochemistry and Biophysics, 398(1), 109–117. <https://doi.org/10.1006/abbi.2001.2677>
50. Chen, F., Liu, H., Sun, H., Pan, P., Li, Y., Li, D., & Hou, T. (2016). Assessing the performance of the MM/PBSA and MM/GBSA methods. 6. Capability to predict protein-protein binding free energies and re-rank binding poses generated by protein-protein docking. Physical Chemistry Chemical Physics : PCCP, 18(32), 22129–22139. <https://doi.org/10.1039/c6cp03670h>
51. Otton, S. V., Schadel, M., Cheung, S. W., Kaplan, H. L., Busto, U. E., & Sellers, E. M. (1993). CYP2D6 phenotype determines the metabolic conversion of hydrocodone to hydromorphone. Clinical Pharmacology and Therapeutics, 54(5), 463–472. <https://doi.org/10.1038/clpt.1993.177>
52. Hutchinson, M. R., Menelaou, A., Foster, D. J. R., Coller, J. K., & Somogyi, A. A. (2004). CYP2D6 and CYP3A4 involvement in the primary oxidative metabolism of hydrocodone by human liver microsomes. British Journal of Clinical Pharmacology, 57(3), 287–297. <https://doi.org/10.1046/j.1365-2125.2003.02002.x>
53. KAMIMURA, H., OISHIOE, S., MATSUSHIMA, H., WATANABE, T., HIGUCHI, S., HALL, M., WOOD, S. G., & CHASSEAUD, L. F. (1998). Identification of cytochrome P450 isozymes involved in metabolism of the α1-adrenoceptor blocker tamsulosin in human liver microsomes. Xenobiotica, 28(10), 909–922. <https://doi.org/10.1080/004982598238985>
54. Mazaleuskaya, L. L., Theken, K. N., Gong, L., Thorn, C. F., FitzGerald, G. A., Altman, R. B., & Klein, T. E. (2015). PharmGKB summary: ibuprofen pathways. Pharmacogenetics and Genomics, 25(2), 96–106. <https://doi.org/10.1097/FPC.0000000000000113>
55. Huang, F., Ajavon, A., Huang, E., Lettieri, J., Liu, R., Peña, C., & Berse, M. (2017). No Effect of Levothyroxine and Levothyroxine-Induced Subclinical Thyrotoxicosis on the Pharmacokinetics of Sorafenib in Healthy Male Subjects. Thyroid (New York, N.Y.), 27(9), 1118–1127. <https://doi.org/10.1089/thy.2017.0085>
56. Rollins, D. E., & Blumenthal, D. K. (2016). Workbook and Casebook for Goodman and Gilman’s : The Pharmacological Basis of Therapeutics. McGraw-Hill Education LLC.
57. Schoretsanitis, G., de Leon, J., Eap, C. B., Kane, J. M., & Paulzen, M. (2019). Clinically Significant Drug–Drug Interactions with Agents for Attention-Deficit/Hyperactivity Disorder. CNS Drugs, 33(12), 1201–1222. <https://doi.org/10.1007/s40263-019-00683-7>
58. Dean, L. (2017). Metoprolol therapy and CYP2D6 genotype
59. Prueksaritanont, T., Ma, B., & Yu, N. (2003). The human hepatic metabolism of simvastatin hydroxy acid is mediated primarily by CYP3A, and not CYP2D6. British Journal of Clinical Pharmacology, 56(1), 120–124. <https://doi.org/10.1046/j.1365-2125.2003.01833.x>
60. Chen, Y., Liu, H., Liu, L., Nguyen, K., Jones, E. B., & Fretland, A. J. (2010). The in vitro metabolism of bupropion revisited: concentration dependent involvement of cytochrome P450 2C19. Xenobiotica, 40(8), 536–546. <https://doi.org/10.3109/00498254.2010.492880>
61. Rendic, S., & Carlo, F. J. D. (1997). Human Cytochrome P450 Enzymes: A Status Report Summarizing Their Reactions, Substrates, Inducers, and Inhibitors. Drug Metabolism Reviews, 29(1–2), 413–580. <https://doi.org/10.3109/03602539709037591>
62. Monostory, K., Hazai, E., & Vereczkey, L. (2004). Inhibition of cytochrome P450 enzymes participating in p-nitrophenol hydroxylation by drugs known as CYP2E1 inhibitors. Chemico-Biological Interactions, 147(3), 331–340. <https://doi.org/10.1016/j.cbi.2004.03.003>
63. Xu, R.-A., Xu, Z.-S., Hu, L.-F., Zhang, C.-H., Pan, X.-F., Shi, D.-W., Ye, X.-L., Liu, Y.-J., & Zhang, X.-H. (2013). Effects of repeated allopurinol administration on rat cytochrome P450 activity. Pharmazie, 68(5), 365–368. <https://doi.org/10.1691/ph.2013.2775>
64. Fohner, A. E., Sparreboom, A., Altman, R. B., & Klein, T. E. (2017). PharmGKB summary: Macrolide antibiotic pathway, pharmacokinetics/pharmacodynamics. Pharmacogenetics and Genomics, 27(4), 164–167. <https://doi.org/10.1097/FPC.0000000000000270>
65. HAMELIN, B. A., BOUAYAD, A., DROLET, B., GRAVEL, A., & TURGEON, J. (1998). In Vitro Characterization of Cytochrome P450 2D6 Inhibition by Classic Histamine H1 Receptor Antagonists. Drug Metabolism and Disposition, 26(6), 536–539.
66. Zhu, Y., Wang, F., Li, Q., Zhu, M., Du, A., Tang, W., & Chen, W. (2014). Amlodipine metabolism in human liver microsomes and roles of CYP3A4/5 in the dihydropyridine dehydrogenation. Drug Metabolism and Disposition, 42(2), 245–249. <https://doi.org/10.1124/dmd.113.055400>
67. <https://www.accessdata.fda.gov/drugsatfda_docs/label/2017/020235s064_020882s047_021129s046lbl.pdf>
68. Stockmann, C., Fassl, B., Gaedigk, R., Nkoy, F., Uchida, D. A., Monson, S., Reilly, C. A., Leeder, J. S., Yost, G. S., & Ward, R. M. (2013). Fluticasone Propionate Pharmacogenetics: CYP3A422 Polymorphism and Pediatric Asthma Control. The Journal of Pediatrics, 162(6), 1222-1227.e2. <https://doi.org/10.1016/j.jpeds.2012.11.031>
69. Zaretzki, J., Bergeron, C., Huang, T., Rydberg, P., Swamidass, S. J., & Breneman, C. M. (2013). RS-WebPredictor: a server for predicting CYP-mediated sites of metabolism on drug-like molecules. Bioinformatics, 29(4), 497–498. <https://doi.org/10.1093/bioinformatics/bts705>
70. Chan, C. Y., New, L. S., Ho, H. K., & Chan, E. C. Y. (2011). Reversible time-dependent inhibition of cytochrome P450 enzymes by duloxetine and inertness of its thiophene ring towards bioactivation. Toxicology Letters, 206(3), 314–324. <https://doi.org/10.1016/j.toxlet.2011.07.019>
71. Olsen, L., Montefiori, M., Tran, K. P., & Jørgensen, F. S. (2019). SMARTCyp 3.0: enhanced cytochrome P450 site-of-metabolism prediction server. Bioinformatics (Oxford, England), 35(17), 3174–3175. <https://doi.org/10.1093/bioinformatics/btz037>
72. Richter, T., Mürdter, T. E., Heinkele, G., Pleiss, J., Tatzel, S., Schwab, M., Eichelbaum, M., & Zanger, U. M. (2004). Potent Mechanism-Based Inhibition of Human CYP2B6 by Clopidogrel and Ticlopidine. The Journal of Pharmacology and Experimental Therapeutics, 308(1), 189–197. <https://doi.org/10.1124/jpet.103.056127>
73. Palikhe, N. S., Kim, S.-H., Nam, Y. H., Ye, Y.-M., & Park, H.-S. (2011). Polymorphisms of Aspirin-Metabolizing Enzymes CYP2C9, NAT2 and UGT1A6 in Aspirin-Intolerant Urticaria. Allergy, Asthma & Immunology Research, 3(4), 273–276. <https://doi.org/10.4168/aair.2011.3.4.273>
74. Chen, X., Tan, Z., Huang, S., Huang, Z., Ou‐Yang, D., & Zhou, H. (2003). Isozyme-specific induction of low-dose aspirin on cytochrome P450 in healthy subjects. Clinical Pharmacology and Therapeutics, 73(3), 264–271. <https://doi.org/10.1067/mcp.2003.14>
75. Sternieri, E., Coccia, C. P. R., Pinetti, D., Guerzoni, S., & Ferrari, A. (2006). Pharmacokinetics and interactions of headache medications, part II: prophylactic treatments. Expert Opinion on Drug Metabolism & Toxicology, 2(6), 981–1007. <https://doi.org/10.1517/17425255.2.6.981>
76. Fogelman, S. M., Schmider, J., Venkatakrishnan, K., von Moltke, L. L., Harmatz, J. S., Shader, R. I., & Greenblatt, D. J. (1999). O- and N-demethylation of Venlafaxine In Vitro by Human Liver Microsomes and by Microsomes from cDNA-Transfected Cells: Effect of Metabolic Inhibitors and SSRI Antidepressants. Neuropsychopharmacology (New York, N.Y.), 20(5), 480–490. <https://doi.org/10.1016/S0893-133X(98)00113-4>
77. Ezuruike, U., Humphries, H., Dickins, M., Neuhoff, S., Gardner, I., & Rowland Yeo, K. (2018). Risk–Benefit Assessment of Ethinylestradiol Using a Physiologically Based Pharmacokinetic Modeling Approach. Clinical Pharmacology and Therapeutics, 104(6), 1229–1239. <https://doi.org/10.1002/cpt.1085>
78. Wang, B., Sanchez, R. I., Franklin, R. B., Evans, D. C., & Huskey, S. E. W. (2004). The involvement of CYP3A4 and CYP2C9 in the metabolism of 17α-ethinylestradiol. Drug metabolism and disposition, 32(11), 1209-1212.
79. Palovaara, S., Tybring, G., & Laine, K. (2003). The effect of ethinyloestradiol and levonorgestrel on the CYP2C19‐mediated metabolism of omeprazole in healthy female subjects. British Journal of Clinical Pharmacology, 56(2), 232–237. <https://doi.org/10.1046/j.1365-2125.2003.01868.x>
80. LALOVIC, B., PHILLIPS, B., RISLER, L. L., HOWALD, W., & SHEN, D. D. (2004). QUANTITATIVE CONTRIBUTION OF CYP2D6 AND CYP3A TO OXYCODONE METABOLISM IN HUMAN LIVER AND INTESTINAL MICROSOMES. Drug Metabolism and Disposition, 32(4), 447–454. <https://doi.org/10.1124/dmd.32.4.447>
81. ZHOU, S.-F., XUE, C. C., YU, X.-Q., CHUNGUANG LI, & GUANGJI WANG. (2007). Clinically Important Drug Interactions Potentially Involving Mechanism-based Inhibition of Cytochrome P450 3A4 and the Role of Therapeutic Drug Monitoring. Therapeutic Drug Monitoring, 29(6), 687–710. <https://doi.org/10.1097/FTD.0b013e31815c16f5>
82. Sun, H., & Scott, D. O. (2010). Structure-based Drug Metabolism Predictions for Drug Design. Chemical Biology & Drug Design, 75(1), 3–17. <https://doi.org/10.1111/j.1747-0285.2009.00899.x>
83. Farid, N. A., Payne, C. D., Small, D. S., Winters, K. J., Ernest, C. S., Brandt, J. T., Darstein, C., Jakubowski, J. A., & Salazar, D. E. (2007). Cytochrome P450 3A Inhibition by Ketoconazole Affects Prasugrel and Clopidogrel Pharmacokinetics and Pharmacodynamics Differently. Clinical Pharmacology and Therapeutics, 81(5), 735–741. <https://doi.org/10.1038/sj.clpt.6100139>
84. MARGOLIS, J. M., O’DONNELL, J. P., MANKOWSKI, D. C., EKINS, S., & OBACH, R. S. (2000). (R)-, (S)-, and Racemic Fluoxetine N-Demethylation by Human Cytochrome P450 Enzymes. Drug Metabolism and Disposition, 28(10), 1187–1191.
85. Frye, R. F., Zgheib, N. K., Matzke, G. R., Chaves‐Gnecco, D., Rabinovitz, M., Shaikh, O. S., & Branch, R. A. (2006). Liver disease selectively modulates cytochrome P450-mediated metabolism. Clinical Pharmacology and Therapeutics, 80(3), 235–245. <https://doi.org/10.1016/j.clpt.2006.05.006>
86. RAO, N. (2007). The clinical pharmacokinetics of escitalopram. Clinical Pharmacokinetics, 46(4), 281–290. <https://doi.org/10.2165/00003088-200746040-00002>
87. Ayano, G. (2016). Psychotropic Medications Metabolized by Cytochromes P450 (CYP) 2D6 Enzyme and Relevant Drug Interactions. Clinical Pharmacology & Biopharmaceutics, 5(4). <https://doi.org/10.4172/2167-065X.1000162>
88. Hasselstrøm, J., & Linnet, K. (2006). IN VITRO STUDIES ON QUETIAPINE METABOLISM USING THE SUBSTRATE DEPLETION APPROACH WITH FOCUS ON DRUG-DRUG INTERACTIONS. Drug Metabolism and Drug Interactions, 21(3–4), 187–212. <https://doi.org/10.1515/DMDI.2006.21.3-4.187>
